# Canonicalizing Zeta Generators: Genus Zero and Genus One

**DOI:** 10.1007/s00220-025-05489-x

**Published:** 2025-12-08

**Authors:** Daniele Dorigoni, Mehregan Doroudiani, Joshua Drewitt, Martijn Hidding, Axel Kleinschmidt, Oliver Schlotterer, Leila Schneps, Bram Verbeek

**Affiliations:** 1 Centre for Particle Theory and Department of Mathematical Sciences, Durham University, Lower Mountjoy, Stockton Road, Durham, DH1 3LE UK; 2 Max-Planck-Institut für Gravitationsphysik (Albert-Einstein-Institut), Am Mühlenberg 1, 14476 Potsdam, Germany; 3 School of Mathematics, University of Bristol, Queens Road, Bristol, BS8 1QU UK; 4 Department of Physics and Astronomy, Uppsala University, 75108 Uppsala, Sweden; 5 Institute for Theoretical Physics, ETH Zurich, 8093 Zurich, Switzerland; 6 Department of Mathematics, Centre for Geometry and Physics, Uppsala University, 75106 Uppsala, Sweden; 7 International Solvay Institutes, ULB-Campus Plaine, CP231, 1050 Brussels, Belgium; 8 CNRS, Sorbonne Université, Campus Pierre et Marie Curie, 4 Place Jussieu, 75005 Paris, France

## Abstract

Zeta generators are derivations associated with odd Riemann zeta values that act freely on the Lie algebra of the fundamental group of Riemann surfaces with marked points. The genus-zero incarnation of zeta generators are Ihara derivations of certain Lie polynomials in two generators that can be obtained from the Drinfeld associator. We characterize a canonical choice of these polynomials, together with their non-Lie counterparts at even degrees $w\ge 2$, through the action of the dual space of formal and motivic multizeta values. Based on these canonical polynomials, we propose a canonical isomorphism that maps motivic multizeta values into the *f*-alphabet. The canonical Lie polynomials from the genus-zero setup determine canonical zeta generators in genus one that act on the two generators of Enriquez’ elliptic associators. Up to a single contribution at fixed degree, the zeta generators in genus one are systematically expanded in terms of Tsunogai’s geometric derivations dual to holomorphic Eisenstein series, leading to a wealth of explicit high-order computations. Earlier ambiguities in defining the non-geometric part of genus-one zeta generators are resolved by imposing a new representation-theoretic condition. The tight interplay between zeta generators in genus zero and genus one unravelled in this work connects the construction of single-valued multiple polylogarithms on the sphere with iterated-Eisenstein-integral representations of modular graph forms.

## Introduction

A wealth of recent interactions between mathematics and physics evolves around the appearance of multizeta values in period integrals and scattering amplitudes. In the first place, multizeta values arise as real numbers defined by the infinite sums

1.1 $$\begin{aligned} \zeta _{k_1,k_2,\ldots ,k_r} :=\sum _{1\le n_1<n_2<\cdots <n_r}^{\infty } n_1^{-k_1} n_2^{-k_2}\ldots n_r^{-k_r} , \end{aligned}$$

where $k_1,\ldots ,k_r \in {\mathbb {N}}$ and $k_r>1$ in order to ensure convergence of the sum. While many of their number-theoretic properties, including transcendentality of Riemann zeta values, remain conjectural at the level of real numbers, there are rigorous results on their motivic versions.^1^ In particular, motivic multizeta values admit a Hopf algebra structure which was observed to universally govern quantum-field-theory and string-theory amplitudes. These structures also facilitate and guide many concrete investigations of period computations. In this article we set forth some canonical features of motivic multizeta values at the level of their Hopf algebra comodule ${\mathcal{M}\mathcal{Z}}$ and its graded dual.

Both practical computations and structural understanding of multizetas benefit from organizing period integrals into generating series. The non-commuting bookkeeping variables of these generating series can be identified with generators of certain Lie algebras associated with punctured Riemann surfaces of different genus. A central ingredient of generating-series approaches to multizeta values are *zeta generators* that have been studied before from different angles in the literature but whose definition depended on certain ad-hoc choices.

In this work, we present canonical zeta generators in genus zero and genus one. Furthermore, our results also imply a canonical map from motivic multizeta values to the so-called *f*-alphabet [2, 4], a representation of ${\mathcal{M}\mathcal{Z}}$ that is widely used but has eluded a canonical form until this work. The methods we present in this work are fully constructive.

One of the main motivations for seeking concrete expressions for zeta generators stems from the key role they play for the construction of single-valued multiple polylogarithms on the sphere [5–8] and of modular equivariant iterated integrals of Eisenstein series [9–12]. In a physics context, our results on zeta generators facilitate and organize the low-energy expansion of perturbative string-theory scattering amplitudes [13–17]. In particular, the contributions from string world-sheets of genus zero and one are intertwined through the connection between the associated zeta generators, bringing out universal structures of importance also for string dualities.

### The canonical zeta generators in genus zero

Our first main contribution is the definition of a canonical set of generators for the graded dual ${\mathcal{M}\mathcal{Z}}^\vee$ of the Hopf algebra comodule ${\mathcal{M}\mathcal{Z}}$ of motivic multizeta values. These canonical generators will be encoded in a family of homogeneous degree-*w* polynomials

1.2 $$\begin{aligned} g_w(x,y)\in {\mathbb {Q}}\langle x,y\rangle ,\ \ \ \ w\ge 2 \end{aligned}$$

in two non-commutative variables *x*, *y*. The polynomials $g_w$ satisfy three natural conditions presented in Theorem 1.1.1 below and related to the intrinsic structure of ${\mathcal{M}\mathcal{Z}}$, see Remark 1.1.2 and (3.32) for examples. For odd values of *w*, the polynomials $g_w$ are Lie polynomials which provide a set of canonical generators for the genus zero motivic Lie algebra which is well-known to be a free Lie algebra with one generator in each odd degree $w\ge 3$ (a result established in [18]).^2^ This freeness property is essential throughout this work but only established for the Lie-algebra structure underlying *motivic* multizetas as opposed to the incarnation (1.1) of multizetas as real numbers. Hence, our main results are stated only for *motivic* multizetas, although they are expected to apply to real multizetas (1.1) in identical form by the conjectural isomorphism between real and motivic multizetas.

The key tool used to define the polynomials $g_w$ is the *Z-map*, first introduced in [20] and explained here in Sect. 3, which is a canonical linear isomorphism from ${\mathcal{M}\mathcal{Z}}^\vee$ to ${\mathcal{M}\mathcal{Z}}$. The Z-map comes from the canonical isomorphism of vector spaces

1.3 $$\begin{aligned} {\mathbb {Q}}\langle x,y\rangle \rightarrow {\mathbb {Q}}[Z(w)], \end{aligned}$$

where the space on the right-hand side is the ${\mathbb {Q}}$-vector space on symbols *Z*(*w*) indexed by all monomials *w* in the letters *x*, *y*, and the isomorphism is given simply by mapping $w\mapsto Z(w)$. Identifying ${\mathbb {Q}}\langle x,y\rangle$ with the dual space of ${\mathbb {Q}}[Z(w)]$ and considering the bases of monomials *w* and of symbols *Z*(*w*) as dual bases makes this into an isomorphism of dual vector spaces. We will also use variants of the Z-map associated with different quotients of ${\mathbb {Q}}[Z(w)]$ or subspaces of ${\mathbb {Q}}\langle x,y\rangle$. One such quotient is given by ${\mathcal{M}\mathcal{Z}}$, obtained by imposing the linear relations between motivic multizeta values on the symbols *Z*(*w*).

Let ${\mathfrak {mz}}$ denote the quotient of ${\mathcal{M}\mathcal{Z}}$ modulo the linear subspace spanned by constants, non-trivial products of motivic multizeta values, and the motivic single zeta value $\zeta ^{\mathfrak {m}}_2$. Then, ${\mathfrak {mz}}$ inherits the structure of a Lie coalgebra from the Hopf-algebra-comodule structure of ${\mathcal{M}\mathcal{Z}}$ (cf. Sect.  2.2.2). Let ${\mathfrak {mz}}^\vee \subset {\mathcal{M}\mathcal{Z}}^\vee \subset {\mathbb {Q}}\langle x,y\rangle$ denote its dual space, which is a Lie algebra equipped with the Ihara bracket below (see also Sect.  3.1). Like ${\mathcal{M}\mathcal{Z}}$ and ${\mathcal{M}\mathcal{Z}}^\vee$, the spaces ${\mathfrak {mz}}$ and ${\mathfrak {mz}}^\vee$ are graded by the (homogeneity) degree in *x*, *y* or *weight*, with finite-dimensional graded parts for fixed weight. A major structure theorem by Brown [2] has shown that ${\mathfrak {mz}}^\vee$ is freely generated by one Lie polynomial (of depth 1 in the sense of Definition 3.3.1) in each odd homogeneous weight $w\ge 3$.

The universal enveloping algebra ${{\mathcal {U}}}{\mathfrak {mz}}^\vee$ is freely generated by the generators of ${\mathfrak {mz}}^\vee$ under the Poincaré–Birkhoff–Witt multiplication, which we denote by $\diamond$. In the case where $g\in {\mathfrak {mz}}^\vee$ and $h\in \mathcal{U}{\mathfrak {mz}}^\vee$, this multiplication rule has a simple form:

1.4 $$\begin{aligned} g\diamond h=gh+D_g(h) , \end{aligned}$$

where $D_g$ is the Ihara derivation of ${\mathbb {Q}}\langle x,y\rangle$ defined by $D_g(x)=0$ and $D_g(y)=[y,g]$. The space ${\mathcal{M}\mathcal{Z}}^\vee$ is a module over the Hopf algebra ${{\mathcal {U}}}{\mathfrak {mz}}^\vee$.

Let us write ${\mathfrak {mz}}^\vee _{\ge 2}$ for the subspace of ${\mathfrak {mz}}^\vee$ spanned by Ihara brackets $\{ g,h\} :=g\diamond h - h\diamond g$ of the generators; this is a canonical subspace independent of any actual choice of generators. The spaces ${\mathcal{M}\mathcal{Z}}$, ${\mathcal{M}\mathcal{Z}}^\vee$, ${\mathfrak {mz}}$, ${\mathfrak {mz}}^\vee$ and ${\mathfrak {mz}}^\vee _{\ge 2}$ are all weight-graded spaces; we write ${\mathcal{M}\mathcal{Z}}_w$, ${\mathcal{M}\mathcal{Z}}^\vee _w$ etc. to indicate their graded parts of weight *w*, all of which are finite-dimensional. Each graded piece ${\mathcal{M}\mathcal{Z}}_w$ contains a canonical *reducible* subspace ${\hat{R}}_w$ spanned by all weight-*w* products of lower-weight multizeta values. We write $R_w :={\hat{R}}_w$ if *w* is odd, and if *w* is even we let $R_w$ denote the subspace of ${\hat{R}}_w$ spanned by all products except for $(\zeta _2^\mathfrak {m})^{w/2}$, so that

1.5 $$\begin{aligned} {\left\{ \begin{array}{ll} {\hat{R}}_w=R_w& \hbox {if }w\hbox { is odd} ,\\ {\hat{R}}_w={\mathbb {Q}}\zeta ^{\mathfrak {m}}_w\oplus R_w& \hbox {if }w\hbox { is even} , \end{array}\right. } \end{aligned}$$

where $\zeta ^{\mathfrak {m}}_w$ denotes the single zeta value in weight *w*. We then have ${\mathfrak {mz}}_w={\mathcal{M}\mathcal{Z}}_w/{\hat{R}}_w$ for $w\ge 3$. We further define canonical subspaces of *irreducible* multizeta values (resp. non-single irreducible multizeta values) in ${\mathcal{M}\mathcal{Z}}_w$ for each weight $w\ge 2$ by setting

1.6 $$\begin{aligned} {\hat{I}}_w :=Z({\mathfrak {mz}}^\vee _w) ,\ \ \ \ I_w :=Z\bigl (({\mathfrak {mz}}^\vee _{\ge 2})_w\bigr ) , \end{aligned}$$

where we note that

1.7 $$\begin{aligned} {\left\{ \begin{array}{ll} {\hat{I}}_w=I_w& \hbox {if }w\hbox { is even} , \\ {\hat{I}}_w={\mathbb {Q}}\zeta ^{\mathfrak {m}}_w\oplus I_w& \hbox {if }w\hbox { is odd} . \end{array}\right. } \end{aligned}$$

In this way, we obtain a canonical decomposition of ${\mathcal{M}\mathcal{Z}}_w$ into single, irreducible and reducible parts:

1.8 $$\begin{aligned} {\mathcal{M}\mathcal{Z}}_w={\mathbb {Q}}\zeta ^{\mathfrak {m}}_w\oplus I_w\oplus R_w \hspace{10mm}\text {for all }w\ge 2 . \end{aligned}$$

#### Examples

While for all $w\le 7$ the irreducible parts are trivial, e.g.

1.9 $$\begin{aligned} {\mathcal{M}\mathcal{Z}}_5={\mathbb {Q}}\zeta _5^{\mathfrak {m}}\oplus R_5 = \langle \zeta _5^{\mathfrak {m}}\rangle \oplus \langle \zeta _2^{\mathfrak {m}}\zeta _3^{\mathfrak {m}}\rangle , \end{aligned}$$

for $w\ge 8$ we have that generically $I_w\ne \emptyset$. The first non-trivial instance of this decomposition is

1.10 $$\begin{aligned} {\mathcal{M}\mathcal{Z}}_8&=\langle \zeta _8^{\mathfrak {m}}\rangle \oplus \langle Z_{35}\rangle \oplus \langle \zeta _3^{\mathfrak {m}}\zeta _5^{\mathfrak {m}} ,\ \zeta _2^{\mathfrak {m}}(\zeta _3^{\mathfrak {m}})^2\rangle ={\mathbb {Q}}\zeta _8^{\mathfrak {m}}\oplus I_8\oplus R_8 , \end{aligned}$$

where the canonical choice of irreducible mutizeta,

1.11 $$\begin{aligned} Z_{35}&:=Z(\{g_3,g_5\})= -\tfrac{1105181}{80}\zeta _8^{\mathfrak {m}} +\tfrac{24453}{5}\zeta _{3,5}^{\mathfrak {m}} +\tfrac{28743}{2}\zeta _3^{\mathfrak {m}}\zeta _5^{\mathfrak {m}}-1683\,\zeta _2^{\mathfrak {m}}(\zeta _3^{\mathfrak {m}})^2 , \end{aligned}$$

is dictated by the procedure described above. We refer to Sect. 3.4 for more details and examples at higher weight.

The explicit form of the canonical polynomials $g_w$ can be obtained from the motivic Drinfeld associator [21, 22]

1.12 $$\begin{aligned} \Phi ^{\mathfrak {m}}_{\textrm{KZ}}(x,y)\in {\mathbb {Q}}\langle \langle x,y\rangle \rangle \otimes _{\mathbb {Q}}{\mathcal{M}\mathcal{Z}}, \end{aligned}$$

which for our work can be thought of as a generating series of motivic multizeta values [23]. For convenience, we work with the motivic power series $\Phi ^{\mathfrak {m}}(x,y) :=\Phi ^{\mathfrak {m}}_{\textrm{KZ}}(x,-y)$. Apart from the definition of the Z-map and the canonical decomposition (1.8), the main results of Sects. 2 and 3 are summarized by:

#### Theorem 1.1.1

Write the expansion of $\Phi ^{\mathfrak {m}}$ in *x*, *y* in any basis of motivic multizetas adapted to the canonical decomposition (1.8), and for each $w\ge 2$, set

1.13 $$\begin{aligned} g_w :=\Phi ^{\mathfrak {m}}|_{\zeta ^{\mathfrak {m}}_w} . \end{aligned}$$

Then the polynomials $g_w$ lie in ${\mathcal{M}\mathcal{Z}}_w^\vee$. Equivalently, $g_w$ can be identified (with no reference to $\Phi ^{\mathfrak {m}}$) as the unique polynomial in ${\mathcal{M}\mathcal{Z}}_w^\vee$ satisfying the following three properties:

(i) $\langle g_w,\zeta ^{\mathfrak {m}}_w\rangle =1$, where $\langle \cdot , \cdot \rangle$ denotes the canonical action of the dual space ${\mathcal{M}\mathcal{Z}}^\vee$ on ${\mathcal{M}\mathcal{Z}}$ (see (3.1) below),
(ii) $g_w$ annihilates the reducible subspace $R_w\subset {\mathcal{M}\mathcal{Z}}_w$,
(iii) $Z(g_w)\in {\mathbb {Q}}\zeta ^{\mathfrak {m}}_w\oplus R_w$, i.e. it does not contain any irreducible multizeta values in $I_w$.

The $g_w$ for odd $w\ge 3$ form a canonical set of generators for the Lie algebra ${\mathfrak {mz}}^\vee$, and the $g_w$ for all $w\ge 2$ form a set of generators for the Hopf algebra module ${\mathcal{M}\mathcal{Z}}^\vee$ over the Hopf algebra ${{\mathcal {U}}}{\mathfrak {mz}}^\vee$. More precisely, every element of ${\mathcal{M}\mathcal{Z}}^\vee$ can be written uniquely as a product

1.14 $$\begin{aligned} g_{w_1}\diamond \cdots \diamond g_{w_r}\diamond g_k , \end{aligned}$$

where the $w_i$ are all odd $\ge 3$ and $k\ge 2$, and the multiplication proceeds from right to left using the rule (1.4).

#### Remark 1.1.2

For both even and odd $w\ge 2$, the polynomials $g_w$ are canonical since the subspaces $R_w, I_w$ in part (ii) and (iii) of Theorem 1.1.1 are. Their simplest instances are given by $g_2 = [x,y]$ and $g_3 = [x-y,[x,y]]$, with more examples in (3.32). The ancillary files of the arXiv submission of this work contain the explicit form of all $g_w$ with $w\le 12$.

#### Remark 1.1.3

In [24], Écalle gave an alternative method to specify a depth 1 polynomial $g_w$ in each odd weight *w*, by defining an inner product on monomials and then choosing $g_w$ to be the unique element orthogonal to all elements in the associated graded Lie algebra. Using the language of moulds reviewed in Sect. 6.1, he gave two different possibilities for inner products with good symmetry properties (called *kya* and *kwa*, cf. section 19 of [24]).

#### Remark 1.1.4

In his Ph.D. thesis [25], Keilthy gave a similar method, but using the “trivial” inner product on ${\mathbb {Q}}\langle x,y\rangle$ (for which the inner product of two monomials *u* and *v* is $\delta _{u,v}$). This is analogous to our use of the action of the dual space ${\mathbb {Q}}\langle x,y\rangle ^\vee$ on ${\mathbb {Q}}\langle x,y\rangle$ described in Sect. 3.1 below (cf. (3.1)). As we explain in more detail in Sect. 3.3, this construction agrees with ours for odd *w*; however our definition applies uniformly to produce canonical elements for both odd and also for even *w*.

### The canonical *f*-alphabet isomorphism

Brown proved in [2, 4] that the motivic multizeta algebra ${\mathcal{M}\mathcal{Z}}$ is isomorphic to a certain Hopf-algebra comodule ${{\mathcal {F}}}$, known as the *f*-alphabet algebra, which has a very simple structure: it is a commutative algebra under the shuffle multiplication, multiplicatively generated by all monomials in an alphabet of letters $f_2$ and $f_3,f_5,f_7,\ldots$ which is free apart from the unique relation that $f_2$ commutes with all the other letters; thus we have

1.15 $$\begin{aligned} {{\mathcal {F}}}={\mathbb {Q}}[f_2]\otimes _{\mathbb {Q}}\overline{{\mathcal {F}}} , \end{aligned}$$

where $\overline{{\mathcal {F}}}$ is freely generated under the shuffle multiplication by all monomials in $f_3,f_5,\ldots$, and we sometimes write $f_{2n} = \frac{\zeta _{2n}}{(\zeta _2)^n} f_2^n$ for $n \in {\mathbb {N}}$. The space $\overline{{\mathcal {F}}}$ is a commutative Hopf algebra equipped with the shuffle multiplication and the deconcatenation coproduct, and ${{\mathcal {F}}}$ is a Hopf-algebra comodule equipped with the following extension of the deconcatenation coproduct to a coaction:

1.16 $$\begin{aligned} \Delta :{{\mathcal {F}}}&\rightarrow {{\mathcal {F}}}\otimes \overline{{\mathcal {F}}} ,\nonumber \\ f_2^nf_{w_1}\cdots f_{w_r}&\mapsto \sum _{i=0}^r f_2^nf_{w_1}\cdots f_{w_i}\otimes f_{w_{i+1}}\cdots f_{w_r} . \end{aligned}$$

In [2, 4], Brown identified the complete family of Hopf-algebra-comodule isomorphisms ${\mathcal{M}\mathcal{Z}}\rightarrow \mathcal{F}$ normalized by $\zeta ^{\mathfrak {m}}_w\mapsto f_w$, showing that it is parametrized by rational parameters indexed by any basis of non-single irreducible multizetas. In Sect. 4, we display a canonical choice of one such isomorphism, uniquely determined as follows.

#### Theorem 1.2.1

There exists a canonical normalized Hopf algebra comodule isomorphism $\rho :{\mathcal{M}\mathcal{Z}}\rightarrow {{\mathcal {F}}}$ whose definition depends only on the canonical decomposition (1.8); it is characterized by each of the two following properties, which are equivalent:

- $$\rho $$ satisfies
  1.17 $$\begin{aligned} \rho (\xi )|_{f_w}=0\ \ \ \ \forall \ \xi \in I_w , \end{aligned}$$
- if $\Phi ^{\mathfrak {m}}$ is written in a basis adapted to the canonical decomposition (1.8), then $\rho$ satisfies
  1.18 $$\begin{aligned} \rho (\Phi ^{\mathfrak {m}})|_{f_w}=g_w\ \ \ \ \forall \ w \ge 2 . \end{aligned}$$

This choice of isomorphism $\rho$ is canonical since the subspaces $I_w$ and the polynomials $g_w$ in (1.17) and (1.18) are.

#### Example

The irreducible multizeta value of (1.11) has the following *f*-alphabet image:

1.19 $$\begin{aligned} \rho (Z_{35})&=-\tfrac{20163}{2}f_3f_5+\tfrac{28743}{2}f_5f_3-3366f_2f_3f_3 . \end{aligned}$$

The canonical choice of isomorphism is reflected in the absence of a term proportional to $f_8$.

### The canonical zeta generators in genus one

Sections 5 to 7 are dedicated to zeta generators $\sigma _w$ in genus one. These are derivations of the free graded Lie algebra $\textrm{Lie}[a,b]$ associated to the (pro-unipotent) fundamental group of the once-punctured torus. Based on earlier work in [26–29], the action of the genus one generators $\sigma _w$ on *a*, *b* is determined in Sect. 5.4 from the genus-zero polynomials $g_w$ via (with $\textrm{B}_n$ the $n^{\textrm{th}}$ Bernoulli number)

1.20 $$\begin{aligned} \sigma _w(s_{12})&=0 ,&\sigma _w(s_{01})&= \big [ s_{01} , g_w(s_{12},- s_{01})\big ] , \nonumber \\ s_{12}&= [b,a] ,&s_{01}&= -b-\sum _{n\ge 1} \frac{\textrm{B}_n}{n!}\textrm{ad}_a^n(b), \end{aligned}$$

together with the “extension lemma” 2.1.2 of [29] reviewed in Sect. 5.3. In view of the canonical $g_w$ in the defining equation (1.20), we arrive at the first canonical choice of the zeta generators $\sigma _w$ in genus one at arbitrary odd $w\ge 3$.

Another important family of derivations on $\textrm{Lie}[a,b]$ are Tsunogai’s $\epsilon _{k}$ in even degree $k\ge 0$ (i.e. the combined homogeneity degrees in *a* and *b*). The algebra $\mathfrak {u}$ generated by the $\epsilon _k$ is called the algebra of *geometric derivations*. By work of Hain–Matsumoto [27], the $\sigma _w$ normalize the algebra $\mathfrak {u}$, i.e. commutators $[\sigma _w,\mathfrak {u}]$ are again contained in $\mathfrak {u}$. In fact, upon decomposing the zeta generators $\sigma _w$ into an infinite number of contributions at fixed even degree $\ge w+1$, all the terms lie in $\mathfrak {u}$ except for certain contributions at *key degree* 2*w*. The terms of $\sigma _w$ outside $\mathfrak {u}$ belong to yet another derivation known as the *arithmetic part* $z_w$ that furnishes a one-dimensional representation under the $\mathfrak {sl}_2$ spanned by the $\textrm{Lie}[a,b]$-derivations $\epsilon _0, \epsilon _0^\vee$ and $\textrm{h}:=[\epsilon _0, \epsilon _0^\vee ]$ defined by

1.21 $$\begin{aligned} \epsilon _0(a) = b , \ \ \ \ \epsilon _0(b) = 0 , \ \ \ \ \epsilon _0^\vee (a) = 0 , \ \ \ \ \epsilon _0^\vee (b) = a . \end{aligned}$$

Even with the canonical definition of $\sigma _w$, the arithmetic derivations $z_w$ are not entirely characterized by requiring that they form an $\mathfrak {sl}_2$ singlet and that $\sigma _w-z_w \in \mathfrak {u}$. We arrive at canonical representatives of $z_w$ by additionally imposing that they exhaust the complete $\mathfrak {sl}_2$ singlet at key degree of $\sigma _w$. More specifically, the $\epsilon _k^{(j)} :=\textrm{ad}_{\epsilon _0}^j(\epsilon _k)$ with $j=0,1,\ldots ,k{-}2$ composing $\sigma _w{-}z_w$ fall into $(k{-}1)$-dimensional representations of $\mathfrak {sl}_2$ because of $\epsilon _k^{(k-1)} \!=\! 0$. The canonical arithmetic derivations $z_w$ are then uniquely defined by imposing that any nested commutator $\epsilon _k^{(j)}$ at the key degree of $\sigma _w-z_w$ belongs to $\mathfrak {sl}_2$ representations of dimension $\ge 3$.

Based on mould theory, we describe a first algorithm in Sect.  6.2 to explicitly compute the action of $\sigma _w$ on *a* and *b* degree by degree and prove the following theorem:

#### Theorem 1.3.1

(see Theorem 5.4.1 (iii)). The genus-one zeta generators $\sigma _w$ are entirely determined by their parts of degree $<2w$.

This remarkable property of $\sigma _w$ can be combined with the commutation relation [27]

1.22 $$\begin{aligned} {[}N,\sigma _w]= 0 \ \ \textrm{with} \ \ N:=-\epsilon _0 + \sum _{k=2}^\infty (2k-1) \frac{\textrm{B}_{2k}}{(2k)!} \epsilon _{2k} , \end{aligned}$$

to make $\sigma _w$ computationally accessible to all degrees. By solving (1.22) for $[\epsilon _0,\sigma _w]$, it relates contributions to $\sigma _w-z_w$ with different numbers of $\epsilon _{k_i}^{(j_i)}$ factors (with $0\le j_i \le k_i-2$) to be referred to as *modular depth*.^3^ On these grounds, we describe a second algorithm in Sect. 7.3 based on (1.22) to determine $\sigma _w-z_w$ recursively in modular depth, up to highest-weight vectors of $\mathfrak {sl}_2$ in each step which are defined to lie in the kernel of $\textrm{ad}_{\epsilon _0}$. We will infer from the results of [27] that there are no highest-weight vectors beyond key degree. From the viewpoint of (1.22), it is thus sufficient to know the degree $\le 2w$ parts (though Theorem 1.3.1 even guarantees that the complete information is available from degree $< 2w$) of $\sigma _w$. The infinity of terms at degree $\ge 2w+2$ follows from (1.22) together with representation theory of $\mathfrak {sl}_2$.

This setup leads us to present a closed all-degree formula for $\sigma _w$ up to contributions in $\mathfrak {u}$ of modular depth $\ge 3$ (in the ellipsis),

1.23 $$\begin{aligned} \sigma _w&= z_w - \frac{1}{(w-1)!} \epsilon _{w+1}^{(w-1)} \nonumber \\&\quad -\frac{1}{2} \sum _{d=3}^{w-2} \frac{ \textrm{BF}_{d-1} }{\textrm{BF}_{w-d+2} } \sum _{k=d+1}^{w-1} \textrm{BF}_{k-d+1} \textrm{BF}_{w-k+1} s^d(\epsilon _k,\epsilon _{w-k+d}) \nonumber \\&\quad - \sum _{d=5}^w \textrm{BF}_{d-1} s^d(\epsilon _{d-1},\epsilon _{w+1}) - \frac{1}{2} \textrm{BF}_{w+1} s^{w+2}(\epsilon _{w+1},\epsilon _{w+1}) \nonumber \\&\quad + \sum _{k=w+3}^\infty \textrm{BF}_k \sum _{j=0}^{w-2} \frac{(-1)^j \left( {\begin{array}{c}k{-}2\\ j\end{array}}\right) ^{-1} }{j! (w{-}2{-}j)! } \, [ \epsilon _{w+1}^{(w-2-j)} , \epsilon _k^{(j)} ] +\ldots , \end{aligned}$$

where we employ the shorthand $\textrm{BF}_{k}:=\frac{\textrm{B}_k}{k!}$ and we define

1.24 $$\begin{aligned} s^d(\epsilon _{k_1},\epsilon _{k_2}) :=\frac{(d{-}2)! }{(k_1{-}2)! (k_2{-}2)!} \sum _{i=0}^{d-2} (-1)^i [ \epsilon _{k_1}^{(k_1-2-i)}, \epsilon _{k_2}^{(k_2-d+i)}] . \end{aligned}$$

The highest-weight-vector contribution $\sim \epsilon _{w+1}^{(w-1)}$ in first line of (1.23) is well-known and is used to determine the modular-depth two terms in the third and fourth line from (1.22). The second line of (1.23) is conjectural and features highest-weight vectors $s^d(\epsilon _k,\epsilon _{w-k+d})$ in each term—they are not fixed by (1.22) and confirmed by direct computation in a large number of examples. Moreover, the $d=3$ terms in the second line of (1.23) reproduce the closed formula of Brown [33] on depth-three terms in the terminology of the reference.

#### Examples

As an illustration of Theorem 1.3.1, the first two zeta generators are determined fully by the following terms of their expansion in (1.23) bounded by the respective key degrees 6 and 10,

1.25 $$\begin{aligned} \sigma _3 = z_3- \frac{1}{2} \epsilon _4^{(2)} +\ldots , \hspace{10mm} \sigma _5 = z_5 -\frac{1}{24} \epsilon _6^{(4)} -\frac{5 }{48} [\epsilon _4^{(1)},\epsilon _4^{(2)}]+\ldots . \end{aligned}$$

We refer to Sect. 7 for more examples.

Finally, (1.22) together with the terms of modular depth *d* in $\sigma _w - z_w$ fix the explicit form of $[z_w, \epsilon _k] \in \mathfrak {u}$ up to and including modular depth $d+1$. Accordingly, the closed formula (1.23) determines the terms of modular depth three beyond the well-known contributions [27]

1.26 $$\begin{aligned} {[}z_w, \epsilon _k] = \frac{\textrm{BF}_{w+k-1}}{\textrm{BF}_k} \sum _{i=0}^{w-1} \frac{ (-1)^i(k+i-2)!}{i!(w+k-3)!} [\epsilon _{w+1}^{(i)}, \epsilon _{ w+k -1 }^{(w-i-1)} ] +\ldots \end{aligned}$$

and we give closed formulae for $[z_3,\epsilon _k]$ and $[z_5,\epsilon _k]$ at modular depth three in Sect. 7.4.2.

### Motivation and outlook

A major motivation for our study of zeta generators stems from their relevance for periods of configuration spaces of Riemann surfaces with marked points. In genus zero, the canonical polynomials $g_w$ take center stage in the recent reformulation [8] of the motivic coaction [1, 2, 34] and the single-valued map [5–7] of multiple polylogarithms on the sphere. The genus-one zeta generators $\sigma _w$ and their interplay with geometric derivations $\epsilon _k$ unlocked a fully explicit generating-series description of non-holomorphic modular forms in a companion paper [12] to this work.

As detailed in [12], the expansion of $\sigma _w$ in terms of the geometric derivations $\epsilon _k$ determines the appearance of (single-valued) multizeta values in so-called modular graph forms [13, 14]. The latter are non-holomorphic modular forms appearing in genus-one string scattering amplitudes. At a computational level, the precise expressions for $\sigma _w$ in terms of $\epsilon _k$ presented in this work are crucial for an explicit realization of Brown’s construction of non-holomorphic modular forms in [9, 10] which was related to modular graph forms in [11]. At a conceptual level, the intimate connection between zeta generators in genus zero and genus one presented in Sect. 5 leads to a unified description of the single-valued map of multiple polylogarithms in one variable and iterated Eisenstein integrals [12].

These applications of zeta generators in genus zero and genus one lead us to expect that generalizations thereof to compact Riemann surfaces of arbitrary genus with any number of marked points may in fact exist. Our work sets the stage for two lines of follow-up research:

- adapting zeta generators in genus one to systematic constructions of single-valued elliptic polylogarithms pioneered by Zagier [35] in any number of variables and which were more recently approached in the framework of “elliptic modular graph forms” in the string-theory literature [36–39];
- determining higher-genus incarnations of zeta generators from degenerations of the flat connections [40–43] used for constructions of polylogarithms on Riemann surfaces of arbitrary genus and applying them to non-holomorphic modular graph forms [36, 44–47] and tensors [48–51].

## Background on Multizeta Values

In this section, we review basic definitions on different types of multizeta values, their relations and their Hopf-algebraic properties. See [52, 53] for textbook introductions to the subject and [2, 54] for earlier references.

### Real and formal multizeta values

#### Real multizeta values, shuffle and stuffle multiplication

The *real multizeta values* are defined by the infinite sums

2.1 $$\begin{aligned} \zeta _{k_1,k_2,\ldots ,k_r} :=\sum _{1\le n_1<n_2<\ldots <n_r}^{\infty } n_1^{-k_1} n_2^{-k_2}\ldots n_r^{-k_r} , \end{aligned}$$

where $k_1,\ldots ,k_r \in {\mathbb {N}}$ and $k_r>1$ in order to ensure convergence of the sum. The integers *r* and $\sum _{i=1}^r k_i$ in (2.1) are respectively referred to as the *depth* and *weight* of $\zeta _{k_1,k_2,\ldots ,k_r}$. Multizeta values (MZVs) satisfy a number of algebraic relations over ${\mathbb {Q}}$ which we discuss further below. Let us first introduce the monomial notation

2.2 $$\begin{aligned} \zeta (x^{k_r-1}y\cdots x^{k_2-1}y x^{k_1-1}y)=\zeta _{k_1,k_2,\ldots ,k_r} , \end{aligned}$$

where *x* and *y* are non-commutative indeterminates and the convergence property $k_r>1$ implies that the first letter on the left-hand side is *x*. We say that a non-trivial monomial in *x*, *y* is *convergent* if it begins with *x* and ends with *y*; all other monomials are *non-convergent*. We extend the notation (2.2) to the definition of the *regularized zeta values* $\zeta (w)$ for all non-convergent monomials $w=y^r v x^s$ with *v* convergent, by the explicit formula (established in Prop. 3.2.3 of [55], based on the regularization methods of [23])2.3an expression in which all the non-convergent $\zeta (w)$ cancel out so that $\zeta (y^rvx^s)$ is expressed as a linear combination of convergent words only, and which ensures that for all pairs of (convergent or non-convergent) words *u*, *v*, the $\zeta$-values satisfy the shuffle relation2.4where $\zeta$ is considered as a linear function on words, and we fix the values $\zeta (x)=\zeta (y)=0$ and also $\zeta (\textbf{1})=1$, where $\textbf{1}$ in the argument denotes the empty word. We recall here that the shuffle product of monomials can be defined recursively as follows: for any monomial *u*, we have , and if $u,v\ne \textbf{1}$ we write $u=au'$ and $v=bv'$, where *a* and *b* are single letters (either *x* or *y*), and we have2.5For example, writing $\zeta _2=\zeta (xy)$, we have2.6This multiplication rule is called the *shuffle multiplication* of real MZVs.

There is a second multiplication, restricted to a subset of words *w*, which arises when considering the MZVs written as infinite sums as in (2.1). Indeed, the result of multiplying two such series is itself a sum of such series, as can be seen on the first example:

2.7 $$\begin{aligned} \zeta _2^2&=\sum _{n_1\ge 1} n_1^{-2}\sum _{n_2\ge 1} n_2^{-2}\nonumber \\&= \sum _{n_1>n_2\ge 1}n_1^{-2}n_2^{-2}+ \sum _{n_2>n_1\ge 1}n_1^{-2}n_2^{-2}+\sum _{n_1=n_2\ge 1}n_1^{-4}\nonumber \\&= 2\zeta _{2,2}+\zeta _4 . \end{aligned}$$

This product, called the *stuffle product*, can be defined for any pair of words *u*, *v* ending in *y* as follows: we first note that every monomial *u* ending in *y* can be rewritten in the free variables $y_i=x^{i-1}y$, with $i\ge 1$:

2.8 $$\begin{aligned} u=y_{i_1}\cdots y_{i_r} . \end{aligned}$$

We stipulate that for all such monomials, we have $u*\textbf{1}=\textbf{1}*u=u$. Then, in the case where $u,v\ne \textbf{1}$, we peel off the first letter of each of the two words, writing $u=y_{i_1}u'$ and $v=y_{j_1}v'$ with $u'=y_{i_2}\cdots y_{i_r}$ and $v'=y_{j_2}\cdots y_{j_r}$, and define the stuffle product by the recursive rule (first developed by Hoffman in [56])

2.9 $$\begin{aligned} u*v=y_{i_1}(u'*v)+y_{j_1}(u*v')+y_{i_1+j_1}(u'*v') . \end{aligned}$$

The stuffle product is commutative and associative on words ending in *y*.

Associated with the stuffle product, one can define a *stuffle regularization* $\zeta _*(w)$ of MZVs for words ending in *y*. For convergent words *w* (beginning with *x* and ending in *y*) we set $\zeta _*(w) = \zeta (w)$. The stuffle-regularized MZVs for non-convergent words ending in *y* are defined as follows. First we deal with $\zeta _*(y^i)$ for $i\ge 0$ by writing the series

2.10 $$\begin{aligned} \sum _{n\ge 0}\zeta _*(y^n) y^n :=\textrm{exp}\bigg (\sum _{n\ge 2} \frac{(-1)^{n-1}}{n}\zeta (x^{n-1}y)y^n\bigg ) , \end{aligned}$$

leading for instance to

2.11 $$\begin{aligned} \zeta _*(\textbf{1})&=1 , \nonumber \\ \zeta _*(y)&=0 , \nonumber \\ \zeta _*(y^2)&= - \tfrac{1}{2} \zeta (xy) = - \tfrac{1}{2} \zeta _2 , \nonumber \\ \zeta _*(y^3)&=\tfrac{1}{3} \zeta (x^2y)= \tfrac{1}{3}\zeta _3 , \nonumber \\ \zeta _*(y^4)&=-\tfrac{1}{4}\zeta (x^3y)+\tfrac{1}{8}\zeta (xy)^2=-\tfrac{1}{4}\zeta _4+\tfrac{1}{8}\zeta _2^2 . \end{aligned}$$

Then for monomials $y^iv$ for a non-trivial convergent word *v* we define the stuffle regularization by

2.12 $$\begin{aligned} \zeta _*(y^iv)=\sum _{j=0}^i \zeta _*(y^j)\zeta (y^{i-j}v), \end{aligned}$$

where the notation $\zeta (y^{i-j}v)$ refers to the shuffle regularization defined in (2.3).

The stuffle-regularized zeta values $\zeta _*(u)$ defined in this way satisfy the stuffle relations

2.13 $$\begin{aligned} \zeta _*(u)\zeta _*(v)=\zeta _*(u*v)=\zeta _*(v*u) \end{aligned}$$

for every pair of monomials *u*, *v* both ending in *y* as a direct consequence of their infinite sum expressions (2.1) (see the original reference [56]). In particular the stuffle relations hold for ordinary MZVs $\zeta (u)$ and $\zeta (v)$ when *u* and *v* are convergent words; for example, we have

2.14 $$\begin{aligned} xy*xy=y_2*y_2=2y_2^2+y_4=2xyxy+xxxy , \end{aligned}$$

which corresponds to $\zeta _2^2=2\zeta _{2,2}+\zeta _4$ as in (2.7) above. Note that if both *u* and *v* are convergent, then since $\zeta _*(u)=\zeta (u)$ and $\zeta _*(v)=\zeta (v)$, combining (2.4) and (2.13) implies that2.15The family of relations between MZVs consisting of the (“regularized”) shuffle relations (2.4) for all pairs of monomials *u*, *v* and the (“regularized”) stuffle relations (2.13) for all pairs of words *u*, *v* both ending in *y* is known as *the family of regularized double shuffle relations* on MZVs. These were studied fully in [54]; for a standard reference text containing all the basic material on MZVs, see also [52].

#### Formal MZVs

The formal MZVs, denoted by $\zeta ^{\mathfrak {f}}(w)$, are symbols which by definition satisfy only the (regularized) double shuffle relations explained above, as opposed to the real MZVs which may in theory satisfy any number of additional relations, even including the possibility of being rational numbers. General references for this material are [24, 54, 57–59]. Let us introduce the notation for the ring of formal MZVs.

For each $n\ge 0$, let ${\mathbb {Q}}_n[Z(w)]$ denote the vector space spanned by formal symbols *Z*(*w*) indexed by all degree *n* monomials *w* in two non-commutative variables *x* and *y*; in particular we have ${\mathbb {Q}}_0[Z(w)]={\mathbb {Q}}$. We set

2.16 $$\begin{aligned} {\mathbb {Q}}[Z(w)]:=\bigoplus _{n\ge 0} {\mathbb {Q}}_n[Z(w)] , \end{aligned}$$

and make this vector space into a commutative ${\mathbb {Q}}$-algebra by equipping it with the (commutative) shuffle multiplication2.17Let us introduce a second set of formal symbols $Z_*(w)$ for monomials *w* ending in *y*, by

- setting $Z_*(w):=Z(w)$ for convergent *w*,
- defining $Z_*(y^n)$ for $n\ge 1$ by the equation (2.10) with $\zeta$ replaced by *Z*,
- defining $Z_*(y^iv)$ for convergent words *v* by equation (2.12) with $\zeta$ replaced by *Z*.

Given that multiplying the symbols *Z*(*w*) by the shuffle multiplication (2.17) reduces products to linear combinations, all of the new symbols $Z_*(w)$ can be expressed in terms of linear combinations of the symbols *Z*(*w*).

##### Definition 2.1.1

Let ${\mathcal {I}}_{\mathcal{F}\mathcal{Z}}$ be the ideal of the ring ${\mathbb {Q}}[Z(w)]$ generated by the following two families of linear combinations: on the one hand the *regularizations*2.18for all words $w =y^r v x^s$ with *v* convergent (adapted from (2.3)), and on the other hand the regularized stuffles given for all pairs of monomials *u* and *v* both ending in *y* by

2.19 $$\begin{aligned} Z_*(u)Z_*(v)&-Z_*(u*v) \end{aligned}$$

(adapted from (2.13)). The expression (2.19) is to be computed as a linear combination of symbols $Z(w')$ where the monomials $w'$ are all of homogeneous weight equal to the sum of the weights of *u* and *v* by (i) expanding out the right-hand term as a linear combination, (ii) replacing every occurrence of $Z_*$ by a polynomial expression in *Z* using (2.10) and (2.12), (iii) using the shuffle multiplication (2.17) to express all products $Z(w')Z(w'')$ as linear combinations . Thus each of the expressions in (2.18) and (2.19) is a linear combination of fixed weight; we take them all together as the generators of the ideal $\mathcal {I}_{{\mathcal{F}\mathcal{Z}}}$.

##### Examples

*Regularization:* the formula (2.18) above for the non-convergent word $w=yxy$ tells us to add the linear combination2.20to the ideal ${\mathcal {I}}_{\mathcal{F}\mathcal{Z}}$.

*Stuffle:* Let us compute the linear combination

2.21 $$\begin{aligned} Z_*(y^2)Z_*(xy)-Z_*(y^2*xy) \end{aligned}$$

as a linear combination of *Z*-symbols using the three steps explained below (2.19). Using (2.9), we have

2.22 $$\begin{aligned} yy*xy&=y_1y_1*y_2=y_2y_1y_1+y_1y_2y_1+y_1y_2y_2+y_3y_1+y_1y_3\nonumber \\&=xyyy+yxyy+yyxy+xxyy+yxxy , \end{aligned}$$

so by the first step, which consists of expanding out $Z_*(yy*xy)$, (2.21) can be rewritten as

2.23 $$\begin{aligned} Z_*(yy)Z_*(xy)-Z_*(xyyy)-Z_*(yxyy)-Z_*(yyxy)-Z_*(xxyy)-Z_*(yxxy). \end{aligned}$$

In the second step we replace each $Z_*$ by an expression in *Z*. For the three convergent words *xy*, *xyyy* and *xxyy* we have $Z_*=Z$; by (2.11) we have $Z_*(y)=0$ and $Z_*(yy)=-\tfrac{1}{2}Z(xy)$, and finally by (2.12) we have

2.24 $$\begin{aligned} Z_*(yxyy)&=Z(yxyy)+Z_*(y)Z(xyy)=Z(yxyy) , \nonumber \\ Z_*(yyxy)&=Z(yyxy)+Z_*(y)Z(yxy)+Z_*(yy)Z(xy)=Z(yyxy)-\tfrac{1}{2}Z(xy)^2 ,\nonumber \\ Z_*(yxxy)&=Z(yxxy)+Z_*(y)Z(xxy)=Z(yxxy) . \end{aligned}$$

Plugging these into (2.23) allows us to rewrite (2.21) as

2.25 $$\begin{aligned} -\tfrac{1}{2}Z(xy)^2-Z(xyyy)-Z(yxyy)-Z(yyxy)+\tfrac{1}{2}Z(xy)^2-Z(xxyy)-Z(yxxy). \end{aligned}$$

If necessary we could now expand out the products of *Z*-symbols using the shuffle, but since they cancel out we don’t need to, so in the end we add the linear combination

2.26 $$\begin{aligned} -Z(xyyy)-Z(yxyy)-Z(yyxy)-Z(xxyy)-Z(yxxy) \end{aligned}$$

to the ideal ${\mathcal {I}}_{{\mathcal{F}\mathcal{Z}}}$.

##### Remark 2.1.2

Note that by (2.17), for convergent words *u* and *v*, the relations (2.19) of $\mathcal {I}_{{\mathcal{F}\mathcal{Z}}}$ are of the “shuffle=stuffle” form  since $Z_*(u)=Z(u)$ and $Z_*(v)=Z(v)$. A conjecture by Hoffman (cf. [60] which is useful for practical computations in low weight and further discussed in [54]) posits that the combinations2.27with both *u* and *v* convergent or $u=y$ and *v* convergent suffice to generate the ideal $\mathcal {I}_{{\mathcal{F}\mathcal{Z}}}$.

##### Definition 2.1.3

Let ${\mathcal {I}}_{\mathcal {Z}}$ be the ideal of ${\mathbb {Q}}[Z(w)]$ generated by all algebraic relations between real MZVs, as defined in (2.1). Since these are known to satisfy the regularized double shuffle relations, we have the inclusions

2.28 $$\begin{aligned} {\mathcal {I}}_{\mathcal{F}\mathcal{Z}}\subset {\mathcal {I}}_{\mathcal {Z}}\subset {\mathbb {Q}}[Z(w)] . \end{aligned}$$

The space ${\mathcal{F}\mathcal{Z}}$ of *formal MZVs* and the space ${\mathcal {Z}}$ of *real MZVs* are defined by

2.29 $$\begin{aligned} {\mathcal{F}\mathcal{Z}}&:={\mathbb {Q}}[Z(w)]/{\mathcal {I}}_{\mathcal{F}\mathcal{Z}},\nonumber \\ {\mathcal {Z}}&:={\mathbb {Q}}[Z(w)]/{\mathcal {I}}_{\mathcal {Z}}, \end{aligned}$$

so that there is a natural surjection

2.30 $$\begin{aligned} {\mathcal{F}\mathcal{Z}}\rightarrow \!\!\!\!\!\rightarrow {\mathcal {Z}}. \end{aligned}$$

The space ${\mathcal{F}\mathcal{Z}}$ is generated by the images of the *Z*(*w*) in the quotient modulo ${\mathcal {I}}_{\mathcal{F}\mathcal{Z}}$, which we denote $\zeta ^{\mathfrak {f}}(w)$; these formal MZVs are subject by definition only to the regularized double shuffle relations coming from Definition 2.1.1. The elements of the ${\mathbb {Q}}$-algebra ${\mathcal {Z}}$ of real MZVs are denoted by $\zeta (w)$.

The ${\mathbb {Q}}$-algebra ${\mathcal{F}\mathcal{Z}}$ is weight-graded by definition since all of its defining relations are weight-graded, while ${\mathcal {Z}}$ is conjectured but of course not known to be weight-graded; if it were, this would imply that all real MZVs are transcendental since any MZV which is algebraic would have to be the root of a ${\mathbb {Q}}$ polynomial in which each term would be of different weight. A standard conjecture asserts that the surjection (2.30) is an isomorphism.

#### The Goncharov–Brown coaction

Let ${\overline{{\mathcal{F}\mathcal{Z}}}}$ denote the quotient of ${\mathcal{F}\mathcal{Z}}$ modulo the ideal generated by $\zeta ^{\mathfrak {f}}_2$. In [1, 34], Goncharov introduced a coproduct on the space of motivic iterated integrals, making it into a Hopf algebra.

In his thesis, published as [57], Racinet showed that the space $\mathfrak {ds}$ of double-shuffle Lie polynomials is a Lie algebra under the Ihara bracket to be defined below in (3.14), which implies that the universal enveloping algebra ${\mathcal {U}}{\mathfrak {ds}}$ is a Hopf algebra. In [1], Goncharov showed that his coproduct is dual to the (Poincaré–Birkhoff–Witt) multiplication on ${\mathcal {U}}{\mathfrak {ds}}$. Therefore, ${\overline{{\mathcal{F}\mathcal{Z}}}}$, which is the dual of ${\mathcal {U}}{\mathfrak {ds}}$, is a Hopf algebra under the Goncharov coproduct. These objects and ideas are made fully explicit in the discussion around (3.16). Brown’s work [2] allows us to define an extension of Goncharov’s coproduct on the Hopf algebra ${{\overline{{\mathcal{F}\mathcal{Z}}}}}$ to a coaction on the comodule

2.31 $$\begin{aligned} {\mathcal{F}\mathcal{Z}}\cong {\mathbb {Q}}[\zeta ^{\mathfrak {f}}_2]\otimes _{\mathbb {Q}}{\overline{{\mathcal{F}\mathcal{Z}}}} \end{aligned}$$

as in (2.46) below. See section 5 of [58] for an introductory recapitulation of these facts.

There are in fact two different versions of the Goncharov–Brown coaction, which differ from each other only by the order of the tensor factors. We denote them by

2.32 $$\begin{aligned} {\left\{ \begin{array}{ll} \Delta ^{GB} : {\mathcal{F}\mathcal{Z}}\rightarrow {\overline{{\mathcal{F}\mathcal{Z}}}}\otimes {\mathcal{F}\mathcal{Z}},\\ \Delta _{GB} : {\mathcal{F}\mathcal{Z}}\rightarrow {\mathcal{F}\mathcal{Z}}\otimes {\overline{{\mathcal{F}\mathcal{Z}}}} . \end{array}\right. } \end{aligned}$$

Both versions of the coaction are used regularly in the literature, so that it is important to keep track of which one is being used at all times. In the present paper, as we will specify, the coaction $\Delta ^{GB}$ is implicitly used in numerous proofs in view of its compatibility with double-shuffle theory and Hopf-algebra duals. The coaction $\Delta _{GB}$ entering explicit formulae (most notably in Sect. 4) is used to remain coherent with the recent literature.^4^

Let us describe the construction of the Goncharov–Brown coaction $\Delta _{GB}$.

##### Definition 2.1.4

Let *w* be a convergent monomial in *x* and *y*, i.e. starting with *x* and ending with *y*. Write $w=x^{k_r-1}y\cdots x^{k_1-1}y$ to match the monomial notation of $\zeta ^{\mathfrak {f}}_{k_1,\ldots ,k_r}$ in (2.2), and associate to it the symbol

2.33 $$\begin{aligned} I(0;1,0^{k_1-1},\ldots ,1, 0^{k_r-1};1) = \zeta ^{\mathfrak {f}}_{k_1,\ldots ,k_r} . \end{aligned}$$

**Fig. 1:**
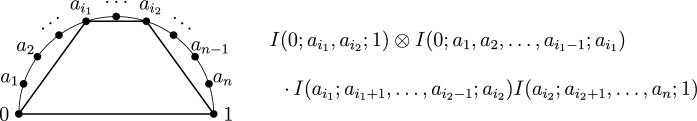
Contributions to the coaction formula (2.35) for $\Delta _{GB} I(0;a_{1},a_{2},\ldots ,a_n;1)$ from polygons with inner vertices $a_{i_1},a_{i_2}$, i.e. quadrilaterals associated with subsets of $a_1,a_2,\ldots ,a_n$ of cardinality $r=2$

Let $n=k_1+\cdots +k_r$ denote the degree of *w*. Visualize the sequence $(0;1,0^{k_1-1},\ldots ,1,0^{k_r-1};1)$ in order from left to right around a semi-circle as illustrated in Fig. 1, with the terminal 0 and 1 at the outer edges and the middle *n* points placed in clockwise order along the inner part of the semi-circle. To compute the coaction of the symbol $I(0;1,0^{k_1-1},\ldots ,1,0^{k_r-1};1)$ associated with $\zeta ^{\mathfrak {f}}_{k_1,\ldots ,k_r}$, draw every possible “polygon” inside the half-circle starting with the outer 0 on the left and ending with the outer 1 on the right, with vertices at any subset of the inner letters (including the empty set). In the notation

2.34 $$\begin{aligned} (a_1,a_2,\ldots ,a_n)=(1,0^{k_1-1},1,0^{k_2-1},\ldots ,1,0^{k_r-1}) \end{aligned}$$

for the middle *n* points (apart from the outer points 0 and 1), the contributing polygons are parametrized by subsets $\{a_{i_1},a_{i_2},\ldots ,a_{i_r}\}$ with $1\le i_1<i_2< \cdots <i_r\le n$ and all cardinalities in the range $0\le r \le n$; see Fig. 1 for the example of $r=2$.

The coaction is computed by adding up the contributions of all possible polygons:

2.35 $$\begin{aligned}&\Delta _{GB} I(0;a_{1},a_{2},\ldots ,a_{n};1) \nonumber \\&\quad = \sum _{r=0}^n \sum _{1\le i_1< i_2< \ldots < i_r \le n} \! \! \! \! \! \! \! \! \! \! \! I(0;a_{i_1},a_{i_2},\ldots ,a_{i_r};1)\otimes I(0;a_{1},a_2,\ldots , a_{{i_1}-1};a_{i_1}) \nonumber \\&\quad \!\!\cdot I(a_{i_1};a_{{i_1}+1},\ldots , a_{{i_2}-1};a_{i_2}) \cdots I(a_{i_{r-1}};a_{{i_{r-1}}+1},\ldots , a_{{i_r}-1};a_{i_r}) I(a_{i_r}; a_{{i_r}+1},\ldots ,a_n;1) , \end{aligned}$$

where $I(0;a_{i_1},\ldots ,a_{i_r};1)$ specializes to $I(0;1)=1$ in case of the empty subset at $r=0$ and *I*(0; *A*; 1)*I*(0; *B*; 1) (or $I(0; A;1) \cdot I(0; B;1)$) is the shuffle product. We simplify the expression (2.35) according to the following rules:

- $$I(a;b)=1$$ for all $$a,b\in \{0,1\}$$,
- $$I(a;b;c)=0$$ for all $$a,b,c\in \{0,1\}$$,
- $I(a;S;a)=0$ for $a\in \{0,1\}$ and any non-empty sequence *S* of $0's$ and 1’s,
- $I(1;S;0)=(-1)^nI(0;\overleftarrow{S};1)$ if *S* is a sequence of 0’s and 1’s of length *n* and $\overleftarrow{S}$ denotes the sequence *S* in the reversed order.

We can also replace each term *I*(0; *S*; 1) by the formal (shuffle-regularized) MZV $\zeta ^{\mathfrak {f}}(w_S)$, where if *S* is any sequence of 0’s and 1’s then $w_S$ is the monomial obtained by reversing the order of *S* and replacing every 0 with an *x* and every 1 with a *y*. We finally project the entries of the second factor of the tensor product modulo $\zeta ^{\mathfrak {f}}(xy)=\zeta ^{\mathfrak {f}}_2$ to ${\overline{{\mathcal{F}\mathcal{Z}}}}$, so that the Goncharov–Brown coaction takes values in ${\mathcal{F}\mathcal{Z}}\otimes {\overline{{\mathcal{F}\mathcal{Z}}}}$ as announced in (2.32).

##### Example

**Fig. 2:**
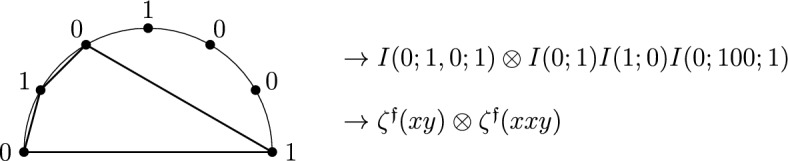
Example of a contribution to $\Delta _{GB} \zeta ^{\mathfrak {f}}(xxyxy)$ as computed in (2.36)

The coaction on the convergent word $\zeta ^{\mathfrak {f}}(xxyxy)$ is computed from the semi-circle drawn in Fig. 2, which shows one example of a contribution from a quadrilateral. The total result of the coaction is given by

2.36 $$\begin{aligned} \Delta _{GB} \zeta ^{\mathfrak {f}}(xxyxy) = 1 \otimes \zeta ^{\mathfrak {f}}(xxyxy) + \zeta ^{\mathfrak {f}}(xxyxy) \otimes 1 +3 \zeta ^{\mathfrak {f}}(xy) \otimes \zeta ^{\mathfrak {f}}(xxy) . \end{aligned}$$

The first term comes from the degenerate polygon consisting of the straight line from the outer 0 to the outer 1 with no inner vertices and the second to the full polygon touching all the inner vertices. The term with factor 3 arises from quadrilaterals involving the earliest 1 (in clockwise direction) of the type shown in Fig. 2, and there are three such quadrilaterals which produce the same non-vanishing contribution. All other polygons have a vanishing contribution; in particular the polygons going from 0 directly to the 1 at the top produce a $\zeta ^{\mathfrak {f}}(xy)=\zeta ^{\mathfrak {f}}_2$ to the right of the tensor product $\otimes$, which is projected to zero.

##### Definition 2.1.5

The coaction $\Delta ^{GB}$ is obtained from $\Delta _{GB}$ by the identity

2.37 $$\begin{aligned} \Delta ^{GB}=\iota \circ \Delta _{GB} , \end{aligned}$$

where $\iota$ exchanges the two tensor factors

2.38 $$\begin{aligned} \iota :{\mathcal{F}\mathcal{Z}}\otimes {\overline{{\mathcal{F}\mathcal{Z}}}}&\mapsto {\overline{{\mathcal{F}\mathcal{Z}}}}\otimes {\mathcal{F}\mathcal{Z}},\nonumber \\ \alpha \otimes \beta&\mapsto \beta \otimes \alpha . \end{aligned}$$

Reducing the ${\mathcal{F}\mathcal{Z}}$ factor mod $\zeta ^{\mathfrak {f}}_2$ in not just one but both factors of the image yields two coproducts

2.39 $$\begin{aligned} \Delta _G,\Delta ^G:{\overline{{\mathcal{F}\mathcal{Z}}}}\rightarrow {\overline{{\mathcal{F}\mathcal{Z}}}} , \end{aligned}$$

each of which confers a Hopf algebra structure on ${\overline{{\mathcal{F}\mathcal{Z}}}}$. We will study the Hopf algebra ${\overline{{\mathcal{F}\mathcal{Z}}}}$ equipped with $\Delta ^G$ and its dual Hopf algebra ${\overline{{\mathcal{F}\mathcal{Z}}}}^\vee$ further in Sect. 3.1.

### Motivic MZVs

We shall here recall the ${\mathbb {Q}}$-algebra of *motivic MZVs*, which were constructed and studied in depth as a subcategory of the category of mixed Tate motives (*MTM*) unramified over $\mathbb {Z}$ by Deligne, Goncharov, Manin and others, until Brown proved that the subcategory is equal to the full category (see [2]). Our definition follows from Brown’s results. For further reading, see [52, 67, 68]. The motivic Lie algebra associated to motivic MZVs is known to be freely generated by one element in each odd degree; a fact that we will make crucial use of in this paper.

#### Motivic versus formal multizetas, coproduct and coaction

Let ${\overline{{\mathcal{M}\mathcal{Z}}}}$ denote the space of motivic multizetas (modulo $\zeta ^\mathfrak {m}_2$) as defined by Goncharov [1]. It is known to be a Hopf algebra equipped with the coproduct that we introduced in Sect. 2.1.3.

Goncharov showed that these motivic MZVs satisfy the double shuffle relations and surject via the period map to the quotient ${{\overline{{\mathcal {Z}}}}}:={\mathcal {Z}}/\langle \zeta _2\rangle$ of the $\mathbb Q$-algebra of real multizetas by the ideal generated by $\zeta _2$. We therefore have the surjections

2.40 $$\begin{aligned} {\overline{{\mathcal{F}\mathcal{Z}}}}\rightarrow \!\!\!\!\!\rightarrow {\overline{{\mathcal{M}\mathcal{Z}}}} \rightarrow \!\!\!\!\!\rightarrow {\overline{{\mathcal {Z}}}} . \end{aligned}$$

Let ${\mathcal{M}\mathcal{Z}}$ be the Hopf-algebra comodule of motivic multizeta values defined by Brown [2]; he showed that it has the structure

2.41 $$\begin{aligned} {\mathcal{M}\mathcal{Z}}\cong {\mathbb {Q}}[\zeta ^{\mathfrak {m}}_2]\otimes _{\mathbb {Q}}{\overline{{\mathcal{M}\mathcal{Z}}}} . \end{aligned}$$

This allows us to lift the first surjection in (2.40) to a surjection from the space of formal multizetas $\mathcal{F}\mathcal{Z}$ thanks to (2.31) to the space of motivic multizetas $\mathcal{M}\mathcal{Z}$ as follows: for every word *w* in *x*, *y* of length $>2$, we map $\zeta ^{\mathfrak {f}}(w) \in {\overline{{\mathcal{F}\mathcal{Z}}}}$ to $\zeta ^{\mathfrak {m}}(w) \in {\overline{{\mathcal{M}\mathcal{Z}}}}$ and similarly map $\zeta ^{\mathfrak {f}}_2=\zeta ^{\mathfrak {f}}(xy)$ to $\zeta _2^{\mathfrak {m}}=\zeta ^{\mathfrak {m}}(xy)$.

For any sequence *S* of letters 0, 1, let $I^{\mathfrak {m}}(0;S;1)$ denote the image of the $I(0;S;1) \in {\mathcal{F}\mathcal{Z}}$ in Sect. 2.1.3. The motivic MZVs surject down to the real MZVs by the period map

2.42 $$\begin{aligned} I^{\mathfrak {m}}(0;S;1)\mapsto \zeta (w_S) \end{aligned}$$

(see [2]) with $w_S$ as in Sect. 2.1.3, so (2.40) lifts to the following sequence of ${\mathbb {Q}}$-algebra surjections

2.43 $$\begin{aligned} {\mathcal{F}\mathcal{Z}}\rightarrow \!\!\!\!\!\rightarrow {\mathcal{M}\mathcal{Z}}\rightarrow \!\!\!\!\!\rightarrow {\mathcal {Z}}, \end{aligned}$$

with conjectured equality.

Like ${\mathcal{F}\mathcal{Z}}$, the Hopf algebra comodule ${\mathcal{M}\mathcal{Z}}$ is graded by the weight of the MZVs, as is ${\overline{{\mathcal{M}\mathcal{Z}}}}$. We write ${\mathcal{M}\mathcal{Z}}_w$ (resp. ${\overline{{\mathcal{M}\mathcal{Z}}}}_w$  ${\mathcal{F}\mathcal{Z}}_w$, ${\overline{{\mathcal{F}\mathcal{Z}}}}_w$) for the weight *w* part of ${\mathcal{M}\mathcal{Z}}$ (resp. ${\overline{{\mathcal{M}\mathcal{Z}}}}$  ${\mathcal{F}\mathcal{Z}}$, ${\overline{{\mathcal{F}\mathcal{Z}}}}$). Note that we have

2.44 $$\begin{aligned} {\mathcal{F}\mathcal{Z}}_0&={\overline{{\mathcal{F}\mathcal{Z}}}}_0={\mathcal{M}\mathcal{Z}}_0=\overline{{\mathcal{M}\mathcal{Z}}_0}={\mathbb {Q}},\nonumber \\ {\mathcal{F}\mathcal{Z}}_1&={\overline{{\mathcal{F}\mathcal{Z}}}}_1={\mathcal{M}\mathcal{Z}}_1=\overline{{\mathcal{M}\mathcal{Z}}_1}=\{0\} . \end{aligned}$$

The coactions $\Delta ^{GB}$ and $\Delta _{GB}$ reviewed in Sect. 2.1.3 both descend directly to ${\mathcal{M}\mathcal{Z}}$. Let us recall the notation for $\Delta _{GB}$; it is identical to $\Delta ^{GB}$ in (2.32) up to exchanging the two factors of the tensor product. The descended coaction [2]

2.45 $$\begin{aligned} \Delta _{GB} : {\mathcal{M}\mathcal{Z}}\rightarrow {\mathcal{M}\mathcal{Z}}\otimes {\overline{{\mathcal{M}\mathcal{Z}}}} , \end{aligned}$$

makes ${\mathcal{M}\mathcal{Z}}$ into a Hopf algebra comodule. In particular we have

2.46 $$\begin{aligned} \Delta _{GB}\bigl (\zeta ^{\mathfrak {m}}_2\bigr )=\zeta ^{\mathfrak {m}}_2\otimes 1 . \end{aligned}$$

In analogy with (2.33) we write $\zeta ^{\mathfrak {m}}_{k_1,\ldots ,k_r}=I^{\mathfrak {m}}(0;1,0^{k_1-1},\ldots ,1, 0^{k_r-1};1)\in {\mathcal{M}\mathcal{Z}}$. We also use the notation $\zeta ^{\mathfrak {dr}}_{k_1,\ldots ,k_r}=I^{\mathfrak {dr}}(0;1,0^{k_1-1},\ldots ,1, 0^{k_r-1};1)\in {\overline{{\mathcal{M}\mathcal{Z}}}}$ for the second tensor factor of $\Delta _{GB}$ whose reduction modulo $\zeta _2$ translates into $\zeta ^{\mathfrak {dr}}_2=0$.^5^ The explicit form of the coaction for motivic MZVs $\zeta ^{\mathfrak {m}}_{k_1,\ldots ,k_r}$ is encoded in symbols exactly as in (2.35): we write

2.47 $$\begin{aligned}&\Delta _{GB} I^{\mathfrak {m}}(0;a_{1},a_{2},\ldots ,a_{n};1)= \sum _{r=0}^n \sum _{1\le i_1< i_2< \ldots < i_r \le n} \! \! \! \! \! \! I^{\mathfrak {m}}(0;a_{i_1},\ldots ,a_{i_r};1)\otimes I^{\mathfrak {dr}}(0;a_{1},\ldots , a_{{i_1}-1};a_{i_1}) \nonumber \\&\quad \cdot I^{\mathfrak {dr}}(a_{i_1};a_{{i_1}+1},\ldots , a_{{i_2}-1};a_{i_2}) \cdots \nonumber \\&\quad \cdot I^{\mathfrak {dr}}(a_{i_{r-1}};a_{{i_{r-1}}+1},\ldots , a_{{i_r}-1};a_{i_r}) I^{\mathfrak {dr}}(a_{i_r}; a_{{i_r}+1},\ldots ,a_n;1) , \end{aligned}$$

where the rules detailed below (2.35) apply in identical form to the terms $I^{\mathfrak {m}}$ and $I^{\mathfrak {dr}}$ on the right-hand side of (2.47) and can be used to put all terms into the standard form $I^{\mathfrak {m}}(0;S;1)$ and $I^{\mathfrak {dr}}(0;S;1)$ for finite tuples *S* of 0’s and 1’s.

##### Examples

When $w=x^{n-1}y$ for odd values of $n=2k+1$, the only polygons with a non-zero contribution are the degenerate one (going directly from 0 to 1) and the full polygon including every point on the semi-circle: thus we have

2.48 $$\begin{aligned} \Delta _{GB}\zeta ^{\mathfrak {m}}_{2k+1}&= \zeta ^{\mathfrak {m}}_{2k+1} \otimes 1 + 1\otimes \zeta ^{\mathfrak {dr}}_{2k+1}\in {{\mathcal{M}\mathcal{Z}}}\otimes {\overline{{\mathcal{M}\mathcal{Z}}}} . \end{aligned}$$

Such elements are said to be *primitive* for the coproduct. The counterparts of (2.48) for $w=x^{n-1}y$ at even $n=2k$ simplifies to $\Delta _{GB} \zeta ^{\mathfrak {m}}_{2k} = \zeta ^{\mathfrak {m}}_{2k}\otimes 1$ by the vanishing of $\zeta ^{\mathfrak {dr}}_{2k}$.

We also give a few other illustrative instances:

2.49 $$\begin{aligned} \Delta _{GB}(\zeta ^{\mathfrak {m}}_3 \zeta ^{\mathfrak {m}}_5)&= \zeta ^{\mathfrak {m}}_3 \zeta ^{\mathfrak {m}}_5 \otimes 1 + 1\otimes \zeta ^{\mathfrak {dr}}_3 \zeta ^{\mathfrak {dr}}_5 + \zeta ^{\mathfrak {m}}_3\otimes \zeta ^{\mathfrak {dr}}_5 + \zeta ^{\mathfrak {m}}_5 \otimes \zeta ^{\mathfrak {dr}}_3 ,\nonumber \\ \Delta _{GB}(\zeta ^{\mathfrak {m}}_{3,5})&= \zeta ^{\mathfrak {m}}_{3,5} \otimes 1 + 1\otimes \zeta ^{\mathfrak {dr}}_{3,5} -5\, \zeta ^{\mathfrak {m}}_3 \otimes \zeta ^{\mathfrak {dr}}_5 ,\nonumber \\ \Delta _{GB}(\zeta ^{\mathfrak {m}}_{2,6})&= \zeta ^{\mathfrak {m}}_{2,6} \otimes 1 + 1\otimes \zeta ^{\mathfrak {dr}}_{2,6} +4\, \zeta ^{\mathfrak {m}}_3 \otimes \zeta ^{\mathfrak {dr}}_5+2\, \zeta ^{\mathfrak {m}}_5 \otimes \zeta ^{\mathfrak {dr}}_3 . \end{aligned}$$

These relations are compatible with

2.50 $$\begin{aligned} \zeta ^{\mathfrak {m}}_{2,6} = - \frac{2}{5} \zeta ^{\mathfrak {m}}_{3,5} + 2\zeta ^{\mathfrak {m}}_3 \zeta ^{\mathfrak {m}}_5 - \frac{42}{125} (\zeta ^{\mathfrak {m}}_2)^4 , \end{aligned}$$

where one has to use that the second entries of tensor products in ${\mathcal{M}\mathcal{Z}}\otimes {\overline{{\mathcal{M}\mathcal{Z}}}}$ are automatically projected modulo $\zeta ^{\mathfrak {m}}_2$, so that $\zeta ^{\mathfrak {dr}}_{2,6} = -\frac{2}{5} \zeta ^{\mathfrak {dr}}_{3,5} + 2 \zeta ^{\mathfrak {dr}}_3\zeta ^{\mathfrak {dr}}_5$.

#### Reducible motivic MZVs

General references for the following subsection are [53, 55]. Let ${\mathfrak {fz}}$ denote the quotient of the ${\mathbb {Q}}$-algebra ${\mathcal{F}\mathcal{Z}}$ given by

2.51 $$\begin{aligned} {\mathfrak {fz}}:={\mathcal{F}\mathcal{Z}}/\bigl ({\mathcal{F}\mathcal{Z}}_0\oplus {\mathcal{F}\mathcal{Z}}_2\oplus ({\mathcal{F}\mathcal{Z}}_{>0})^2\bigr )= {\overline{{\mathcal{F}\mathcal{Z}}}}/\bigl ({\overline{{\mathcal{F}\mathcal{Z}}}}_0\oplus ({\overline{{\mathcal{F}\mathcal{Z}}}}_{>0})^2\bigr ) , \end{aligned}$$

and analogously, let ${\mathfrak {mz}}$ denote the quotient of the ${\mathbb {Q}}$-algebra ${\mathcal{M}\mathcal{Z}}$ given by

2.52 $$\begin{aligned} {\mathfrak {mz}}:={\mathcal{M}\mathcal{Z}}/\bigl ({\mathcal{M}\mathcal{Z}}_0\oplus {\mathcal{M}\mathcal{Z}}_2\oplus ({\mathcal{M}\mathcal{Z}}_{>0})^2\bigr )= {\overline{{\mathcal{M}\mathcal{Z}}}}/\bigl ({\overline{{\mathcal{M}\mathcal{Z}}}}_0\oplus ({\overline{{\mathcal{M}\mathcal{Z}}}}_{>0})^2\bigr ) . \end{aligned}$$

From the Hopf algebra structure on ${\overline{{\mathcal{F}\mathcal{Z}}}}$ (resp. ${\overline{{\mathcal{M}\mathcal{Z}}}}$), the vector space ${\mathfrak {fz}}$ (resp. ${\mathfrak {mz}}$) inherits the structure of a Lie coalgebra, dual to the Lie algebras that will be introduced in Sect. 3.1. Note that by (2.51) and (2.52), the element $\zeta ^{\mathfrak {f}}_2$ (resp. $\zeta ^{\mathfrak {m}}_2$) maps down to zero in ${\mathfrak {fz}}$ (resp. ${\mathfrak {mz}}$).

##### Definition 2.2.1

For all even positive integers $w=2n$, let $\textrm{B}_{2n}$ be the Bernoulli number, and set [2]

2.53 $$\begin{aligned} \zeta ^{\mathfrak {m}}_{2n} :=\frac{\zeta _{2n}}{\zeta _2^{n}}(\zeta ^{\mathfrak {m}}_2)^{n}= (-1)^{n-1} \frac{(24)^n \textrm{B}_{2n}}{2 (2n)!} \, (\zeta ^{\mathfrak {m}}_2)^n\in {\mathcal{M}\mathcal{Z}}_{2n} . \end{aligned}$$

##### Definition 2.2.2

For all $w\ge 3$, let ${\hat{R}}_w$ denote the canonical subspace of *reducible MZVs* in ${\mathcal{M}\mathcal{Z}}_w$. The space ${\hat{R}}_w$ is the subspace generated by all total-weight *w* products of lower-weight MZVs, or in other words by all weight *w* elements of $({{\mathcal{M}\mathcal{Z}}}_{>0})^2$. Note that ${\hat{R}}_3=\{0\}$, so there are actually non-trivial reducible subspaces only for $w\ge 4$, starting with ${\hat{R}}_4={\mathbb {Q}} \zeta ^{\mathfrak {m}}_4$ and ${\hat{R}}_5 = {\mathbb {Q}} \zeta ^{\mathfrak {m}}_2 \zeta ^{\mathfrak {m}}_3$.

The Lie coalgebras ${\mathfrak {fz}}$ and ${\mathfrak {mz}}$ are weight-graded, and for each weight $w>1$ we have

2.54 $$\begin{aligned} {\mathfrak {fz}}_w={\mathcal{F}\mathcal{Z}}_w/\hat{R}_w , \qquad {\mathfrak {mz}}_w={\mathcal{M}\mathcal{Z}}_w/\hat{R}_w . \end{aligned}$$

#### Irreducible MZVs

Let ${\hat{I}}_w$ be any supplementary subspace of ${\hat{R}}_w$ in ${\mathcal{M}\mathcal{Z}}_w$ so that

2.55 $$\begin{aligned} {\mathcal{M}\mathcal{Z}}_w={\hat{R}}_w\oplus {\hat{I}}_w . \end{aligned}$$

Since the map ${\mathcal{M}\mathcal{Z}}_w\rightarrow {\mathfrak {mz}}_w$ is the quotient mod ${\hat{R}}_w$, it induces an isomorphism ${\hat{I}}_w\rightarrow {\mathfrak {mz}}_w$. We will always choose ${\hat{I}}_w$ containing $\zeta ^{\mathfrak {m}}_w$ if *w* is odd, see Lemma 3.2 of [2]. If *w* is even, we set $I_w :={\hat{I}}_w$, and if *w* is odd we choose a supplementary subspace $I_w$ in ${\hat{I}}_w$ such that ${\hat{I}}_w={\mathbb {Q}}\zeta ^{\mathfrak {m}}_w\oplus I_w$. Similarly, if *w* is odd we set $R_w :={\hat{R}}_w$ and if *w* is even we choose a supplementary subspace $R_w\subset {\hat{R}}_w$ such that ${\hat{R}}_w={\mathbb {Q}}\zeta ^{\mathfrak {m}}_w\oplus R_w.$ Then for all $w\ge 2$ we have the direct sum decomposition

2.56 $$\begin{aligned} {\mathcal{M}\mathcal{Z}}_w={\mathbb {Q}}\zeta ^{\mathfrak {m}}_w\oplus I_w\oplus R_w . \end{aligned}$$

## The Z-Map Associating Polynomials to MZVs

In this section we will introduce the *Z-map* (see [20]), which provides a family of canonical isomorphisms between the MZV spaces studied in Sect. 2 (namely ${\mathcal{F}\mathcal{Z}}$, ${\overline{{\mathcal{F}\mathcal{Z}}}}$, ${\mathcal{M}\mathcal{Z}}$, ${\overline{{\mathcal{M}\mathcal{Z}}}}$, ${\mathcal {Z}}$, ${\mathfrak {fz}}$ or ${\mathfrak {mz}}$) and their dual spaces. As will be detailed in Sect. 3.1, since all the MZV spaces are quotients of ${\mathbb {Q}}[Z(w)]$, all of their duals are subspaces of ${\mathbb {Q}}[Z(w)]^\vee$, which is nothing other than the polynomial algebra ${\mathbb {Q}}\langle x,y\rangle$ in the non-commutative variables *x* and *y*.

Thanks to the fact that the double shuffle relations generate all relations satisfied by ${\mathcal{F}\mathcal{Z}}$ (and in their linearized version, ${\mathfrak {fz}}$), we can give an explicit description of the elements of the dual spaces ${\mathcal{F}\mathcal{Z}}^\vee$ and ${\mathfrak {fz}}^\vee$ in ${\mathbb {Q}}\langle x,y\rangle$. In the case of motivic and real MZVs we do not have an explicit description of this type since they may satisfy further, unknown relations. Still, thanks to Brown’s theorem in [2], we do know the structure and dimensions of the graded parts of the dual spaces ${\mathcal{M}\mathcal{Z}}^\vee$ and ${\mathfrak {mz}}^\vee$, which allows us to compute their elements explicitly in low weights (see Sect. 3.4).

### The double shuffle dual space of formal MZVs

Let ${\mathbb {Q}}\langle x,y\rangle$ denote the polynomial ring in two non-commutative variables *x* and *y*, equipped with its canonical basis of monomials *w* in *x* and *y* (including the constant monomial $\textbf{1}$), and let ${\mathbb {Q}}\langle \langle x,y\rangle \rangle$ denote its degree-completion, the power series ring in *x* and *y*. The space ${\mathbb {Q}}[Z(w)]$ introduced in Sect. 2.1.2 can be identified with the graded dual of ${\mathbb {Q}}\langle x,y\rangle$, equipped with the dual basis of symbols *Z*(*w*) such that

3.1 $$\begin{aligned} \langle Z(u),v\rangle =\delta _{u,v} , \end{aligned}$$

on monomials *u* and *v* and extended linearly to give a canonical pairing between ${\mathbb {Q}}\langle x,y\rangle$ and ${\mathbb {Q}}[Z(w)]$.

Recall from (2.29) that ${\mathcal{F}\mathcal{Z}}$ is the quotient of ${\mathbb {Q}}[Z(w)]$ by the ideal ${\mathcal {I}}_{{\mathcal{F}\mathcal{Z}}}$. The graded dual space ${\mathcal{F}\mathcal{Z}}^\vee$ is thus the subspace of ${\mathbb {Q}}\langle x,y\rangle$ that annihilates the elements of ${\mathcal {I}}_{\mathcal{F}\mathcal{Z}}$; explicitly, ${\mathcal{F}\mathcal{Z}}^\vee \subset {\mathbb {Q}}\langle x,y\rangle$ is a weight-graded space in which ${\mathcal{F}\mathcal{Z}}_0^\vee ={\mathbb {Q}}$, ${\mathcal{F}\mathcal{Z}}^\vee _1=0$ and for $w\ge 2$, ${\mathcal{F}\mathcal{Z}}^\vee _w$ consists of all degree *w* homogeneous polynomials $P\in {\mathbb {Q}}\langle x,y\rangle$ satisfying

3.2 $$\begin{aligned} \langle L,P\rangle =0\ \ \hbox {for all }L\in {\mathcal {I}}_{\mathcal{F}\mathcal{Z}}, \end{aligned}$$

for the pairing in (3.1) (see Definition 2.1.1 for an explicit description of the elements *L* of the ideal ${\mathcal {I}}_{\mathcal{F}\mathcal{Z}}$). The subspace ${\mathcal{F}\mathcal{Z}}^\vee$ is strictly smaller than ${\mathbb {Q}}\langle x,y\rangle$. In weight $w=2$, for instance, since $Z(xy)+Z(yx) \in {\mathcal {I}}_{{\mathcal{F}\mathcal{Z}}}$, we have $xy-yx \in {\mathcal{F}\mathcal{Z}}^\vee _2$ whereas *xy* and *yx* are not individually contained in ${\mathcal{F}\mathcal{Z}}^\vee _2$.

Similarly, the dual space of the quotient ${\overline{{\mathcal{F}\mathcal{Z}}}}$ of ${\mathcal{F}\mathcal{Z}}$ modulo $\zeta ^{\mathfrak {f}}_2$ is a subspace ${\overline{{\mathcal{F}\mathcal{Z}}}}^\vee \subset {\mathcal{F}\mathcal{Z}}^\vee$. We now consider ${\overline{{\mathcal{F}\mathcal{Z}}}}$ with its Hopf algebra structure given by the coproduct $\Delta ^G$; then the dual space ${\overline{{\mathcal{F}\mathcal{Z}}}}^\vee$ is also a Hopf algebra. The coproduct on ${\overline{{\mathcal{F}\mathcal{Z}}}}^\vee$ is inherited directly from the standard coproduct $\Delta _s$ on ${\mathbb {Q}}\langle x,y\rangle$, given by

3.3 $$\begin{aligned} \Delta _s(x)=x\otimes \textbf{1}+\textbf{1}\otimes x , \ \ \ \ \Delta _s(y)=y\otimes \textbf{1}+\textbf{1}\otimes y\,; \end{aligned}$$

it satisfies3.4for $g\in {\overline{{\mathcal{F}\mathcal{Z}}}}^\vee$, $\xi _1,\xi _2\in {\overline{{\mathcal{F}\mathcal{Z}}}}$. The multiplication on ${\overline{{\mathcal{F}\mathcal{Z}}}}^\vee$, which we denote by $\diamond$, is uniquely determined by the equality

3.5 $$\begin{aligned} \langle \Delta ^G(\xi ),g\otimes h\rangle =\langle \xi ,g\diamond h\rangle \end{aligned}$$

for $\xi \in {\overline{{\mathcal{F}\mathcal{Z}}}}$ and $g,h\in {\overline{{\mathcal{F}\mathcal{Z}}}}^\vee$, see Prop. 3.18 of [58] for a fully explicit proof of this duality formula. An explicit formula for $g\diamond h$ in the restricted case of $g \in {\mathfrak {fz}}^\vee$ can be found in (3.17) below; the full formula (which we do not need here) can be found in (23) of [58].

Let us now explain how to view ${\overline{{\mathcal{F}\mathcal{Z}}}}^\vee$ as the universal enveloping algebra of the Lie algebra consisting of its primitive elements. We begin by identifying the subspace $\textrm{Lie}[x,y]$ of Lie polynomials in ${\mathbb {Q}}\langle x,y\rangle$ as the subspace of primitive elements, which are those satisfying

3.6 $$\begin{aligned} \Delta _s(g)=g\otimes \textbf{1}+\textbf{1}\otimes g . \end{aligned}$$

An equivalent formulation of this property is that *g* is a Lie polynomial in ${\mathbb {Q}}\langle x,y\rangle$ if and only if3.7for all pairs of non-empty words *u*, *v*. The Lie subalgebra of the Hopf algebra ${\overline{{\mathcal{F}\mathcal{Z}}}}^\vee$ is likewise the space of elements $g\in {\overline{{\mathcal{F}\mathcal{Z}}}}^\vee$ satisfying (3.6); the Lie bracket is given by

3.8 $$\begin{aligned} \{g,h\} :=g\diamond h-h\diamond g , \end{aligned}$$

for the multiplication $\diamond$ of (3.5).

This Lie algebra is identified with the dual of the space ${\mathfrak {fz}}$ defined in (2.51) above; indeed, the vector space ${\mathfrak {fz}}$ inherits the structure of a Lie coalgebra from the Hopf algebra structure on ${\overline{{\mathcal{F}\mathcal{Z}}}}$, so its dual space ${\mathfrak {fz}}^\vee \subset {\overline{{\mathcal{F}\mathcal{Z}}}}^\vee$ thus forms a Lie algebra, which is precisely the Lie algebra of primitive elements of ${\overline{{\mathcal{F}\mathcal{Z}}}}^\vee$.

Since ${\mathfrak {fz}}$ is the quotient of ${\overline{{\mathcal{F}\mathcal{Z}}}}$ modulo non-trivial products and the relations3.9hold in ${\mathcal{F}\mathcal{Z}}$ (the second equality being valid whenever *u*, *v* both end in *y*), we see that the images of $\zeta ^{\mathfrak {f}}(w)$ in the quotient ${\mathfrak {fz}}$ satisfy3.10Thus the dual space ${\mathfrak {fz}}^\vee$ is the subspace of polynomials $g\in {\mathbb {Q}}\langle x,y\rangle$ such that3.11for all pairs of monomials *u* and *v* (ending in *y* for the $*$ term), where

3.12 $$\begin{aligned} g_*=g+\sum _{n\ge 2} \frac{(-1)^{n-1}}{n}\zeta ^{\mathfrak {f}}(x^{n-1}y) y^n , \end{aligned}$$

(the term added to *g* is the linearized version of (2.10)). We note in particular that by (3.7), the first equality  shows that we have an inclusion of vector spaces (which is not a Lie algebra morphism as the brackets are different)

3.13 $$\begin{aligned} {\mathfrak {fz}}^\vee \subset \textrm{Lie}[x,y] .\end{aligned}$$

The Lie algebra ${\mathfrak {fz}}^\vee$ is known as the *double shuffle Lie algebra* and usually denoted by ${\mathfrak {ds}}$ for “double shuffle” (or ${\mathfrak {dmr}}$ for “double mélange régularisé” by French authors). The Lie bracket $\{\cdot ,\cdot \}$ on ${\mathfrak {ds}}$ corresponds to the Ihara bracket

3.14 $$\begin{aligned} \{g,h\}=[g,h]+D_g(h)-D_h(g) , \end{aligned}$$

where for each $g\in \textrm{Lie}[x,y]$, the *Ihara derivation* $D_g$ of $\textrm{Lie}[x,y]$ is defined by

3.15 $$\begin{aligned} D_g(x)=0 , \ \ \ \ D_g(y)=[y,g] , \end{aligned}$$

and the Lie bracket arises from the bracket of derivations

3.16 $$\begin{aligned} {[}D_g,D_h]=D_{\{g,h\}} . \end{aligned}$$

The Hopf algebra ${\overline{{\mathcal{F}\mathcal{Z}}}}^\vee$ is identified with the universal enveloping algebra ${\mathcal {U}}{\mathfrak {ds}}$. As such, the multiplication $\diamond$ is identified with the Poincaré–Birkhoff–Witt multiplication (which exists for every universal enveloping algebra of a Lie algebra).

In the case where $g\in {\mathfrak {ds}}$ and $h\in {\mathcal {U}}{\mathfrak {ds}}$ the multiplication $\diamond$ can be written succinctly as

3.17 $$\begin{aligned} g\diamond h=gh+D_{g}(h) , \end{aligned}$$

which suffices for our purposes and implies that the two representations (3.8) and (3.14) of the Ihara bracket agree.

In the rest of this article with the exception of Sect. 4, we will consider the space ${\mathcal{F}\mathcal{Z}}$ as a Hopf algebra comodule equipped with the coaction $\Delta ^{GB}$ over the Hopf algebra ${\overline{{\mathcal{F}\mathcal{Z}}}}$ equipped with the coproduct $\Delta ^G$; the multiplication $\diamond$ extends to ${\mathcal{F}\mathcal{Z}}$ by the identity

3.18 $$\begin{aligned} \langle \Delta ^{GB}(\xi ),g\otimes h\rangle = \langle \xi ,g\diamond h\rangle \end{aligned}$$

for $\xi \in {\mathcal{F}\mathcal{Z}}$ and $g,h\in {\mathcal{F}\mathcal{Z}}^\vee$. The quotient space ${\overline{{\mathcal{M}\mathcal{Z}}}}$ of ${\overline{{\mathcal{F}\mathcal{Z}}}}$ is then also a Hopf algebra equipped with the coproduct $\Delta ^G$, and ${\mathcal{M}\mathcal{Z}}$ equipped with $\Delta ^{GB}$ is a Hopf algebra comodule over it. The dual space

3.19 $$\begin{aligned} {\overline{{\mathcal{M}\mathcal{Z}}}}^\vee \subset {\overline{{\mathcal{F}\mathcal{Z}}}}^\vee = {{\mathcal {U}}}{\mathfrak {ds}}\end{aligned}$$

of ${\overline{{\mathcal{M}\mathcal{Z}}}}$ is a Hopf algebra equipped with the standard coproduct $\Delta _s$ and the (restriction of the) multiplication $\diamond$, and the Lie algebra

3.20 $$\begin{aligned} {\mathfrak {mz}}^\vee \subset {\mathfrak {fz}}^\vee = {\mathfrak {ds}}\end{aligned}$$

consists of the primitive elements for $\Delta _s$ in ${\overline{{\mathcal{M}\mathcal{Z}}}}$, and is equipped with the (restriction of the) Ihara bracket (3.8).

### The Z-map and dual spaces

#### Definition 3.2.1

We define the *Z-map* to be the canonical isomorphism3.21mapping $\textbf{1}$ to 1 and each non-trivial monomial *w* to *Z*(*w*), so that the notation *Z*(*w*), previously just a symbol (see Sect. 2.1.2), can now be interpreted as the image of the monomial *w* under the map *Z*. The Z-map restricts to a canonical isomorphism on each (finite-dimensional) weight-graded part, and passes to corresponding isomorphisms (also called Z-maps) between any quotient of ${\mathbb {Q}}[Z(w)]$ (in particular the MZV spaces) and its dual viewed as a subspace of ${\mathbb {Q}}\langle x,y\rangle$.

The situation is summarized in (3.25) below, in which all of the horizontal arrows are the canonical isomorphisms inherited from the top Z-map

3.22 $$\begin{aligned} Z:{\mathbb {Q}}\langle x,y\rangle \rightarrow {\mathbb {Q}}[Z(w)] , \end{aligned}$$

all surjective maps are quotients, and all injective maps are inclusions of the dual spaces. The space ${\overline{{\mathcal {Z}}}}$ denotes the quotient of the ${\mathbb {Q}}$-algebra ${\mathcal {Z}}$ of real MZVs modulo the ideal generated by $\zeta _2$, and in analogy with ${\mathfrak {fz}}$ and ${\mathfrak {mz}}$, we denote the quotient of ${\overline{{\mathcal {Z}}}}$ mod constants and non-trivial products by ${\mathfrak {z}}$.

For instance, the Z-map *Z*(*xy*) is given by $\zeta _2^{\mathfrak {m}}$ in $\mathcal{M}\mathcal{Z}$ and 0 in $\overline{\mathcal{M}\mathcal{Z}}$, respectively. More generally, we have

3.23 $$\begin{aligned} Z(x^{k_r-1}y\cdots x^{k_2-1}y x^{k_1-1}y) =\zeta ^{\mathfrak {m}}_{k_1,k_2,\ldots ,k_r}\ \textrm{in} \ \mathcal{M}\mathcal{Z} \end{aligned}$$

for convergent words ($k_r\ge 2$), whereas the Z-map of divergent words follows from setting the combinations in (2.18) to zero.

Note that while both ${\mathfrak {fz}}$ and ${\mathfrak {mz}}$ are equipped with a Lie coalgebra structure inherited from the Hopf algebra structures on ${\overline{{\mathcal{F}\mathcal{Z}}}}$ and ${\overline{{\mathcal{M}\mathcal{Z}}}}$, we do not know that ${\overline{{\mathcal {Z}}}}$ is a Hopf algebra and therefore we do not know that ${\mathfrak {z}}$ has a Lie coalgebra structure. However we still have vector space surjections ${\mathfrak {fz}}\rightarrow \!\!\!\!\!\rightarrow {\mathfrak {mz}}\rightarrow \!\!\!\!\!\rightarrow {\mathfrak {z}}$ and the corresponding vector space inclusions of the dual spaces, all of which lie in the vector space $\textrm{Lie}[x,y]$ by (3.13):

3.24 $$\begin{aligned} {\mathfrak {z}}^\vee \subset {\mathfrak {mz}}^\vee \subset {\mathfrak {fz}}^\vee \subset \textrm{Lie}[x,y] . \end{aligned}$$

We underline once more that all maps in the following diagram are to be viewed as vector space morphisms.3.25We will make constant use of the Z-maps as well as the quotient maps and inclusions in this diagram for our constructions below.

### The canonical decomposition of motivic MZV spaces and zeta generators in genus zero

In this section we will define a specific canonical decomposition of ${\mathcal{M}\mathcal{Z}}_w$ for each weight $w\ge 2$ into singles, irreducibles and reducibles of the type

3.26 $$\begin{aligned} {\mathcal{M}\mathcal{Z}}_w={\mathbb {Q}}\zeta ^{\mathfrak {m}}_w\oplus I_w\oplus R_w \end{aligned}$$

introduced in (2.56). The existence of this decomposition relies on working in the space of motivic multizeta values. More generally, the main results of this work are stated only for motivic multizetas as opposed to real multizetas in (2.1) since our arguments and proofs crucially rely on the freeness of the Lie algebra ${\mathfrak {mz}}^\vee$ below which is tied to the *motivic* incarnation of multizetas.

#### Definition 3.3.1

For each $w\ge 2$, let ${\hat{R}}_w\subset {\mathcal{M}\mathcal{Z}}_w$ denote the subspace of reducible MZVs as in Sect. 2.2.2, let ${\mathfrak {mz}}_w={\mathcal{M}\mathcal{Z}}_w/{\hat{R}}_w$ as in (2.54), let ${\mathfrak {mz}}_w^\vee \subset {\mathcal{M}\mathcal{Z}}_w^\vee$ denote the dual space, and let $({\mathfrak {mz}}_w^\vee )^{\ge 2} \subset {\mathfrak {mz}}_w^\vee$ denote the subspace of ${\mathfrak {mz}}_w^\vee$ consisting of elements of depth $\ge 2$, where depth is the minimal *y*-degree of a polynomial.

- Define the *canonical subspace of non-single irreducibles* $I_w$ of ${\mathcal{M}\mathcal{Z}}_w$ by
  3.27 $$\begin{aligned} I_w=Z\bigl (({\mathfrak {mz}}_w^\vee )^{\ge 2}\bigr )\subset {\mathcal{M}\mathcal{Z}}_w . \end{aligned}$$
- Define the *canonical subspace of non-single reducibles* $R_w$ as follows. For odd weights *w*, set $R_w={\hat{R}}_w$, and for even weights *w*, let $R_w\subset {\hat{R}}_w$ be the subspace spanned by all weight *w* products of the elements: $\zeta ^{\mathfrak {m}}_2$, the single zetas $\zeta ^{\mathfrak {m}}_v$ for odd $v<w$, and all elements of $I_v$ with $v<w$, excluding only the product $(\zeta ^{\mathfrak {m}}_2)^{w/2}$. Then since ${\mathcal{M}\mathcal{Z}}= {\mathbb {Q}}[\zeta ^{\mathfrak {m}}_2]\otimes _{\mathbb {Q}}{\overline{{\mathcal{M}\mathcal{Z}}}}$ (cf. (2.41)), using (2.53), we have ${\hat{R}}_w={\mathbb {Q}}\zeta ^{\mathfrak {m}}_{w}\oplus R_w$ when *w* is even.
- Define the *canonical decomposition* of ${\mathcal{M}\mathcal{Z}}_w$ to be
  3.28 $$\begin{aligned} {\mathcal{M}\mathcal{Z}}_w={\mathbb {Q}}\zeta ^{\mathfrak {m}}_w\oplus I_w\oplus R_w \end{aligned}$$
  for the canonical subspaces $R_w$ and $I_w$ defined above.
- Finally, define the *canonical polynomial* $g_w\in {\mathcal{M}\mathcal{Z}}_w^\vee$ for each $w\ge 2$ to be the unique polynomial in *x*, *y* that
  - takes the value 1 on $\zeta ^{\mathfrak {m}}_w =\zeta ^{\mathfrak {m}}(x^{w-1}y)$ in the sense that $\langle Z(x^{w-1}y),g_w\rangle =1$, and
  - annihilates $I_w$ and $R_w$ in the sense that $\langle \xi ,g_w\rangle =0$ for any $\xi \in I_w$ and $\xi \in R_w$.
  That such polynomials exist follows from Lemma 3.3.2, but also from their alternative characterization in terms of the Drinfeld associator, see Sect. 3.6 below.

Examples of the polynomials $g_w$ will be given in Sect. 3.4 below.

#### Lemma 3.3.2

The canonical polynomials $g_w$ for $w\ge 2$ are uniquely characterized by the following properties:

(i) The polynomial $g_w$ is normalized by $g_w|_{x^{w-1}y}=1$;
(ii) The polynomial $g_w$ lies in the subspace $({\mathcal{M}\mathcal{Z}}_w/R_w)^\vee \subset {\mathcal{M}\mathcal{Z}}_w^\vee$; in particular for odd *w* it lies in ${\mathfrak {mz}}_w$ and is thus a Lie polynomial;
(iii) If we consider $g_w$ as lying in $({\mathcal{M}\mathcal{Z}}_w/R_w)^\vee$, the image $Z(g_w)$ of $g_w$ under the Z-map is a rational multiple of $\zeta ^{\mathfrak {m}}_w\in {\mathcal{M}\mathcal{Z}}_w/R_w$; equivalently, if we consider $g_w$ as lying in ${\mathcal{M}\mathcal{Z}}_w^\vee$, then $Z(g_w)$ does not contain any irreducible multizeta values in $I_w$
  3.29 $$\begin{aligned} Z(g_w)\in {\mathbb {Q}}\zeta ^{\mathfrak {m}}_w\oplus R_w\subset {\mathcal{M}\mathcal{Z}}_w . \end{aligned}$$

#### Proof

(i) is equivalent to $\langle Z(x^{w-1}y),g_w\rangle =1$.

For (ii), saying that $g_w$ annihilates $R_w$ is equivalent to saying that $g_w$ lies in the dual space of ${\mathcal{M}\mathcal{Z}}_w/R_w$, namely $({\mathcal{M}\mathcal{Z}}_w/R_w)^\vee$; this space is equal to ${\mathfrak {mz}}_w^\vee$ when *w* is odd, so by (3.24) $g_w$ is then in $\textrm{Lie}[x,y]$.

For (iii), we consider $g_w\in ({\mathcal{M}\mathcal{Z}}_w/R_w)^\vee$ and for ${\mathcal{M}\mathcal{Z}}_w/R_w = {\mathbb {Q}}\zeta ^{\mathfrak {m}}_w\oplus I_w$ we choose any basis consisting of $\zeta ^{\mathfrak {m}}_w$ and a basis for $I_w$. Then since $\langle g_w,I_w\rangle =0$ for all $\xi \in I_w$ we have $\langle Z(g_w),Z^{-1}(I_w)\rangle =0$, but $Z^{-1}(I_w)= ({\mathfrak {mz}}_w^\vee )^{\ge 2}$, and the subspace of ${\mathcal{M}\mathcal{Z}}_w/R_w$ annihilated by $({\mathfrak {mz}}_w^\vee )^{\ge 2}$ is the 1-dimensional subspace generated by $\zeta ^{\mathfrak {m}}_w$. Therefore if $g_w$ is considered as lying in $({\mathcal{M}\mathcal{Z}}_w/R_w)^\vee$ we have $Z(g_w)\in {\mathbb {Q}}\zeta ^{\mathfrak {m}}_w\subset {\mathcal{M}\mathcal{Z}}_w/R_w$, or equivalently, if $g_w$ is considered as lying in ${\mathcal{M}\mathcal{Z}}_w^\vee$, we have $Z(g_w)\in {\mathbb {Q}}\zeta ^{\mathfrak {m}}_w\oplus R_w$. This construction proves the uniqueness of $g_w$: the 1-dimensional subspace it generates annihilates the subspace $({\mathfrak {mz}}_w^\vee )^{\ge 2}$ of non-single zetas in the dual, and the specific choice of $g_w$ is given by the normalization in (i). $\square$

#### Remark 3.3.3

The lemma shows that in order to compute the canonical polynomials $g_w$ for any $w\ge 2$, once conditions (i) and (ii) of Lemma 3.3.2 are fulfilled, the third defining condition of $g_w$, namely that it annihilates the subspace $I_w$, can be replaced by condition (iii) of the Lemma, which does not require computing the space $I_w$. Once $g_w$ is determined, it is then possible to recover the space $I_w$ as the image of a Lie subspace of ${\mathcal{M}\mathcal{Z}}^\vee$ under *Z* as in (3.27) if needed. We will actually provide a very natural explicit basis for $I_w$, called *the semi-canonical basis*, in Sect. 3.5 below.

#### Remark 3.3.4

As mentioned in Remark 1.1.4 in the introduction, Keilthy has provided a construction of polynomials $g_w$ for odd *w* in his dissertation [25]. The idea of his method is the following: assuming that the $g_w$ have been chosen for odd *w* up to $2k-1$, one can then fix a choice of $g_{2k+1}$ by requiring it to be orthogonal to all weight $2k+1$ Ihara brackets of the previously chosen $g_w$; for example, $g_{11}$ is fixed by the unique condition that it must be orthogonal to $\{g_3,\{g_3,g_5\}\}$, where orthogonality is defined by the inner product on all pairs of monomials *u*, *v* taking value $\delta _{u,v}$, analogously to our (3.1). This is equivalent to our condition $\langle \xi , g_w\rangle =0$ for all irreducibles $\xi \in I_w$ since a basis of $I_w$ can be given using Ihara brackets of lower degree $g_{w'}$ (i.e. with $w'<w$). Note however that Lemma 3.3.2 provides canonical elements $g_w$ also for even *w*.

Given that we know from [2] that ${\mathfrak {mz}}^\vee$ is free on one depth 1 generator in each odd weight $w\ge 3$ and the $g_w$ are such elements, the set of $g_w$ for odd $w\ge 3$ form a canonical generating set for ${\mathfrak {mz}}^\vee$. By Lemma 3.3.2, each $g_w$ is characterized uniquely as the only depth 1 element of ${\mathfrak {mz}}_w^\vee \subset {\mathcal{M}\mathcal{Z}}^\vee$ normalized by $g_w|_{x^{w-1}y}=1$ such that $Z(g_w) \in {\mathbb {Q}}\zeta ^{\mathfrak {m}}_w\oplus R_w\subset {\mathcal{M}\mathcal{Z}}_w$.

#### Definition 3.3.5

The Ihara derivations (3.15) associated with the $g_w$ with $w\ge 3$ odd are referred to as *zeta generators in genus zero*.

The method of using the Z-map to produce canonical generators by taking the duals of the single zetas was initially developed in the framework of formal multizetas in [20]. The family of polynomials $g_w$ will play a crucial role in the main results of this paper, namely

- the construction of a canonical isomorphism $\rho :{\mathcal{M}\mathcal{Z}}\rightarrow {{\mathcal {F}}}$ from the motivic MZVs to the *f*-alphabet (Sect. 4.2);
- the construction of a canonical set of zeta generators in genus one (Sect. 5.3).

In the next subsection we give the explicit calculation of the canonical decomposition in weights up to $w=11$ and spell out the canonical polynomials $g_w$ up to $w=7$. We emphasize that our approach is unaffected by the irregular behaviour of the depth filtration of MZVs starting in weight 12: Our method to construct the canonical polynomials $g_w$ does not depend on the depth of the MZVs encountered in the canonical decompositions for ${\mathcal{M}\mathcal{Z}}_w$, and the explicit form of $g_{12}$ can be found in the ancillary files of the arXiv submission. The canonical morphism to the *f*-alphabet resulting from the discussion of Sect. 4 below and relying on the canonical decomposition of ${\mathcal{M}\mathcal{Z}}_w$ in intermediate steps is explicitly worked out up to and including weight 17 in the ancillary files of [12].

### The canonical decomposition for ${\mathcal{M}\mathcal{Z}}_w$ for $w\le 11$

Since all MZVs in this subsection and the next one are motivic, we drop the superscript $\mathfrak {m}$ and simply write $\zeta _{k_1,\ldots ,k_r}$ instead of $\zeta ^{\mathfrak {m}}_{k_1,\ldots ,k_r}$. We have

3.30 $$\begin{aligned} {\mathcal{M}\mathcal{Z}}_2&=\langle \zeta _2\rangle , \nonumber \\ {\mathcal{M}\mathcal{Z}}_3&=\langle \zeta _3\rangle , \nonumber \\ {\mathcal{M}\mathcal{Z}}_4&=\langle \zeta _4\rangle ,\nonumber \\ {\mathcal{M}\mathcal{Z}}_5&=\langle \zeta _5\rangle \oplus \langle \zeta _2\zeta _3\rangle ={\mathbb {Q}}\zeta _5\oplus R_5 ,\nonumber \\ {\mathcal{M}\mathcal{Z}}_6&=\langle \zeta _6\rangle \oplus \langle \zeta _3^2\rangle ={\mathbb {Q}}\zeta _6\oplus R_6 ,\nonumber \\ {\mathcal{M}\mathcal{Z}}_7&=\langle \zeta _7\rangle \oplus \langle \zeta _2\zeta _5 ,\ \zeta _2^2\zeta _3\rangle ={\mathbb {Q}}\zeta _7\oplus R_7 ,\nonumber \\ {\mathcal{M}\mathcal{Z}}_8&=\langle \zeta _8\rangle \oplus \langle Z_{35}\rangle \oplus \langle \zeta _3\zeta _5 ,\ \zeta _2\zeta _3^2\rangle ={\mathbb {Q}}\zeta _8\oplus I_8\oplus R_8 ,\nonumber \\ {\mathcal{M}\mathcal{Z}}_9&=\langle \zeta _9\rangle \oplus \langle \zeta _3^3 ,\ \zeta _2\zeta _7 ,\ \zeta _4\zeta _5 ,\ \zeta _6\zeta _3\rangle ={\mathbb {Q}}\zeta _9\oplus R_9\, \nonumber \\ {\mathcal{M}\mathcal{Z}}_{10}&=\langle \zeta _{10}\rangle \oplus \langle Z_{37}\rangle \oplus \langle \zeta _3\zeta _7 ,\ \zeta _5^2 ,\ \zeta _2\zeta _3\zeta _5 ,\ \zeta _2Z_{35} ,\ \zeta _4\zeta _3^2 \rangle ={\mathbb {Q}}\zeta _{10}\oplus I_{10}\oplus R_{10} ,\nonumber \\ {\mathcal{M}\mathcal{Z}}_{11}&=\langle \zeta _{11}\rangle \oplus \langle Z_{335}\rangle \oplus \langle \zeta _3Z_{35} ,\ \zeta _3^2\zeta _5 ,\ \zeta _2\zeta _9 ,\ \zeta _2\zeta _3^3 ,\ \zeta _4\zeta _7 ,\ \zeta _6\zeta _5 ,\ \zeta _8\zeta _3\rangle \nonumber \\&={\mathbb {Q}}\zeta _{11}\oplus I_{11}\oplus R_{11} , \end{aligned}$$

where the irreducibles $Z_{35}$, $Z_{37}$ and $Z_{335}$ are the Z-map images of the generators $\{g_3,g_5\}$, $\{g_3,g_7\}$ and $\{g_3,\{g_3,g_5\}\}$ of $({\mathfrak {mz}}_w^\vee )^{\ge 2}$ for $w=8,10$ and 11, respectively (see (3.14) for the definition of the Ihara bracket): they are explicitly given in terms of a common (arbitrary) choice of MZVs $\zeta _{3,5}$, $\zeta _{3,7}$ and $\zeta _{3,3,5}$ by

3.31 $$\begin{aligned} Z_{35}&:=Z(\{g_3,g_5\})= -\tfrac{1105181}{80}\zeta _8 +\tfrac{24453}{5}\zeta _{3,5} +\tfrac{28743}{2}\zeta _3\zeta _5-1683\,\zeta _2\zeta _3^2 ,\nonumber \\ Z_{37}&:=Z(\{g_3,g_7\})= \tfrac{6614309}{112} \zeta _{3,7} + \tfrac{7796217}{16}\zeta _3\zeta _7 + \tfrac{26525967}{112} \zeta _5^2-\tfrac{2159}{627}\zeta _2 Z_{35} \nonumber \\&\qquad -\tfrac{3203187}{76}\zeta _2\zeta _3\zeta _5 - \tfrac{60072829}{608} \zeta _4 \zeta _3^2 -\tfrac{408872741707}{680960} \zeta _{10} ,\nonumber \\ Z_{335}&:=Z(\{g_3,\{g_3,g_5\}\}) = -\tfrac{3683808}{5}\zeta _{3,3,5} +\tfrac{1119631493}{20}\zeta _{11}-\tfrac{28597725}{38}\zeta _3^2\zeta _5\nonumber \\&\qquad +\tfrac{296304}{2717} \zeta _3 Z_{35}-\tfrac{198893689}{6}\zeta _2\zeta _9 +\tfrac{25828428}{247}\zeta _2\zeta _3^3 - \tfrac{90515817}{40} \zeta _4\zeta _7 \nonumber \\&\qquad + \tfrac{6826931}{4} \zeta _6\zeta _5 + \tfrac{1953356831 }{23712} \zeta _8\zeta _3 . \end{aligned}$$

We observe here that the products listed above spanning the spaces of reducibles $R_w$ actually form bases for these spaces. This is a general result valid for all *w*, which will be proven in the following Sect. 3.5, in which we actually determine an explicit basis for ${\mathcal{M}\mathcal{Z}}$ adapted to the canonical decomposition of Definition 3.3.1.

Up to $w=7$, the canonical polynomials $g_w$ are given by

3.32 $$\begin{aligned} g_2&=[xy] ,\nonumber \\ g_3&=[x[xy]]+[[xy]y] , \nonumber \\ g_4&=[x[x[xy]]]+\tfrac{1}{4}[x[[xy]y]]+[[[xy]y]y]+\tfrac{5}{4}(xyxy-xyyx-yxxy+yxyx) ,\nonumber \\ g_5&=[x[x[x[xy]]]]{+}2[x[x[[xy]y]]]{-}\tfrac{3}{2}[[x[xy]]\, [xy]]{+}2[x[[[xy]y]y]]\nonumber \\&\quad {+}\tfrac{1}{2}[[xy]\, [[xy]y]]{+}[[[[xy]y]y]y] ,\nonumber \\ g_6&=[x[x[x[x[xy]]]]]{+}\tfrac{3}{4}[x[x[x[[xy]y]]]]{+}\tfrac{1}{6}[x[[x[xy]]\,[xy]]]\nonumber \\&\quad {+}\tfrac{23}{16}[x[x[[[xy]y]y]]]{+}\tfrac{1}{12}[x[[xy]\,[[xy]y]]]\nonumber \\&\quad -\tfrac{89}{48}[x[[[xy]y]\,[xy]]]+\tfrac{3}{4}[x[[[[xy]y]y]y]] +\tfrac{5}{3}[[xy]\,[[[xy]y]y]]+[[[[[xy]y]y]y]y] \nonumber \\&\quad +\tfrac{7}{4}(xyxxxy-xyyxxx+xyyyxy-xyyyyx\nonumber \\&\quad -yxxxxy+yxyxxx-yyyxxy+yyyxyx)\nonumber \\&\quad +\tfrac{21}{4} (xyxyxx-xyxxyx+yxxxyx-y\nonumber \\&\quad -yxyyxy+yxyyyx+yyxyxy-yyxyyx)\nonumber \\&\quad +\tfrac{7}{16}(xyxxyy-xyyyxx-yxxxyy+yxyyxx)+\tfrac{7}{48}(yxxyxy-xyxyxy)\nonumber \\&\quad +\tfrac{35}{48}(yxxyyx+yxyxxy-xyxyyx-xyyxxy)+\tfrac{77}{48}(xyyxyx-yxyxyx) ,\nonumber \\ g_7&=[x[x[x[x[x[xy]]]]]]+3[x[x[x[x[[xy]y]]]]] \nonumber \\&\quad -5 [x [x[[x[x, y]]\,[x, y]]]] +2[[x[x[xy]]\,[x[xy]]] \nonumber \\&\quad +5 [x[x[x[[[x y] y] y]]]] +\tfrac{19}{16}[x[x[[xy]\,[[xy]y]]]] -\tfrac{173}{16} [x[[x[[x y] y]]\, [x y]]]\nonumber \\&\quad -2[[x[xy]]\,[x[[xy]y]]]\nonumber \\&\quad +\tfrac{17}{16}[[[x[xy]]\,[xy]]\,[xy]]+5[x[x[[[[xy]y]y]y]]] +\tfrac{99}{16}[x[[xy]\,[[[xy]y]y]]]\nonumber \\&\quad -\tfrac{61}{16}[[x[[xy]y]]\,[[xy]y]]\nonumber \\&\quad -\tfrac{109}{16}[[x[[[xy]y]y]]\,[xy]] +\tfrac{65}{16}[[xy]\,[[xy]\,[[xy]y]]]\nonumber \\&\quad +3[x[[[[[xy]y]y]y]y]]+4[[xy]\,[[[[xy]y]y]y]]\nonumber \\&\quad +3[[[xy]y]\,[[[xy]y]y]] +[[[[[[xy]y]y]y]y]y] . \end{aligned}$$

In these expressions, we have omitted the separating comma between the two arguments of the Lie bracket in $\textrm{Lie}[x,y]$ to condense the formulas. The odd degree (Lie) polynomials satisfy the symmetry property $g_{2k+1}(x,y) = g_{2k+1}(y,x)$ that follows from the arguments in footnote 12. This is easy to see for $g_3$, but requires also the use of the Jacobi identity to make it manifest for $g_5$ and $g_7$. Our expressions are chosen to be adapted to the Lyndon basis of $\textrm{Lie}[x,y]$ that we introduce in the next section.

For $w\ge 8$ the polynomials $g_w$ become too unwieldy to write down, although they can be calculated on a computer easily (either by the methods presented here, or from the Drinfeld associator as in (3.49) below). The explicit form of all $g_w$ at $w \le 12$ can be found in machine-readable form in an ancillary file of the arXiv submission of this work. However, since the Z-map is an isomorphism, no information is lost in giving their Z-map images, which determine them completely and are much shorter to write down:

3.33 $$\begin{aligned} Z(g_2)&=2 \zeta _2 ,\nonumber \\ Z(g_3)&=12\zeta _3 ,\nonumber \\ Z(g_4)&=\tfrac{375}{8}\zeta _4 ,\nonumber \\ Z(g_5)&=385\zeta _5-105\zeta _2\zeta _3 ,\nonumber \\ Z(g_6)&=\tfrac{251797}{288}\zeta _6-\tfrac{679}{4}\zeta _3^2 ,\nonumber \\ Z(g_7)&=\tfrac{49203}{4}\zeta _7 -\tfrac{14091}{4}\zeta _2\zeta _5 -\tfrac{11865}{4}\zeta _4\zeta _3 ,\nonumber \\ Z(g_8)&=\tfrac{769152355481}{40974336}\zeta _8-\tfrac{18246083}{1824}\zeta _3\zeta _5+\tfrac{74974943}{71136}\zeta _2\zeta _3^2 ,\nonumber \\ Z(g_9)&=\tfrac{373659143}{864}\zeta _9-\tfrac{264398849}{3456}\zeta _6\zeta _3-\tfrac{3702413}{36}\zeta _4\zeta _5-\tfrac{70513729}{576}\zeta _2\zeta _7+\tfrac{133133}{16}\zeta _3^3 ,\nonumber \\ Z(g_{10})&=\tfrac{22565838727030761032761}{48180785666457600} \zeta _{10} + + \tfrac{23603271373}{184515876480} \zeta _2 Z_{35} \nonumber \\&\quad -\tfrac{70504768535925229}{227096463360} \zeta _3\zeta _7 -\tfrac{66965094752611}{436723968} \zeta _5^2 \nonumber \\&\quad +\tfrac{21865877274704331}{321719989760} \zeta _2\zeta _3\zeta _5 + \tfrac{3916397111572098571}{100376636805120} \zeta _4 \zeta _3^2 ,\nonumber \\ Z(g_{11})&=\tfrac{1316030287522093}{78587904}\zeta _{11} +\tfrac{67235}{1227936}\zeta _3Z_{35} \nonumber \\&\quad +\tfrac{4632642114815}{4911744}\zeta _3^2\zeta _5 -\tfrac{824237896586533}{176822784}\zeta _2\zeta _9 \nonumber \\&\quad -\tfrac{470709526441}{4911744}\zeta _2\zeta _3^3 -\tfrac{3026492983085}{818624}\zeta _4\zeta _7\nonumber \\&\quad -\tfrac{218501860145855}{78587904}\zeta _6\zeta _5 -\tfrac{3190686062952839}{1414582272}\zeta _8\zeta _3 . \end{aligned}$$

Note that, in agreement with the third characterizing property (3.29) of $g_w$, the non-single irreducibles $Z_{35}\in I_8$, $Z_{37}\in I_{10}$ and $Z_{335}\in I_{11}$ are absent in $Z(g_{8})$, $Z(g_{10})$ and $Z(g_{11})$, respectively. The contributions $\zeta _2Z_{35}$ and $\zeta _3Z_{35}$ to $Z(g_{10})$ and $Z(g_{11})$ lie in $R_{10}$ and $R_{11}$, respectively, and are therefore compatible with (3.29).

### The semi-canonical basis for ${\mathcal{M}\mathcal{Z}}_w$

In this section we determine an explicit basis for ${\mathcal{M}\mathcal{Z}}$ which is adapted to the canonical decomposition. The basis of the irreducible parts $I_w$ is given by the Z-map images of the Lyndon brackets of the canonical free generators $g_w$ of ${\mathfrak {mz}}_w^\vee$. The basis of the reducible part $R_w$ in turn consists of all weight *w* products of elements of the set given by $\zeta _2$, $\zeta _v$ for all odd $v<w$, and the chosen basis elements for $I_v$ for $v<w$, which form a linearly independent set as proven in Corollary 3.5.8 at the end of this subsection. Because the Lyndon basis for a free Lie algebra, although very natural and practical, cannot justifiably be called canonical, we refer to our basis as the *semi-canonical* basis for the canonical decomposition of ${\mathcal{M}\mathcal{Z}}_w$.

Let us recall the definition and the basic result we need concerning Lyndon bases.

#### Definition 3.5.1

Let $B=\{b_1,b_2,\ldots \}$ be an ordered set of letters. A *Lyndon word* in the alphabet *B* is a word $W_1=b_{i_1}b_{i_2}\cdots b_{i_r}$ that has the property that every right subword $W_j=b_{i_j} b_{i_{j+1}}\cdots b_{i_r}$ with $j>1$ is lexicographically larger than $W_1$.

The following classic theorem was discovered simultaneously in 1958 by Chen–Fox–Lyndon and Shirshov (cf. [70, 71], or [72] for a comprehensive introduction).

#### Theorem 3.5.2

Let $B=\{b_1,b_2,\ldots \}$ be an ordered set of letters and let $\textrm{Lie}[B]$ be the free Lie algebra generated by *B* (over a field which we take to be ${\mathbb {Q}}$). Then a basis of $\textrm{Lie}[B]$ is given by the individual letters $b_i$ and the set of *Lyndon brackets*

3.34 $$\begin{aligned} {[}b_{i_1}b_{i_2}\ldots b_{i_r}] , \end{aligned}$$

where the word $b_{i_1}b_{i_2}\ldots b_{i_r}$ is a Lyndon word, and the rule for making it into a Lie bracket is to place the comma at the leftmost position such that it divides the Lyndon word into two shorter Lyndon words:

3.35 $$\begin{aligned} {[}b_{i_1}b_{i_2}\ldots b_{i_r}] = \left[ [b_{i_1}\ldots b_{i_{k-1}}], [b_{i_k}\ldots b_{i_r}]\right] \end{aligned}$$

and to proceed recursively until it is a multiple bracket of single letters for which we set $[b_i] :=b_i$.

#### Examples

The first few Lyndon brackets in the free Lie algebra $\textrm{Lie}[x,y]$ are given by

3.36 $$\begin{aligned} {[}xy] = [x,y] ,\quad [xxy] = [x,[x,y]] ,\quad [xyy] = [[x,y],y] ,\quad [xxyy] = [x,[[x,y],y]]] . \end{aligned}$$

The first few Lyndon brackets in the free Lie algebra ${\mathfrak {mz}}^\vee$ on one generator $g_w$ for each odd $w\ge 3$ (see Definition 3.3.5) equipped with its Ihara Lie bracket $\{\cdot ,\cdot \}$ from (3.14) are given by

3.37 $$\begin{aligned} \{g_3g_5\} = \{g_3,g_5\} ,\ \ \{g_3g_7\} = \{g_3,g_7\} ,\ \ \{g_3g_3g_5\} = \{g_3,\{g_3,g_5\}\} . \end{aligned}$$

#### Definition 3.5.3

Since ${\mathfrak {mz}}^\vee$ is freely generated by the canonical Lie polynomials $g_3,g_5,\ldots$, the Lyndon brackets in these generators form a basis. Every such Lyndon bracket corresponds as above to a Lyndon word $g_{v_1}\cdots g_{v_r}$ with $r>1$. We write the corresponding Lyndon bracket as

3.38 $$\begin{aligned} L_{v_1 v_2 \cdots v_r} :=\{ g_{v_1}g_{v_2} \cdots g_{v_r} \}\in {\mathfrak {mz}}^\vee \end{aligned}$$

with odd $v_1,\ldots ,v_r \ge 3$. For example, $L_{335}$ denotes the Lyndon bracket $\{g_3,\{g_3,g_5\}\}$. We denote the Z-map images of the Lyndon bracket by

3.39 $$\begin{aligned} Z_{v_1\cdots v_r} :=Z(L_{v_1\cdots v_r}) , \end{aligned}$$

consistently with (3.31). These elements with $v_1+\cdots +v_r=w$ form the *semi-canonical basis* for the canonical subspace of weight *w* non-single irreducibles $I_w\subset {\mathcal{M}\mathcal{Z}}_w$.

Our next task is to establish a basis for the spaces $R_w$.

#### Proposition 3.5.4

Let $C_w\subset {\mathcal{M}\mathcal{Z}}$ be the set consisting of $\zeta _2$, the $\zeta _v$ for odd $3\le v<w$, and the Z-map images $Z_{v_1\cdots v_r}$ of Lyndon brackets $L_{v_1\cdots v_r}\in {\mathfrak {mz}}^\vee$ with $r>1$, $v_1{+}\cdots {+}v_r<w$. Then, the set of weight *w* products of elements of $C_w$ forms a linearly independent set. If *w* is odd (resp. even) all of these products (resp. all of these products except for $(\zeta _2)^{w/2}$) form a basis for $R_w$.

This proposition follows from the general result on Hopf algebras given in the following theorem (see Corollary 3.5.8). It seems like this result should be well-known, however it appears to have only been written down in an unpublished note by Perrin and Viennot [73].

#### Theorem 3.5.5

Let *X* denote an alphabet of weighted letters having the property that the number of letters in each weight is finite. Let $A^\vee$ denote the graded associative ${\mathbb {Q}}$-algebra on *X*, considered as a Hopf algebra equipped with a multiplication denoted $\diamond$ and the standard coproduct $\Delta _s$ for which the letters of *X* are primitive. Let *A* denote the graded dual space of $A^\vee$, let $L^\vee \subset A^\vee$ denote the subspace of primitive elements for $\Delta _s$, and let $B=\{b_1,b_2,\ldots \}$ be a vector space basis for $L^\vee$. Then,

(i) $L^\vee$ forms a Lie algebra whose bracket is given by $[g,h]=g\diamond h-h\diamond g$.
(ii) Both *A* and $A^\vee$ have bases given by the monomials *w* in the letters of *X*, which we denote by $w\in A$ and $w^\vee \in A^\vee$. The map $w^\vee \mapsto w$ provides an isomorphism of graded vector spaces from $A^\vee$ to *A*. As a ${\mathbb {Q}}$-algebra, however, *A* is commutative, equipped with the shuffle multiplication.
(iii) Let $\xi _i$ denote the images of the elements $b_i\in A^\vee$ under the isomorphism in (ii). The $\xi _i$ then form a multiplicative set of generators for *A* under the shuffle multiplication.
(iv) The ordered monomials  with $i_1\le i_2\le \ldots \le i_m$ form a linear basis for *A*; those with $m>1$ form a basis for the subspace $S\subset A$ annihilating $L^\vee$.

#### Proof

(i) follows directly from the Milnor–Moore theorem [74]. The vector space part of (ii) follows from the fact that each graded part is finite-dimensional, so has a dual that is isomorphic to it and equipped with a dual basis; the notation $w^\vee$ for the basis of $A^\vee$ simply defines a dual basis to the basis of monomials $w\in A$. The fact that the multiplication on *A* is the shuffle is standard, corresponding to the fact that an element of $A^\vee$ is a Lie element if and only if it satisfies the shuffle relations (see (3.7)), completing the proof of (ii). This is the same as saying that the subspace $S\subset A$ spanned by all shuffles of monomials is the subspace that annihilates the Lie algebra $L^\vee$. For this reason, the quotient space $L=A/S$ is the Lie coalgebra dual to $L^\vee$, and the linear isomorphism in (ii) induces a linear isomorphism between *L* and $L^\vee$. Hence, the $\xi _i\in A$ form a basis for a subspace ${\tilde{L}}\subset A$ isomorphic to *L*, restricted to which the quotient map $A\rightarrow A/S = L$ is an isomorphism. Thus we have $A = S\oplus {\tilde{L}}$, completing the proof of (iii).

The final point (iv) follows from the Poincaré–Birkhoff–Witt theorem [75], which states that the universal enveloping algebra of a Lie algebra is generated by the ordered monomials in elements of a basis, and the only relations come from relations in the Lie algebra. We consider $L=A/S$ as a Lie algebra with the trivial bracket, so that the only multiplicative relations between the generators $\xi _i$ of *L* are given by the fact that they commute. By the Poincaré–Birkhoff–Witt theorem, the ordered monomials  with $m\ge 1$ then form a basis for the universal enveloping algebra *A* of *L*, and the monomials with $m>1$ form a basis for the kernel of the map $A\rightarrow L$, so in fact they form a basis for *S*, proving (iv). $\square$

#### Remark 3.5.6

Essentially what this proof expresses is that the usual basis of the free associative algebra $A^\vee$ on the alphabet *X*, given by the monomials in the letters of *X*, can be replaced by a different basis consisting of the basis $b_i$ of Lie elements on the one hand, spanning the Lie algebra $L^\vee \subset A^\vee$, completed by the space $S^\vee$ spanned by shuffles of monomials on the other, so that $A^\vee =L^\vee \oplus S^\vee$. In the dual space *A*, this corresponds to an equivalent decomposition $A=L\oplus S$ where *L* is the subspace whose basis is the $\xi _i$ and *S* is the subspace spanned by all non-trivial shuffles of the $\xi _i$, which are in fact linearly independent by (iv).

#### Corollary 3.5.7

Let $A^\vee ={\overline{{\mathcal{M}\mathcal{Z}}}}^\vee$, which by Brown’s theorem [2] is freely generated by $g_3,g_5,\ldots$ under the $\diamond$ multiplication. Then the elements $Z(g_w)$ for odd $w\ge 3$ together with the shuffles3.40(called *ordered* shuffle products) form a basis for ${\overline{{\mathcal{M}\mathcal{Z}}}}=A$; in particular the ordered shuffles are linearly independent.

We now pass from ${\overline{{\mathcal{M}\mathcal{Z}}}}$ to ${\mathcal{M}\mathcal{Z}}$ by using the isomorphism (2.41).

#### Corollary 3.5.8

Let $g_3,g_5,\ldots$ denote the canonical generators of ${\mathfrak {mz}}^\vee$. Then a basis for ${\mathcal{M}\mathcal{Z}}$ is given by the following elements:

(i) the single motivic zeta values $\zeta _w$ for $w\ge 2$;
(ii) the Z-map images $Z_{w_1\cdots w_r}$ of the basis of ${\mathfrak {mz}}^\vee$ given by the Lyndon brackets $L_{w_1\cdots w_r}$ with $r>1$ of the canonical generators $g_3,g_5,\ldots$; the weight $w=w_1{+}\ldots {+}w_r$ elements of this type give a basis of $I_w$;
(iii) the ordered shuffle products of all the basis elements in (i) and (ii) above, excluding the products of even single zetas (since these products are equal to rational multiples of powers of $\zeta _2$); the weight *w* elements of this type form a basis for $R_w$.

#### Proof

A basis of ${\mathbb {Q}}[\zeta _2]$ is given by the powers of $\zeta _2$, so by (2.53) the single zeta values $\zeta _w$ for all even $w\ge 2$ also give a basis. A basis for ${\overline{{\mathcal{M}\mathcal{Z}}}}$ is given in Corollary 3.5.7. Thanks to (2.41), a basis for the tensor product is given by the products of the basis elements of each of the two vector spaces, which is precisely as described by (i), (ii) and (iii) of the statement. $\square$

### Canonical polynomials from the Drinfeld associator

In this section we introduce the Drinfeld associator [21, 22] which offers an alternative method of computing the canonical polynomials $g_w$. The Drinfeld associator is given by the power series [23]

3.41 $$\begin{aligned} \Phi _{\textrm{KZ}}(x,y) :=\textbf{1}+\sum _w (-1)^{d(w)}\zeta (w)w\in {{\mathcal {Z}}} \otimes _{\mathbb {Q}}{\mathbb {Q}}\langle \langle x,y \rangle \rangle , \end{aligned}$$

where ${\mathbb {Q}}\langle \langle x,y\rangle \rangle$ denotes the degree completion of the polynomial ring ${\mathbb {Q}}\langle x,y\rangle$, the sum runs over non-trivial monomials *w* in *x* and *y*, and for each such *w*, *d*(*w*) denotes the depth of the monomial, i.e. the number of *y*’s contained in it.^6^ Removing the signs in front of each term produces a power series that we call the *modified Drinfeld associator*, given by^7^

3.42 $$\begin{aligned} \Phi (x,y) :=\Phi _{\textrm{KZ}}(x,-y)=\textbf{1}+\sum _w \zeta (w)w\in {\mathcal {Z}}\, {\hat{\otimes }} \, {\mathcal {Z}}^\vee , \end{aligned}$$

where ${\hat{\otimes }}$ denotes the completed tensor product (allowing infinite sums). We also have formal and motivic versions

3.43 $$\begin{aligned} \Phi ^{\mathfrak {f}}\in {\mathcal{F}\mathcal{Z}}\, {\hat{\otimes }} \, {\mathcal{F}\mathcal{Z}}^\vee \ \ \ \textrm{and}\ \ \ \Phi ^{\mathfrak {m}}\in {\mathcal{M}\mathcal{Z}}\, {\hat{\otimes }}\, {\mathcal{M}\mathcal{Z}}^\vee , \end{aligned}$$

obtained by replacing $\zeta (w)$ by $\zeta ^{\mathfrak {f}}(w)$ and $\zeta ^{\mathfrak {m}}(w)$, respectively. The coefficients of all three power series $\Phi$, $\Phi ^{\mathfrak {f}}$ and $\Phi ^{\mathfrak {m}}$ satisfy the regularized double shuffle relations.

#### Definition 3.6.1

Let $V=\bigoplus _w V_w$ be a graded vector space for which each graded part is finite-dimensional, and let $V^\vee$ denote the graded dual (the direct sum of the duals of the graded parts of *V*). Choose any basis $e_1,e_2,\ldots$ for *V* respecting the grading decomposition, and let $e_1^\vee ,e_2^\vee ,\ldots$ denote the dual basis of $V^\vee$, with $\langle e_i^\vee ,e_j\rangle =\delta _{ij}$. Let

3.44 $$\begin{aligned} \Psi =\sum _{i=1}^\infty e_i\otimes e_i^\vee \in V\, {\hat{\otimes }}\, V^\vee . \end{aligned}$$

We call $\Psi$ the *canonical element* of $V\, {\hat{\otimes }} \, V^\vee$.

Note that the element $\Psi$ is independent of the choice of basis of *V* due to the use of dual bases.

#### Proposition 3.6.2

Let *V* be as in Definition 3.6.1 and let $\phi :V\rightarrow W$ denote any surjective linear morphism and $\phi ^\vee :W^\vee \rightarrow V^\vee$ denote the dual morphism. Let $\Psi$ be the canonical element of $V\,{\hat{\otimes }}\, V^\vee$. Then $\bigl (\phi \otimes (\phi ^\vee )^{-1}\bigr )(\Psi )$ (in the sense specified in the proof) is the canonical element of $W\,{\hat{\otimes }} \,W^\vee$.

#### Proof

We may assume that *V* is finite-dimensional by working with a fixed graded piece. Since $\phi$ is surjective, we have that $V/\textrm{Ker}\,\phi \cong W$. Choose a basis of *V* adapted to this quotient, i.e. linearly independent elements ${\tilde{w}}_1,\ldots , {\tilde{w}}_m\in V$ that get mapped to a basis $\{w_i=\phi ({\tilde{w}}_i)\}$ of *W* under $\phi$ and a basis $k_1,\ldots ,k_n$ of $\textrm{Ker}\,\phi$. Write the canonical element $\Psi$ in this basis:

3.45 $$\begin{aligned} \Psi =\sum _{i=1}^m {{\tilde{w}}}_i\otimes {{\tilde{w}}}_i^\vee +\sum _{j=1}^n k_j\otimes k_j^\vee . \end{aligned}$$

We now apply the map $\phi \otimes (\phi ^\vee )^{-1}$ to $\Psi$, with the understanding that this map is interpreted as the composition

3.46 $$\begin{aligned} \bigl (\textrm{id}\otimes (\phi ^\vee )^{-1}\bigr )\circ (\phi \otimes \textrm{id}\bigr ) , \end{aligned}$$

which avoids appearing to apply $(\phi ^\vee )^{-1}$ to elements not in $\phi ^\vee (W^\vee )$. We thus obtain

3.47 $$\begin{aligned} \bigl (\phi \otimes (\phi ^\vee )^{-1}\bigr )(\Psi )=\sum _{i=1}^m w_i\otimes (\phi ^\vee )^{-1}({{\tilde{w}}}_i^\vee )=\sum _{i=1}^m w_i\otimes w_i^\vee , \end{aligned}$$

which is the canonical element of $W\otimes W^\vee$. $\square$

Recall from diagram (3.25) that ${\mathbb {Q}}[Z(w)]$ is the graded dual of the power series ring ${\mathbb {Q}}\langle \langle x,y\rangle \rangle$. Then, the element

3.48 $$\begin{aligned} \Phi ^Z = \textbf{1}+\sum _w Z(w) \otimes w\in {\mathbb {Q}}[Z(w)]\, {\hat{\otimes _{\mathbb {Q}}}} \, {\mathbb {Q}}\langle \langle x,y\rangle \rangle \end{aligned}$$

is the canonical element of the tensor product ${\mathbb {Q}}[Z(w)] \, {\hat{\otimes _{\mathbb {Q}}}} \,{\mathbb {Q}}\langle \langle x,y\rangle \rangle$. Since ${\mathcal {Z}},{\mathcal{F}\mathcal{Z}}$ and ${\mathcal{M}\mathcal{Z}},$ are all quotients of ${\mathbb {Q}}[Z(w)]$ (see diagram (3.25)), Proposition 3.6.2 then implies that $\Phi$, $\Phi ^{\mathfrak {f}}$ and $\Phi ^{\mathfrak {m}}$ are the canonical elements for the respective rings ${\mathcal {Z}}\,{\hat{\otimes }}\, {\mathcal {Z}}^\vee$, ${\mathcal{F}\mathcal{Z}}\,{\hat{\otimes }}\, {\mathcal{F}\mathcal{Z}}^\vee$ and ${\mathcal{M}\mathcal{Z}}\,{\hat{\otimes }}\, {\mathcal{M}\mathcal{Z}}^\vee$. In particular, the choice of basis in which to express $\Phi ^{\mathfrak {m}}$ is of little significance in general. However, writing $\Phi ^{\mathfrak {m}}$ in the semi-canonical basis does have one convenient advantage: it provides another method to compute the canonical polynomials $g_w$.

In our semi-canonical basis of ${\mathcal{M}\mathcal{Z}}$ (see (3.31) for $Z_{35}, Z_{37}$ and $Z_{335}$), the expansion of the modified Drinfeld associator $\Phi$ to weight 11 reads as follows, see [62] for the analogous expansion of the Drinfeld associator and its significance for the motivic coaction^8^

3.49 $$\begin{aligned} \Phi&=\textbf{1}+\zeta _2 g_2+\zeta _3 g_3+\zeta _4 g_4+\zeta _5 g_5+\zeta _2\zeta _3 g_3\diamond g_2 +\zeta _6 g_6+\tfrac{1}{2}\zeta _3^2g_3\diamond g_3+\zeta _7g_7 \nonumber \\&\quad +\zeta _3\zeta _4g_3\diamond g_4+\zeta _2\zeta _5g_5\diamond g_2 +\zeta _8g_8 +\zeta _2\zeta _3^2\bigl (\tfrac{1}{2}g_3\diamond g_3\diamond g_2+\tfrac{17}{247}\{g_3,g_5\}\bigr )\nonumber \\&\quad +\tfrac{1}{24453}Z_{35}\{g_3,g_5\} +\zeta _3\zeta _5\Bigl (\tfrac{47}{114}g_3\diamond g_5+\tfrac{67}{114}g_5\diamond g_3\Bigr )\nonumber \\&\quad + \zeta _9g_9+\tfrac{1}{6}\zeta _3^3g_3\diamond g_3\diamond g_3 +\zeta _2\zeta _7g_7\diamond g_2+\zeta _4\zeta _5g_5\diamond g_4+\zeta _6\zeta _3g_3\diamond g_6\nonumber \\&\ \ \ +\zeta _{10}g_{10} +\tfrac{8}{6614309}Z_{37}\{g_3,g_7\}+\zeta _3\zeta _7\bigl (\tfrac{24581}{59858}g_3\diamond g_7+\tfrac{35277}{59858}g_7\diamond g_3\bigr )\nonumber \\&\quad +\zeta _5^2\bigl (\tfrac{1}{2}g_5\diamond g_5-\tfrac{2160}{29929}\{g_3,g_7\}\bigr )+\zeta _2Z_{35}\bigl (\tfrac{1016}{243951279}\{g_3,g_7\}+\tfrac{1}{24453}\{g_3,g_5\}\diamond g_2\bigr )\nonumber \\&\quad +\zeta _2\zeta _3\zeta _5\bigl (\tfrac{47}{114}g_3\diamond g_5\diamond g_2+\tfrac{67}{114}g_5\diamond g_3\diamond g_2+\tfrac{492798}{9667067}\{g_3,g_7\}\bigr )\nonumber \\&\quad +\zeta _4\zeta _3^2\bigl (\tfrac{85}{494}\{g_3,g_5\}\diamond g_2 +\tfrac{60072829}{502687484} \{g_3,g_7\}+\tfrac{1}{2}g_3\diamond g_3\diamond g_4\bigr )\nonumber \\&\quad +\zeta _{11}g_{11} +\tfrac{1}{3683808}Z_{335}\{g_3,\{g_3,g_5\}\}\nonumber \\&\quad +\zeta _3Z_{35}\bigl (\tfrac{7063}{625556646}g_3\diamond g_3\diamond g_5+\tfrac{5728}{312778323}g_3\diamond g_5\diamond g_3-\tfrac{6173}{208518882}g_5\diamond g_3\diamond g_3\bigr )\nonumber \\&\quad +\zeta _3^2\zeta _5\bigl (\tfrac{5439455}{46661568}g_3\diamond g_3\diamond g_5+\tfrac{4179377}{23330784}g_3\diamond g_5\diamond g_3+\tfrac{3177525}{15553856}g_5\diamond g_3\diamond g_3\bigr )\nonumber \\&\quad +\zeta _2\zeta _9\bigl (-\tfrac{31943}{22102848}\{g_3, \{g_3,g_5\}\}+g_9\diamond g_2\bigr ) +\zeta _4\zeta _7 \bigl (\tfrac{46765}{3274496} \{g_3,\{g_3,g_5\}\}+g_7\diamond g_4\bigr )\nonumber \\&\quad +\zeta _2\zeta _3^3\bigl ( \tfrac{3066359}{75825048} g_3\diamond g_3\diamond g_5-\tfrac{456995}{37912524} g_3\diamond g_5\diamond g_3\nonumber \\&\quad -\tfrac{ 2152369}{75825048} g_5\diamond g_3\diamond g_3+\tfrac{1}{6}g_3\diamond g_3\diamond g_3\diamond g_2\bigr )\nonumber \\&\quad +\zeta _8\zeta _3\bigl ( -\tfrac{1953356831}{87350455296} \{g_3, \{ g_3, g_5 \}\} +g_3\diamond g_8\bigr ) \nonumber \\&\quad +\zeta _6\zeta _5\bigl ( \tfrac{540685}{14735232} \{g_3,\{g_3,g_5\}\}+ g_5\diamond g_6\bigr ) +\ldots \end{aligned}$$

$$\square $$

#### Computational remarks

In order to write motivic MZVs in a given basis in weight *w* we need to know the linear relations between motivic MZVs in that weight. While these are not known in general, we have several possible approaches: (i) in weights up to $w=22$ (and also at weight $w=23$ modulo a 31-bit prime), it is known by dimension arguments that ${\mathcal{M}\mathcal{Z}}_w={\mathcal{F}\mathcal{Z}}_w$ [76] so we can use the double shuffle relations, (ii) since Brown gave the dimension of ${\mathcal{M}\mathcal{Z}}_w$ in all weights, if we reached any weight where ${\mathcal{M}\mathcal{Z}}_w$ is not equal to ${\mathcal{F}\mathcal{Z}}_w$ (in spite of the conjecture that they are equal) we could write the real MZVs as real numbers, seek for enough linear relations between them with rational coefficients to reach the correct dimension and then prove that these relations are motivic [76]. In practice, the latter method has been used to create the available datamines, making the decomposition particularly easy by computer as it is enough to enter an MZV into the datamine to automatically obtain its decomposition. Note that the ${\mathbb {Q}}$-bases of [76] were extended from weight 22 to weight 34 in the HyperlogProcedures of Schnetz [77].

(1) In computing the expression (3.49), we have written multiple $\diamond$-products without parentheses with the understanding that we can evaluate them as $g_{w_1}\diamond (g_{w_2}\diamond \cdots (g_{w_{r-1}}\diamond (g_{w_r}\diamond g_k))\cdots )$ with $w_i$ odd and *k* odd or even. In this way, the left factor of each $\diamond$ multiplication is a Lie polynomial, i.e. a $g_w$ with *w* odd, which allows us to use the simplified expression (3.17) for the multiplication $\diamond$ in ${\mathcal{M}\mathcal{Z}}^\vee$.

(2) This gives us three ways to recursively compute the $g_w$, of which we saw the first two earlier:

(i) from the properties in Lemma 3.3.2 that uniquely characterize the $g_w$,
(ii) get the semi-canonical basis for $I_w$ using the Lyndon words and then compute the unique normalized polynomial $g_w\in {\mathcal{M}\mathcal{Z}}_w^\vee$ annihilating the basis elements of $R_w$ and $I_w$, or
(iii) decompose $\Phi$ into the semi-canonical basis of ${\mathcal{M}\mathcal{Z}}$; then
  3.50 $$\begin{aligned} g_w = \Phi |_{\zeta _w} \end{aligned}$$

The equivalence of the third approach with the others is a direct consequence of Proposition 3.6.2, which implies that the polynomial appearing in $\Phi$ with coefficient $\zeta _w$ must be the element of the dual basis of the semi-canonical basis taking the value 1 on $\zeta _w$ and annihilating $I_w$ and $R_w$.

(3) As an advantage of the first method (i) over methods (ii) and (iii), the conditions of Lemma 3.3.2 make it clear that the canonical $g_w$ do not depend on any basis choice for ${\mathcal{M}\mathcal{Z}}_w$. For those weights *w* where the expansion of the Drinfeld associator is available (e.g. from [76, 77]), the third approach (iii) enjoys the computational advantage that ansätze and solutions of linear equation systems can be bypassed.

## The Canonical Morphism from Motivic MZVs to the *f*-Alphabet

In [2, 4], Brown proved a remarkable theorem showing that the motivic MZV Hopf algebra comodule ${\mathcal{M}\mathcal{Z}}$ is isomorphic to a certain Hopf algebra comodule ${\mathcal {F}}$ with a particularly simple structure that we recall below. However, Brown did not display a canonical isomorphism, but rather showed the existence and described the construction of a family of isomorphisms $\rho _{\vec {c}\,}: {\mathcal{M}\mathcal{Z}}\rightarrow {\mathcal {F}}$ parametrized by free rational parameters $\vec {c}$ associated to a chosen basis of non-single irreducible motivic MZVs. The goal of this section is to use the canonical polynomials $g_w$ of Definition 3.3.1 to fix a canonical choice of isomorphism

4.1 $$\begin{aligned} \rho :{\mathcal{M}\mathcal{Z}}\rightarrow {{\mathcal {F}}} . \end{aligned}$$

As in Sect. 3.4, we will allow ourselves to simplify the notation by writing $\zeta$ instead of $\zeta ^{\mathfrak {m}}$ throughout the present section, which will deal uniquely with motivic MZVs. Furthermore, in order for this section to remain coherent with the literature (see footnote 3 above) we will consider ${\mathcal{M}\mathcal{Z}}$ as a Hopf algebra comodule with the structure conferred on it by the choice of coaction $\Delta _{GB}$ and not $\Delta ^{GB}$ (see (2.32) and (2.37)). This change also modifies the structure of the dual Hopf algebra ${\mathcal{M}\mathcal{Z}}^\vee$, which instead of being equipped with the multiplication $\diamond$ satisfying (3.18), becomes equipped with the multiplication $\bullet$ defined by

4.2 $$\begin{aligned} h\bullet g :=g\diamond h , \end{aligned}$$

satisfying

4.3 $$\begin{aligned} \langle \Delta _{GB}(\xi ),g\otimes h\rangle =\langle \xi ,g\bullet h\rangle \end{aligned}$$

for all $\xi \in {\mathcal{M}\mathcal{Z}}$, $g,h\in {\mathcal{M}\mathcal{Z}}^\vee$. Moreover, the simple expression (3.17) for $g\diamond h$ in case of $g\in {\mathfrak {ds}}$ translates into

4.4 $$\begin{aligned} h\bullet g = gh + D_g(h) \end{aligned}$$

with the Ihara derivation $D_g$ defined by (3.14). The Lie subspace of ${\mathcal{M}\mathcal{Z}}^\vee$ is then equipped with the Lie bracket associated to $\bullet$, defined by

4.5 $$\begin{aligned} {[}\![g,h]\!] :=g\bullet h-h\bullet g . \end{aligned}$$

(Note that this Lie bracket satisfies $[\![g,h]\!] =-\{g,h\}$ in relation to the Ihara bracket (3.14).)

### Definition of the *f*-alphabet

We begin by defining the Hopf algebra comodule ${{\mathcal {F}}}$, familiarly called the *f*-alphabet [2, 4]. To start with, let $\overline{{\mathcal {F}}}^\vee :={\mathbb {Q}}\langle f^\vee _3,f^\vee _5,\ldots \rangle$ be the free associative Hopf algebra on one non-commutative indeterminate $f^\vee _w$ in each odd weight $w\ge 3$, with the usual (concatenation) multiplication and the standard coproduct defined by

4.6 $$\begin{aligned} \Delta _s(f_w^\vee )=f^\vee _w\otimes 1+1\otimes f^\vee _w \end{aligned}$$

for all odd $w\ge 3$. The subspace of Lie polynomials ${{\mathcal {L}}}^\vee :=\textrm{Lie}[f^\vee _3,f^\vee _5,\ldots ] \subset \overline{\mathcal{F}}^\vee$ is the space of primitive elements $f^\vee \in \overline{{\mathcal {F}}}^\vee$, i.e. elements satisfying

4.7 $$\begin{aligned} \Delta _s(f^\vee )=f^\vee \otimes 1+1\otimes f^\vee . \end{aligned}$$

Now let $\overline{{\mathcal {F}}}$ denote the Hopf algebra dual to $\overline{{\mathcal {F}}}^\vee$. The underlying vector space of $\overline{{\mathcal {F}}}$ is isomorphic to that of ${\mathbb {Q}}\langle f_3,f_5, \ldots \rangle$, the free associative algebra spanned by all monomials $f_{i_1}\cdots f_{i_r}$ in the free non-commutative indeterminates $f_i$ for odd $i\ge 3$; these monomials form a dual basis to the basis of monomials $f_{i_1}^\vee \cdots f_{i_r}^\vee$ of $\overline{{\mathcal {F}}}^\vee$ in the sense that $\langle f_{i_1}^\vee \cdots f_{i_r}^\vee , f_{j_1}\cdots f_{j_r}\rangle \!= \delta _{i_1,j_1}\cdots \delta _{i_r,j_r}$. The Hopf algebra structure of $\overline{{\mathcal {F}}}$ is given by equipping $\overline{{\mathcal {F}}}$ with the (commutative) shuffle multiplication on the monomials $f_{i_1}\cdots f_{i_r}$ and the *deconcatenation coproduct* $\Delta$ defined by

4.8 $$\begin{aligned} \Delta (f_{i_1}\cdots f_{i_r})=\sum _{j=0}^r f_{i_1}\cdots f_{i_j}\otimes f_{i_{j+1}}\cdots f_{i_r} . \end{aligned}$$

Following Brown, let us now define the comodule ${{\mathcal {F}}}$ to be the tensor product

4.9 $$\begin{aligned} {{\mathcal {F}}} :={\mathbb {Q}}[f_2]\otimes _{\mathbb {Q}}\overline{{\mathcal {F}}} , \end{aligned}$$

where $f_2$ is a new commutative indeterminate of weight 2 and the factor ${\mathbb {Q}}[f_2]$ denotes the polynomial ring over ${\mathbb {Q}}$ in the single indeterminate $f_2$. The algebra structure of $\overline{{\mathcal {F}}}$ extends to ${{\mathcal {F}}}$ by letting $f_2$ commute with $\overline{\mathcal{F}}$; the general rule is4.10for odd $i_1,\ldots ,i_r,j_1,\ldots ,j_s\ge 3$. By a slight abuse of terminology, we continue to call this product on all of ${{\mathcal {F}}}$ the *shuffle product* on ${{\mathcal {F}}}$.

The ${\mathbb {Q}}$-algebra ${{\mathcal {F}}}$ is made into a $\overline{\mathcal{F}}$-comodule by defining a coaction

4.11 $$\begin{aligned} \Delta :{{\mathcal {F}}}\rightarrow {{\mathcal {F}}}\otimes \overline{{\mathcal {F}}} \end{aligned}$$

on ${{\mathcal {F}}}$ by (4.8) above together with

4.12 $$\begin{aligned} \Delta (f_2)=f_2\otimes 1 . \end{aligned}$$

Thus the general formula for this coaction is given by

4.13 $$\begin{aligned} \Delta (f_2^n f_{i_1} f_{i_2} \ldots f_{i_r}) = \sum _{j=0}^r f_2^n f_{i_1} \ldots f_{i_j} \otimes f_{i_{j+1}} \ldots f_{i_r} \end{aligned}$$

with integer $n,r \ge 0$ and odd $i_1,\ldots ,i_r \ge 3$.

Now let ${{\mathcal {F}}}^\vee$ denote the dual of ${{\mathcal {F}}}$. The underlying vector space of ${{\mathcal {F}}}^\vee$ is a tensor product of two vector spaces

4.14 $$\begin{aligned} \langle f^\vee _2,f^\vee _4,\ldots \rangle \otimes _{\mathbb {Q}}\overline{\mathcal{F}}^\vee , \end{aligned}$$

where $\overline{{\mathcal {F}}}^\vee$ is as defined at the beginning of this section, and the left-hand factor denotes the vector space (not ring) dual of ${\mathbb {Q}}[f_2]$, with basis $f^\vee _{2n}\in {{\mathcal {F}}}^\vee$ satisfying

4.15 $$\begin{aligned} \langle f^\vee _{2n},f_2^m\rangle =\delta _{m,n} \frac{\zeta _2^n}{\zeta _{2n}} . \end{aligned}$$

By analogy with Definition 2.2.1 we set

4.16 $$\begin{aligned} f_{2m} :=\frac{ \zeta _{2m}}{\zeta _2^m} f_2^m\in {{\mathcal {F}}} \, , \end{aligned}$$

so that

4.17 $$\begin{aligned} \langle f^\vee _{2m},f_{2n}\rangle =\delta _{m,n} . \end{aligned}$$

The fact that ${{\mathcal {F}}}$ is a Hopf algebra comodule and not a Hopf algebra is reflected in the dual space by the fact that $\mathcal{F}^\vee$ is not a Hopf algebra but a Hopf algebra module over the Hopf algebra $\overline{{\mathcal {F}}}^\vee$. Thus, the concatenation multiplication does not extend from the subspace $\overline{\mathcal{F}}^\vee$ to all of ${{\mathcal {F}}}^\vee$; instead we only have an action of $\overline{{\mathcal {F}}}^\vee$ on ${{\mathcal {F}}}^\vee$, which we write as

4.18 $$\begin{aligned} a(f^\vee _{2n}b)=f^\vee _{2n}ab\in {{\mathcal {F}}}^\vee \end{aligned}$$

for $n\ge 1$ and $a,b\in \overline{{\mathcal {F}}}^\vee$. This action can be considered as a multiplication of an element of the space ${\mathbb {Q}}[f^\vee _2,f^\vee _4,\ldots ]$ with an element of $\overline{\mathcal{F}}^\vee$, but the $f^\vee _{2n}$ cannot be multiplied together. Thus every element of ${{\mathcal {F}}}^\vee$ is a sum of monomials which can be written uniquely in the form $f^\vee _{2n}b$ for some $n\ge 0$ (with the convention $f_0^\vee =1$) and some $b\in \overline{{\mathcal {F}}}^\vee$.

### A canonical choice of normalized isomorphism from ${\mathcal{M}\mathcal{Z}}$ to ${{\mathcal {F}}}$

#### Definition 4.2.1

A morphism $\phi : {\mathcal{M}\mathcal{Z}}\rightarrow {\mathcal {F}}$ is a *normalized morphism* if the following conditions hold [2, 4]:

(i) normalization: $\phi \big ( \zeta _n\big ) = f_n$ for all $n \ge 2$, where $f_{n}$ for even values $n=2m$ was defined in (4.16).
(ii) compatibility with the shuffle multiplication (4.10) on ${{\mathcal {F}}}$, 4.19
(iii) compatibility with coactions $\Delta$ in (4.13) and $\Delta _{GB}$ in (2.47), given by the following formula for all monomials *w* in *x* and *y*:
  4.20 $$\begin{aligned} \Delta \phi \big ( \zeta (w)\big ) = \phi \big ( \Delta _{GB} \zeta (w)\big ) . \end{aligned}$$
  It is understood that $\phi$ acts on each factor of the tensor product, with an additional projection from ${{\mathcal {F}}}$ to $\overline{{\mathcal {F}}}$ in the second factor, meaning that each term involving a power of $f_2$ in the second factor will be projected to zero.

#### Remark 4.2.2

The third property (4.20) translates the Goncharov–Brown coaction $\Delta _{GB}$, which is expressed by the complicated procedure given in Definition 2.1.4, into the considerably simpler deconcatenation coaction (4.13) in the *f*-alphabet.

The results summarized in the next theorem follow directly from the results of Brown in [2, 4] that we state here in a version adapted to the semi-canonical basis of Definition 3.5.3.

#### Theorem 4.2.3

(Brown). Let $w\ge 2$, let ${\mathcal{M}\mathcal{Z}}_w={\mathbb {Q}}\zeta _w\oplus I_w\oplus R_w$ be the canonical decomposition of Definition 3.3.1 and choose the semi-canonical basis of $I_w$ expressed via Lyndon words $Z_{v_1\ldots v_r}$ with odd $v_1,\ldots ,v_r \ge 3$ introduced in Definition 3.5.3. Let $\vec {c}=\{ c_{v_1\ldots v_r}\}$ denote an infinite family of rational parameters indexed by the same Lyndon words. Then for any choice of rational values for the parameters $\vec {c}$, there exists a normalized Hopf algebra comodule isomorphism

4.21 $$\begin{aligned} \rho _{\vec {c}\,}:{\mathcal{M}\mathcal{Z}}\rightarrow {\mathcal {F}}. \end{aligned}$$

Furthermore, any normalized Hopf algebra comodule isomorphism in the sense of Definition 4.2.1 corresponds to a specific choice of rational values of the parameters in $\vec {c}$.

#### Remark 4.2.4

We have used our choice of semi-canonical basis to state Brown’s theorem, but the result is in fact independent of the choice of basis and even of the choice of subspace $I_w$ of non-single irreducibles. For any such choice of $I_w$ equipped with any basis, we can use that basis to index a set of rational numbers $\vec {c}$ parametrizing the inequivalent normalized isomorphisms from ${\mathcal{M}\mathcal{Z}}$ to the *f*-alphabet, with the same constructive proof as the one indicated below for our particular choice.

Essentially, the proof of this result comes down to actually constructing the isomorphisms ${\mathcal{M}\mathcal{Z}}\rightarrow {{\mathcal {F}}}$ inductively weight by weight [2, 4]. We sketch the procedure here and work it out explicitly for small weights.

We saw in Sect. 3.5 that for weights $w\le 7$ we have $I_w=\{0\}$. Thus for these weights the theorem says that the normalized isomorphism is uniquely fixed up to $w\le 7$; it is in fact determined solely by properties (i) and (ii) of Definition 4.2.1. For $w=2,3,4$, we must have

4.22 $$\begin{aligned} \rho _{\vec {c}\,}:{\mathcal{M}\mathcal{Z}}_w&\rightarrow {{\mathcal {F}}}_w , \nonumber \\ \zeta _w&\mapsto f_w , \end{aligned}$$

since the weight spaces ${\mathcal{M}\mathcal{Z}}_w$ are 1-dimensional for these values. For weight 5, ${\mathcal{M}\mathcal{Z}}_5$ is 2-dimensional spanned by $\zeta _5$ and $\zeta _2\zeta _3$, so by (i) and (ii) we have

4.23 $$\begin{aligned} \rho _{\vec {c}\,}: {\mathcal{M}\mathcal{Z}}_5&\rightarrow {{\mathcal {F}}}_5 , \nonumber \\ \zeta _5&\mapsto f_5 ,\nonumber \\ \zeta _2\zeta _3&\mapsto f_2f_3 . \end{aligned}$$

For weight 6, ${\mathcal{M}\mathcal{Z}}_6$ is 2-dimensional spanned by $\zeta _6=\frac{35}{8}\zeta _2^3$ and $\zeta _3^2$, so all $\rho _{\vec {c}\,}$ are given by4.24Finally, in weight 7, ${\mathcal{M}\mathcal{Z}}_7$ is 3-dimensional, spanned by $\zeta _7$, $\zeta _2\zeta _5$ and $\zeta _4\zeta _3$, so we have

4.25 $$\begin{aligned} \rho _{\vec {c}\,}:{\mathcal{M}\mathcal{Z}}_7&\rightarrow {{\mathcal {F}}}_7 , \nonumber \\ \zeta _7&\mapsto f_7 , \nonumber \\ \zeta _2\zeta _5&\mapsto f_2f_5 , \nonumber \\ \zeta _4\zeta _3&\mapsto f_4f_3 . \end{aligned}$$

Starting from weight $w=8$, the presence of non-trivial spaces of non-single irreducibles $I_w\subset {\mathcal{M}\mathcal{Z}}_w$ requires additional input from the coaction property (4.20) in (iii).

#### Example

Let us illustrate this for the case of weight $w=8$, where we use the element $Z_{35}$ defined in (3.31) appearing in our semi-canonical basis constructed in Sect. 3.5. The image under $\rho _{\vec {c}\,}$ of this element is not fixed by (i) and (ii) alone, so we make the most general ansatz

4.26 $$\begin{aligned} \rho _{\vec {c}\,}(Z_{35}) = a_1 f_3 f_5 + a_2 f_5f_3 + a_3 f_2 f_3 f_3 + c_{35} f_8 \end{aligned}$$

with rational parameters $a_i,c_{35}$ and then impose (iii). By combining (3.31) and (2.49) we find that

4.27 $$\begin{aligned} \Delta _{GB}(Z_{35}) = Z_{35}\otimes 1 + 1\otimes Z_{35} - \tfrac{20163}{2} \zeta _3\otimes \zeta _5 + \tfrac{28743}{2} \zeta _5\otimes \zeta _3 -3366 \zeta _2\zeta _3\otimes \zeta _3 , \end{aligned}$$

whose $\rho _{\vec {c}\,}$-image is

4.28 $$\begin{aligned} \rho _{\vec {c}\,}\big (\Delta _{GB}(Z_{35})\big ) =\,&\rho _{\vec {c}\,}(Z_{35}) \otimes 1 + 1\otimes \rho _{\vec {c}\,}(Z_{35}) \nonumber \\&- \tfrac{20163}{2} f_3\otimes f_5 + \tfrac{28743}{2} f_5\otimes f_3 -3366 f_2f_3\otimes f_3 . \end{aligned}$$

To impose (iii) we have to compare this with the deconcatenation coaction (4.13) applied to the ansatz (4.26), which is

4.29 $$\begin{aligned} \Delta \big (\rho _{\vec {c}\,}(Z_{35})\big )&= \rho _{\vec {c}\,}(Z_{35}) \otimes 1 + 1\otimes \rho _{\vec {c}\,}(Z_{35}) + a_1 f_3\otimes f_5 + a_2 f_5 \otimes f_3 + a_3 f_2f_3\otimes f_3 . \end{aligned}$$

Comparing coefficients fixes the parameters $a_i$ but leaves $c_{35}$ undetermined, so for (4.26) we obtain

4.30 $$\begin{aligned} \rho _{\vec {c}\,}(Z_{35}) =-\tfrac{20163}{2}f_3f_5+\tfrac{28743}{2}f_5f_3-3366f_2f_3^2+c_{35}f_8\, .\end{aligned}$$

This is the first appearance of a rational parameter of $\vec {c}$ from Theorem 4.2.3. Analogous free parameters appear as the coefficient of $f_w$ in the image under $\rho _{\vec {c}\,}$ of each basis element of $I_w$. In the semi-canonical basis the parameter $c_{v_1\ldots v_r}$ corresponds to the coefficient of $f_{v_1+\ldots +v_r}$ in $\rho _{\vec {c}\,}(Z_{v_1\ldots v_r})$.

#### Definition 4.2.5

For $w\ge 8$, let ${\mathcal{M}\mathcal{Z}}_w={\mathbb {Q}}\zeta _w\oplus I_w\oplus R_w$ denote the canonical decomposition constructed in Sect. 3.3. Let $\rho _{\vec {c}}$ be the family of normalized Hopf algebra comodule isomorphisms established in the semi-canonical basis as in Theorem 4.2.3 such that its rational parameters $\vec {c}=\{ c_{v_1\ldots v_r}\}$ are indexed by Lyndon words with odd $v_1,\ldots ,v_r\ge 3$. Then we define the *canonical f-alphabet isomorphism*

4.31 $$\begin{aligned} \rho :{\mathcal{M}\mathcal{Z}}\rightarrow {{\mathcal {F}}} \quad \text {by} \quad \rho :=\rho _{\vec {0}} . \end{aligned}$$

The definition of the canonical isomorphism implies immediately

4.32 $$\begin{aligned} \rho (Z_{v_1\ldots v_r})|_{f_{w}}=0 \end{aligned}$$

for all $v_1{+}\ldots {+}v_r=w\ge 8$ (with $r>1$), which is an alternative unique characterization of $\rho$. This leads for instance to

4.33 $$\begin{aligned} \rho (Z_{35})&=-\tfrac{20163}{2}f_3f_5+\tfrac{28743}{2}f_5f_3-3366f_2f_3f_3 ,\nonumber \\ \rho (Z_{37})&=-\tfrac{5432401}{16}f_3f_7+\tfrac{7796217}{16}f_7f_3 +119340 f_5 f_5 -\tfrac{2698111}{16}f_4f_3 f_3\nonumber \\&\quad -\tfrac{29731}{4}f_2f_3f_5-\tfrac{366535}{4}f_2f_5f_3 ,\nonumber \\ \rho (Z_{335})&= 1629441 f_5f_3f_3 -1037295 f_3f_5f_3 -20223 f_3f_3f_5 +\tfrac{31943}{6} f_2 f_9\nonumber \\&\quad -473832 f_2 f_3 f_3 f_3- \tfrac{420885}{8} f_4 f_7 - \tfrac{540685}{4} f_6 f_5 +\tfrac{1953356831}{23712} f_8 f_3 . \end{aligned}$$

#### Proposition 4.2.6

The isomorphism $\rho$ is uniquely characterized by the property:

4.34 $$\begin{aligned} \rho (\xi )|_{f_w}=0 \ \text {for all} \ \xi \in I_w . \end{aligned}$$

Equivalently, one can characterize $\rho$ as the unique isomorphism ${\mathcal{M}\mathcal{Z}}\rightarrow {{\mathcal {F}}}$ that preserves the relation (3.50) between the canonical polynomial $g_w$ and the modified Drinfeld associator $\Phi$, i.e.

4.35 $$\begin{aligned} \rho (\Phi ) |_{f_w} = g_w . \end{aligned}$$

#### Proof

Since the $Z_{v_1\ldots v_r}$ at $v_1{+}\ldots {+}v_r=w$ with $r>1$ form a basis of $I_w$, we also have from (4.32) for all $w\ge 2$ that $\rho (\xi )|_{f_w}=0$ for any $\xi \in I_w$. Therefore, writing $\Phi$ in the semi-canonical basis, no irreducible MZV can contribute to the coefficient of $f_w$ in $\rho (\Phi )$ and the property (3.50) is preserved.

Note that even though the semi-canonical basis appears when defining $\rho =\rho _{\vec {0}}$ in (4.31), $\rho$ is characterized by the property (4.34) which refers only to the *canonical* subspace $I_w$ and therefore $\rho$ can be defined canonically in this way. $\square$

#### Remark 4.2.7

We end this section with a brief observation about the specific MZVs $\zeta _{3,5}$, $\zeta _{3,7}$ and $\zeta _{3,3,5}$, that are widely used in the physics literature as a basis for a non-canonical choice of (1-dimensional) subspace of non-single irreducibles in $I_w\subset {\mathcal{M}\mathcal{Z}}_w$ for $w=8,10,11$. Using (3.31) and (4.33), the canonical parameter choice $c_{35}=c_{37}=c_{335}=0$ translates into the *f*-alphabet images

4.36 $$\begin{aligned} \rho ( \zeta _{3,5})&= - 5 f_3 f_5 + \tfrac{100471}{35568} f_8 , \nonumber \\ \rho ( \zeta _{3,7})&= - 14 f_3 f_7 - 6 f_5 f_5 + \tfrac{408872741707}{40214998720} f_{10} , \nonumber \\ \rho ( \zeta _{3,3,5})&= - 5 f_3 f_3 f_5 - 45 f_2 f_9 - 3f_4 f_7 + \tfrac{5}{2}f_6 f_5 + \tfrac{1119631493}{14735232} f_{11} \end{aligned}$$

for these elements. The analogous $\rho$-images of all irreducible higher-depth motivic MZVs of weights $\le 17$ in the basis choice of [76] can be found in the ancillary files of [12].

## Canonical Zeta Generators $\sigma _w$ in Genus One

In this section we show how the canonical polynomials $g_w$ associated with zeta generators in genus zero as defined in Sect. 3.3 induce canonical zeta generators $\sigma _w$ in genus one. The construction also includes a canonical split of $\sigma _w$ into an arithmetic and a geometric part.

### The Tsunogai derivations $\epsilon _k$

In this section we write $\textrm{Lie}[a,b]$ for the fundamental Lie algebra associated to a once-punctured torus. This is a free Lia algebra on two generators and thus isomorphic to $\textrm{Lie}[x,y]$, but we prefer to distinguish the letters used because the topological fundamental group of a thrice-punctured sphere maps non-trivially to that of a once-punctured torus when two of the holes are joined together. We also have a natural map between the pro-unipotent fundamental groups, which gives a natural but highly non-trivial Lie algebra morphism

5.1 $$\begin{aligned} \textrm{Lie}[x,y]\rightarrow \textrm{Lie}[a,b] \end{aligned}$$

between the associated graded Lie algebras (see (5.29) below and “7.4.4”).

We write $\textrm{Der}^0\textrm{Lie}[a,b]$ for the subspace of Lie algebra derivations of $\textrm{Lie}[a,b]$ which annihilate the bracket $[a,b]=ab-ba$, where the last expression is valued in ${\mathbb {Q}}\langle a,b\rangle$. A derivation in $\textrm{Der}^0\textrm{Lie}[a,b]$ is entirely determined by its value on *a* (see for example Thm. 2.1 of [78] giving an explicit formula for the value of such a derivation on *b*).

#### Definition 5.1.1

Let $\delta \in \textrm{Der}^0\textrm{Lie}[a,b]$. We say that $\delta$ is of *homogeneous degree* *n* if $\delta (a)$ (and thus also $\delta (b)$) is a Lie polynomial of homogeneous degree $n+1$, i.e. if $\delta$ adds *n* to the degree of any polynomial it acts on. We furthermore assign *a*-*degree* *k* *and* *b*-*degree* $\ell$ to $\delta$ if $\delta (a)$ is a Lie polynomial of homogeneous degree $k+1$ in *a* and $\ell$ in *b*, in which case $\delta (b)$ is necessarily of *a*-degree *k* and *b*-degree $\ell +1$ (unless it vanishes). The *b*-degree of a derivation and the homogeneous *b*-degree of a polynomial in *a*, *b* is also referred to as the *depth*. The (homogeneous) degree of $\delta$ is equal to the sum of its *a*- and its *b*-degree.

We now need to introduce the *Tsunogai derivations* which were introduced by Tsunogai in 1995 [79], also see [80].

#### Definition 5.1.2

For all $i\ge 0$, let $\epsilon _{2i}$ denote the derivation of $\textrm{Lie}[a,b]$ defined by

5.2 $$\begin{aligned} \epsilon _{2i}(a)=\textrm{ad}_a^{2i}(b) ,\ \ \ \ \epsilon _{2i}([a,b])=0 \, ,\ \ \ \ i\ge 0 . \end{aligned}$$

These two conditions determine $\epsilon _{2i}$ completely: its action on *b* is given explicitly by

5.3 $$\begin{aligned} \epsilon _0(b) =0 \quad \text {and}\quad \epsilon _{2i}(b) = \sum _{j=0}^{i-1} (-1)^j \left[ \textrm{ad}_a^j(b), \textrm{ad}_a^{2i-1-j}(b) \right] ,\ \ \ \ i\ge 1 . \end{aligned}$$

We write $\mathfrak {u}$ for the Lie algebra of derivations of $\textrm{Lie}[a,b]$ generated by the $\epsilon _{2i}$ for $i\ge 0$; the $\epsilon _{2i}$ are also called *geometric derivations*.

The Lie algebra $\mathfrak {u}$ of geometric derivations $\epsilon _{2i}$ has a rich history dating back to pioneering work of Ihara [81], with detailed studies in the work of Tsunogai [79, 80]. They have become ubiquitous in the theory of elliptic MZVs as reviewed in “A.3”, see for example [26, 27, 82–85] and [32], with numerous references in the recent mathematics and string-theory literature. The derivations $\epsilon _0$ and $\epsilon _2$ defined in (5.2) play a special role. The derivation $\epsilon _0$ is nilpotent on the $\epsilon _k$ (with even $k\ge 2$) in the sense that $\textrm{ad}_{\epsilon _0}^{k-1} (\epsilon _k) =0$, see part (i) of Lemma 5.1.5 below. The derivation $\epsilon _2$ is central in $\textrm{Der}^0\textrm{Lie}[a,b]$ and will play no role in our construction.

We will also make essential use of the following $\mathfrak {sl}_2$-subalgebra of $\textrm{Der}^0\textrm{Lie}[a,b]$:

#### Definition 5.1.3

Define derivations $\epsilon _0^\vee , \textrm{h}\in \textrm{Der}^0\textrm{Lie}[a,b]$ by

5.4 $$\begin{aligned} \epsilon _0^\vee (a) = 0 ,\ \ \ \ \epsilon _0^\vee (b) = a ,\ \ \ \ \textrm{h}= [\epsilon _0,\epsilon _0^\vee ] . \end{aligned}$$

The derivations $\epsilon _0$, $\epsilon _0^\vee$ and $\textrm{h}$ generate the Lie subalgebra of $\textrm{Der}^0\textrm{Lie}[a,b]$ denoted $\mathfrak {sl}_2$. The generator $\textrm{h}$ satisfies $\textrm{h}(a) = -a$ and $\textrm{h}(b)=b$. We refer to vectors that are annihilated by $\epsilon _0$ as *highest-weight vectors* and vectors that are annihilated by $\epsilon _0^\vee$ as *lowest-weight vectors*, respectively.

#### Definition 5.1.4

We will also need to introduce the *switch* operator $\theta$, which can be considered as the automorphism of ${\mathbb {Q}}\langle \langle a,b\rangle \rangle$ that exchanges *a* and *b*, mapping a polynomial $f=f(a,b)$ to $\theta (f)$ with $[\theta (f)](a,b)=f(b,a)$, but also acts on derivations $\delta$ of ${\mathbb {Q}}\langle a,b\rangle$ by conjugation via the formula

5.5 $$\begin{aligned} \theta (\delta ):=\theta \circ \delta \circ \theta ^{-1} , \end{aligned}$$

i.e.

5.6 $$\begin{aligned} {[}\theta (\delta )](a)=\theta \bigl (\delta (b)\bigr ) ,\ \ \ \ [\theta (\delta )](b)=\theta \bigl (\delta (a)\bigr ) . \end{aligned}$$

Notice that $\theta (\epsilon _0)=\epsilon _0^\vee$ and therefore $\theta (\textrm{h})=-\textrm{h}$.

The interplay of the derivations $\epsilon _k$ with the $\mathfrak {sl}_2$-algebra and the switch operation $\theta$ in the previous definitions is reviewed in the following lemma (see for instance [27, 32, 79]).

#### Lemma 5.1.5

For even values $k\ge 2$ and even or odd $j\ge 0$, set

5.7 $$\begin{aligned} \epsilon _k^{(j)} :=\textrm{ad}_{\epsilon _0}^j(\epsilon _k) \end{aligned}$$

including $\epsilon _k^{(0)}=\epsilon _k$. Then the $\epsilon _k^{(j)}$ for $k\ge 2$ together with the generators $\epsilon _0,\epsilon _0^\vee ,\textrm{h}$ of the $\mathfrak {sl}_2$ in Definition 5.1.3 satisfy the following properties:

(i) The derivation $\epsilon _k^{(j)}$ is of *a*-degree $k-j-1$ and *b*-degree $j+1$ for $0\le j\le k-2$ (in other words, $\epsilon _k^{(j)}(a)$ is a polynomial of homogeneous *a*-degree $k-j$ and *b*-degree $j+1$) and thus of homogeneous degree *k*. We have the nilpotency property
  5.8 $$\begin{aligned} \epsilon _k^{(j)}=0 \quad \forall \ j>k-2 . \end{aligned}$$
  The $\epsilon _k^{(k-2)}$ at maximum value of *j* are highest-weight vectors of the $\mathfrak {sl}_2$.
(ii) The derivations $\epsilon _k$ with $k\ge 2$ commute with $\epsilon _0^\vee$:
  5.9 $$\begin{aligned} {[}\epsilon _0^\vee , \epsilon _k ] = 0 \quad \forall \ k\ge 2 , \end{aligned}$$
  i.e. they furnish lowest-weight vectors of $\mathfrak {sl}_2$.
(iii) The generator $\textrm{h}$ of $\mathfrak {sl}_2$ satisfies the following commutation relations:
  5.10 $$\begin{aligned} {[}\textrm{h}, \epsilon _k ] = (2-k) \epsilon _k \quad \forall \ k\ge 0 , \ \ \ \ {[}\textrm{h}, \epsilon _0^\vee ] = -2 \epsilon _0^\vee . \end{aligned}$$
  In particular this implies that the $\epsilon _k^{(j)}$ are all eigenvectors for $\textrm{h}$, with eigenvalues given by
  5.11 $$\begin{aligned} {[}\textrm{h}, \epsilon _k^{(j)} ] = (2+2j-k) \epsilon _k^{(j)} \quad \forall \ k\ge 2 , \ 0\le j \le k-2 . \end{aligned}$$
(iv) The commutation relations of the $\mathfrak {sl}_2$ generators with $\epsilon _k^{(j)}$ at $k\ge 2$ and $0\le j \le k-2$ are $[\epsilon _0, \epsilon _k^{(j)}]= \epsilon _k^{(j+1)}$ by definition, $[\textrm{h}, \epsilon _k^{(j)} ] = (2j+2-k)\epsilon _k^{(j)}$ by the previous point and
  5.12 $$\begin{aligned} {[}\epsilon _0^\vee , \epsilon _k^{(j)} ]&= j (k-1-j)\epsilon _k^{(j-1)} . \end{aligned}$$
(v) The switch operator in Definition 5.1.4 acts on the $\epsilon _k^{(j)}$ with $k\ge 2$ and $0\le j \le k-2$ via
  5.13 $$\begin{aligned} \theta \bigl (\epsilon _k^{(j)}\bigr )&=-\frac{j!}{(k-2-j)!}\epsilon _k^{(k-2-j)} . \end{aligned}$$

#### Proof

(i) The derivation $\epsilon _k$ is of *a*-degree $k-1$ and *b*-degree 1 by definition, and each application of $\textrm{ad}_{\epsilon _0}$ increases the *b*-degree by 1 without changing the total degree, so it decreases the *a*-degree by 1, proving the first statement. For the second statement, it is enough to show that $\epsilon _k^{(k-1)}=0$ even though since $\epsilon ^{(j)}_k$ shifts the (*a*, *b*) degrees of any polynomial in *a*, *b* by $(k-1-j,1+j)$, the case $\epsilon ^{(k-1)}_k$ of interest has (*a*, *b*) degrees (0, *k*) as a derivation, meaning that a priori $\epsilon _k^{(k-1)}(a)$ could be a polynomial of *a*-degree 1 and *b*-degree *k*. Since the only Lie polynomial with these degrees is $\textrm{ad}_b^k(a)$ up to scalar multiple, we must have

5.14 $$\begin{aligned} \epsilon _k^{(k-1)}(a)=c\cdot \textrm{ad}_b^k(a) \end{aligned}$$

for some constant *c*, and $\epsilon _k^{(k-1)}(b)=0$. However, the derivation $\epsilon ^{(k-1)}_k$ must annihilate the commutator [*a*, *b*] since both $\epsilon _k$ and $\epsilon _0$ do, so by the above, we have $\epsilon ^{(k-1)}_k([a,b])= c\cdot [ \textrm{ad}_b^k(a),b]$ which only vanishes for $c=0$. Thus $c=0$, so the derivation $\epsilon _k^{(k-1)}=0$.

(ii) is readily established by evaluating $[\epsilon _0^\vee , \epsilon _{2i}]=\epsilon _0^\vee \epsilon _{2i} - \epsilon _{2i} \epsilon _0^\vee$ on *a* and *b*. The least straightforward part of the computation is to note that $\epsilon _0^\vee \sum _{j=0}^{i-1}(-1)^j [\textrm{ad}_a^j(b), \textrm{ad}_a^{2i-1-j}(b)]$ receives a single contribution from the $j=0$ term, resulting in $[\epsilon _0^\vee (b), \textrm{ad}_a^{2i-1}(b)]= \epsilon _{2i}(a)$.

(iii) Any monomial in *a*, *b* is an eigenvector for $\textrm{h}$, with the difference of the *b*-degree minus the *a*-degree as its eigenvalue. Since $\epsilon _k$ at $k\ge 0$ and $\epsilon _0^\vee$ shift the (*a*, *b*)-degrees by $(k-1,1)$ and $(1,-1)$, respectively, the associated differences “*b*-degree minus *a*-degree” are shifted by $2-k$ in case of $\epsilon _k$ and $-2$ in case of $\epsilon _0^\vee$. This implies both identities in (5.10) as eigenvalue equations. The second claim (5.11) is a corollary which can for instance be inferred from $\epsilon _k^{(j)}$ shifting the (*a*, *b*)-degrees by $(k-1-j,j+1)$.

(iv) One can conveniently prove (5.12) by induction in *j*, starting with $[\epsilon _0^\vee ,\epsilon _k^{(0)}]=0$ as a base case which follows from (ii). The inductive step relies on the Jacobi identity $[\epsilon _0^\vee ,\epsilon _k^{(j)}]= [\epsilon _0^\vee ,[\epsilon _0,\epsilon _k^{(j-1)}]] =[ [\epsilon _0^\vee ,\epsilon _0],\epsilon _k^{(j-1)}] +[\epsilon _0,[\epsilon _0^\vee ,\epsilon _k^{(j-1)}]]$ as well as (5.11) to evaluate the first term $[ [\epsilon _0^\vee ,\epsilon _0],\epsilon _k^{(j-1)}]=- [ \textrm{h},\epsilon _k^{(j-1)}]$.

(v) We proceed by induction in *j*, first proving $\theta (\epsilon _k)= -\frac{1}{(k-2)!} \epsilon _k^{(k-2)}$ as a base case of (5.13) at $j=0$.

*Base case:* If a derivation of degree $>0$ annihilates the bracket [*a*, *b*], then knowing its value on one of the variables *a* or *b* determines it completely. Hence, it suffices to show that $\theta (\epsilon _k)$ and $-\frac{1}{(k-2)!} \epsilon _k^{(k-2)}$ have the same action on *b* to establish their equality as derivations in $\textrm{Der}^0\textrm{Lie}[a,b]$. For this purpose, we successively simplify

5.15 $$\begin{aligned} \epsilon _{k}^{(k-2)}(b)&= (\epsilon _0)^{k-2}\epsilon _{k}(b) =\sum _{j=0}^{\frac{k}{2}-1} (-1)^j (\epsilon _0)^{k-2} \left[ \textrm{ad}_a^j(b), \textrm{ad}_a^{k-1-j}(b) \right] \nonumber \\&= (\epsilon _0)^{k-2} \left[ b, \textrm{ad}_a^{k-1}(b) \right] = - \left[ b, (\epsilon _0)^{k-2} \textrm{ad}_a^{k-2}([b,a]) \right] \nonumber \\&=-(k-2)! \left[ b, \textrm{ad}_b^{k-2}([b,a]) \right] = -(k-2)!\,\textrm{ad}_b^{k}(a) . \end{aligned}$$

In the first step, we have used $\epsilon _0(b)=0$ to remove all contributions to $\epsilon _{k}^{(k-2)}(b)$ with an $\epsilon _0$ on the right of $\epsilon _{k}$. The second step makes use of the expression (5.3) for $\epsilon _{k}(b)$ and *k* even. The third step relies on the fact that for $m\ge 1$, $\textrm{ad}_a^m(b)$ is annihilated by $(\epsilon _0)^m$ such that $[ \textrm{ad}_a^j(b), \textrm{ad}_a^{k-1-j}(b)]$ is annihilated by $(\epsilon _0)^{k-2}$ unless $j=0$. After redistributing the $(k-1)$-fold action of $\textrm{ad}_a$ in the fourth step, we note in the fifth step that the $k-2$ factors of $\epsilon _0$ can act on the $k-2$ exposed powers of $\textrm{ad}_a$ (besides [*b*, *a*] which is annihilated by $\epsilon _0$) in $(k-2)!$ different permutations, converting $\textrm{ad}_a^{k-2}$ to $\textrm{ad}_b^{k-2}$ in all cases. The end result of (5.15) after repackaging the powers of $\textrm{ad}_b$ is equivalent to

5.16 $$\begin{aligned} \epsilon _{k}^{(k-2)}(b) = -(k-2)! \,\textrm{ad}_b^{k}(a) = -(k-2)!\, \theta \big ( \epsilon _k(a) \big ) \end{aligned}$$

by virtue of (5.2). As a consequence, $\theta (\epsilon _k)$ and $-\frac{1}{(k-2)!} \epsilon _k^{(k-2)}$ have the same action on *b* and must agree as derivations since they both annihilate [*a*, *b*] and have degree $>0$.

*Inductive step:* Now we can take care of (5.13) at values $j>0$ by induction as follows:

5.17 $$\begin{aligned} \theta (\epsilon _k^{(j)})&=\theta \big ( [\epsilon _0,\epsilon _k^{(j-1)}] \big ) = \big [\theta (\epsilon _0), \theta (\epsilon _k^{(j-1)} )\big ] =- \frac{(j-1)!}{(k-1-j)!} [ \epsilon _0^\vee , \epsilon _k^{(k-1-j)} \big ]\nonumber \\&= -\frac{(j-1)!}{(k-1-j)!} j (k-1-j)\epsilon _k^{(k-2-j)} = -\frac{j!}{(k-2-j)!} \epsilon _k^{(k-2-j)} , \end{aligned}$$

where we used $\theta (\epsilon _0) = \epsilon _0^\vee$ and the induction hypothesis $\theta (\epsilon _k^{(j-1)} ) = -\frac{(j-1)!}{(k-1-j)!} \epsilon _k^{(k-1-j)}$ in the third step and (5.12) proven as (iv) in passing to the second line. $\square$

#### Remark 5.1.6

Note that the $\epsilon _k^{(j)}$ are by no means free generators of $\mathfrak {u}$; commutators of two or more of them obey a number of relations related to period polynomials of holomorphic cusp forms on $\textrm{SL}_2(\mathbb {Z})$, the first of which were noticed by Ihara and Takao (cf. [30]). The relations between brackets of two $\epsilon _k$’s were classified in [31] where the connection with cusp forms was made explicit; subsequently Pollack in [32] unearthed many more relations, and made a general conjecture about the full set of relations between the $\epsilon _k^{(j)}$. These relations, which we call *Pollack’s relations*, were proved to be motivic in [27]. They appear in many works related to elliptic MZVs, such as for example [86] and [84], see “A.3” for a brief recap. The lowest-degree Pollack relations arise in degrees 14 and 16, and are given by

5.18 $$\begin{aligned} 0&= [\epsilon _4,\epsilon _{10}] - 3 [\epsilon _6,\epsilon _8] , \end{aligned}$$

5.19 $$\begin{aligned} 0&= 80 [\epsilon _4^{(1)}, \epsilon _{12} ] + 16 [\epsilon _{12}^{(1)},\epsilon _4] - 250 [\epsilon _6^{(1)},\epsilon _{10}] -125 [\epsilon _{10}^{(1)},\epsilon _6] + 280 [\epsilon _8^{(1)},\epsilon _8] \nonumber \\&\quad - 462[\epsilon _4, [\epsilon _4,\epsilon _8]] - 1725 [\epsilon _6,[\epsilon _6,\epsilon _4]] . \end{aligned}$$

### The genus one motivic Lie algebra

In [27], Hain and Matsumoto define a Tannakian category *MEM* of *mixed elliptic motives* and study its fundamental Lie algebra. We do not recall their construction here, but restrict ourselves to giving the main result of their article that we will use here. Let $\textrm{Lie}\,\pi _1(MEM)$ denote the graded Lie algebra associated to the unipotent radical of the fundamental group of the category *MEM*. Let $\mathfrak {sl}_2$ denotes the Lie subalgebra of $\textrm{Der}^0\textrm{Lie}[a,b]$ from Definition 5.1.3.

#### Theorem 5.2.1

(Hain–Matsumoto). There is a Lie algebra morphism (the “monodromy representation”, see section 22 of [27])

5.20 $$\begin{aligned} \textrm{Lie}\,\pi _1(MEM)\rightarrow \textrm{Der}^0\textrm{Lie}[a,b]\, \end{aligned}$$

whose image ${{\mathcal {L}}}$ is generated by the derivations $\epsilon _k^{(j)}$ for even $k>0$ and $0\le j\le k-2$ together with derivations $\sigma _w$ for each odd $w\ge 3$, and has the following properties:

(i) The Lie subalgebra ${{\mathcal {S}}} :=\textrm{Lie}[\sigma _3,\sigma _5,\ldots ]\subset {{\mathcal {L}}}$ is free,
(ii) The Lie subalgebra $\mathfrak {u}$ generated by the $\epsilon _k^{(j)}$ is normal in ${{\mathcal {L}}}$, i.e. ${{\mathcal {L}}}= \mathfrak {u} \rtimes {{\mathcal {S}}}$,
(iii) ${{\mathcal {L}}}$ is an $\mathfrak {sl}_2$-module, and $\mathfrak {u}$ is also an $\mathfrak {sl}_2$-module,
(iv) the Lie subalgebra $\mathfrak {u} \rtimes \mathfrak {sl}_2$ is normal inside ${{\mathcal {L}}} \rtimes \mathfrak {sl}_2$.

#### Remark 5.2.2

Although entirely phrased in terms of the monodromy representation of the fundamental Lie algebra of the category *MEM*, this theorem reflects essential geometric/arithmetic content. The quotient of ${{\mathcal {L}}}$ by the normal Lie subalgebra $\mathfrak {u}$ is isomorphic to ${{\mathcal {S}}}$, which is itself free on one generator in each odd rank $\ge 3$, i.e. isomorphic to $\textrm{Lie}\,\pi _1(MTM)$ the fundamental Lie algebra of the category of mixed Tate motives unramified over $\mathbb {Z}$. This in turn reflects the geometric situation where an elliptic curve parametrized by $\tau$ degenerates to the nodal elliptic curve when $\tau$ tends to $i\infty$ (see “7.4.4”).

To be more precise, if one considers the universal elliptic curve ${{\mathcal {E}}}$ as a fibration over the Deligne–Mumford compactification $\overline{{\mathcal {M}}}_{1,1}$ of the moduli space of elliptic curves ${{\mathcal {M}}}_{1,1}$ (viewed as the usual fundamental domain for the action of $\textrm{SL}_2(\mathbb {Z})$ on the Poincaré upper half-plane, parametrized by the variable $\tau$), then the fiber over $\tau =i\infty$ is the so-called nodal (or degenerate) elliptic curve $E_\infty$. Let $\pi _1$ denote the fundamental group of the punctured torus, freely generated by loops $\alpha$ and $\beta$ through and around the genus hole, and let ${\hat{\pi }}_{1}$ be its profinite completion. Then there is a canonical *arithmetic* outer Galois action of the absolute Galois group $\textrm{Gal}({\overline{{\mathbb {Q}}}}/{\mathbb {Q}})$ on ${\hat{\pi }}_1(E_\infty )$. Furthermore, since ${{\mathcal {E}}}$ is a fibration over the base ${{\mathcal {M}}}_{1,1}$ with an elliptic curve as a fiber, $\pi _1({{\mathcal {E}}})$ fits into a short exact sequence whose kernel is free on two generators (the $\pi _1$ of the fiber) and whose quotient is $\textrm{SL}_2(\mathbb {Z})$ (the $\pi _1$ of the base). Hence, there is a second, *geometric* outer group action on $\pi _1(E_\infty )$ by the group $\textrm{SL}_2(\mathbb {Z})$, which extends to an action of the profinite completion $\widehat{\textrm{SL}}_2(\mathbb {Z})$ on ${\hat{\pi }}_1(E_\infty )$. Thus we have two disjoint profinite groups, $\widehat{\textrm{SL}}_2(\mathbb {Z})$ and the absolute Galois group $\textrm{Gal}({\overline{{\mathbb {Q}}}}/{\mathbb {Q}})$ [87], acting as automorphism groups of ${\hat{\pi }}_1(E_\infty )$.

The pro-unipotent version of this situation, or rather the associated Lie algebra version, has ${{\mathcal {S}}}= \textrm{Lie}\,\pi _1(MTM)$ playing the role of $\textrm{Gal}({\overline{{\mathbb {Q}}}}/{\mathbb {Q}})$ and $\mathfrak {u}\rtimes \textrm{sl}_2$ playing the role of $\textrm{SL}_2(\mathbb {Z})$, both acting as derivation Lie algebras (the Lie algebra version of automorphism groups) of $\textrm{Lie}[a,b]$, the free Lie algebra on two generators which plays the role of ${\hat{\pi }}_1(E_\infty )$. The fact that ${{\mathcal {S}}}$ acts on $\mathfrak {u}\rtimes \mathfrak {sl}_2$ reflects the fact that $\textrm{Gal}({\overline{{\mathbb {Q}}}}/{\mathbb {Q}})$ acts not only on ${\hat{\pi }}_1(E_\infty )$ but also on $\widehat{\textrm{SL}}_2(\mathbb {Z})$, since the latter group is also a fundamental group, namely of ${{\mathcal {M}}}_{1,1}$.

Hain and Matsumoto conjecture that the surjective morphism from $\textrm{Lie}\,\pi _1(MEM)$ to ${{\mathcal {L}}}$ is actually an isomorphism, but this is still an open question. They further explain that there is a natural surjection from $\textrm{Lie}\,\pi _1(MEM)$ to $\textrm{Lie}\,\pi _1(MTM)$, the fundamental Lie algebra of the category of mixed Tate motives unramified over $\mathbb {Z}$. Since this category was shown by Brown to be generated by the motivic MZVs, we have the isomorphism

5.21 $$\begin{aligned} \textrm{Lie}\,\pi _1(MTM) = {\mathfrak {mz}}^\vee , \end{aligned}$$

where ${\mathfrak {mz}}^\vee$ is the Lie algebra associated to the motivic MZVs. Hain and Matsumoto further proved the existence of a section map

5.22 $$\begin{aligned} \textrm{Lie}\,\pi _1(MTM)\hookrightarrow \textrm{Lie}\,\pi _1(MEM) , \end{aligned}$$

which explains the semi-direct product structure in Theorem 5.2.1 (ii), with the image of $\textrm{Lie}\, \pi _1(MTM)$ identified with ${{\mathcal {S}}}\subset \textrm{Lie} \,\pi _1(MEM)$. The section map was defined explicitly in independent parallel work by Enriquez in [26], working with the *Grothendieck–Teichmüller Lie algebra* ${\mathfrak {grt}}$. Thanks to this work, ${{\mathcal {S}}}$ is identified as a canonical Lie subalgebra of ${{\mathcal {L}}}$. However, neither Hain–Matsumoto nor Enriquez gave a canonical choice of the actual generators $\sigma _w$ for odd $w\ge 3$; a priori, the choice of generator $\sigma _w$ is only defined up to adding on brackets of $\sigma _u$ with smaller $u<w$. This exactly parallels the fact that no special set of free generators of the motivic Lie algebra $\textrm{Lie}\,\pi _1(MTM) = {\mathfrak {mz}}^\vee$ was defined prior to the canonical family of $g_w$ in genus zero defined in Sect. 3.3.

Our main purpose in this section is to point out that, thanks to the canonical genus zero generators $g_w$ and the existence of the section map (5.22), we can now define a canonical choice of genus one generators $\sigma _w$ simply as the images of the $g_w$ under the section map. More precisely, we will construct an explicit Lie algebra morphism

5.23 $$\begin{aligned} {\tilde{\gamma }}:{\mathfrak {mz}}^\vee \rightarrow \textrm{Lie}[\sigma _3,\sigma _5,\ldots ,]\subset \textrm{Der}^0\textrm{Lie}[a,b] \end{aligned}$$

and use it to define the $\sigma _w$ (as images of the $g_w$), to compute them and to determine many of their properties. In the same way as the Ihara derivations of $g_w$ are called zeta generators in genus zero, we will refer to the $\sigma _w$ as *zeta generators in genus one*. The tight interplay of zeta generators in genus zero and one can for instance be seen from (5.41) below where the action of $\sigma _w$ is computed from $g_w$. Additional facets of the relation between zeta generators in genus zero and genus one can be found in “7.4.4”.

Let us show how the map ${\tilde{\gamma }}$ in (5.23) relates to the Grothendieck–Teichmüller section map defined by Enriquez. We do not need to give the definition of ${\mathfrak {grt}}$ here, but only to mention two essential properties that we need: firstly, there is an injective morphism

5.24 $$\begin{aligned} {\mathfrak {mz}}^\vee&\hookrightarrow {\mathfrak {grt}},\nonumber \\ h(x,y)&\mapsto h(x,-y) , \end{aligned}$$

(this is a direct consequence of the fact that Goncharov’s motivic MZVs satisfy the associator relations, see for example [88]) and secondly, Enriquez [26] defined an injective map

5.25 $$\begin{aligned} {\mathfrak {grt}}\hookrightarrow \textrm{Der}^0\textrm{Lie}[a,b] \end{aligned}$$

which was shown in [29] to be equivalent to the Hain–Matsumoto section, using methods from Écalle’s mould theory that will be explained in Sect. 6 below. Let

5.26 $$\begin{aligned} \gamma :{\mathfrak {mz}}^\vee \hookrightarrow \textrm{Der}^0\textrm{Lie}[a,b] \end{aligned}$$

denote the composition of (5.24) with (5.25). The explicit isomorphism ${\tilde{\gamma }}$ announced in (5.23) is given by

5.27 $$\begin{aligned} {\tilde{\gamma }}=\theta \circ \gamma , \end{aligned}$$

where $\theta$ is the switch automorphism of ${\mathbb {Q}}\langle \langle a,b\rangle \rangle$ exchanging *a* and *b*, see Definition 5.1.4.

#### Definition 5.2.3

Let $g_w$ for odd $w\ge 3$ denote the family of canonical free generators of ${\mathfrak {mz}}^\vee$ given in Definition 3.3.5. Set

5.28 $$\begin{aligned} \tau _w :=\gamma (g_w) , \ \ \ \ \sigma _w :={\tilde{\gamma }}(g_w) , \end{aligned}$$

where $\gamma$ is as in (5.26) and ${\tilde{\gamma }}$ as in (5.27). This definition accomplishes the second goal of this article of giving a canonical choice for the zeta generators $\sigma _w$ in genus one for odd $w\ge 3$.

The remainder of Sect. 5 and all of Sects. 6 and 7 are devoted to the study of the canonical zeta generators $\sigma _w$ in genus one. Section 5.3 gives an explicit step-by-step construction of the Enriquez map (5.25), and in Theorem 5.4.1 of Sect. 5.4 we list several properties of the zeta generators $\sigma _w$ and their switch images $\tau _w$. Section 5.5 contains the low-degree parts of $\sigma _w$ for $w=3,5,7,9$. The proofs of some of the properties in Theorem 5.4.1 rely on a second, mould theoretic construction of the map $\gamma$, which is given in Sect. 6.1 along with a necessary introduction to mould theory; the full proof of the theorem is contained in Sect. 6.2 (using mould theory), Sect. 6.3 (using the $\mathfrak {sl}_2$ subalgebra of Definition 5.1.3) and Sect. 7.1 (summarizing the essential argument of [27, 89]). Section 7.3 introduces a recursive procedure to compute high-degree contributions to $\sigma _w$ in terms of $\epsilon _k$ which leads to a variety of explicit results beyond the state-of-the-art in Sect. 7.4.

### Genus one derivations from genus zero polynomials

Since in this section we will work only in odd weights *w*, we can work entirely mod $\zeta _2$, in the ${\mathbb {Q}}$-algebras ${\overline{{\mathcal{F}\mathcal{Z}}}}$ and ${\overline{{\mathcal{M}\mathcal{Z}}}}$.

The surjection ${\mathcal{F}\mathcal{Z}}\rightarrow \!\!\!\!\!\rightarrow {\mathcal{M}\mathcal{Z}}$ from Sect. 2.2 induces a surjection ${\overline{{\mathcal{F}\mathcal{Z}}}}\rightarrow \!\!\!\!\!\rightarrow {\overline{{\mathcal{M}\mathcal{Z}}}}$ and a surjection ${\mathfrak {fz}}\rightarrow \!\!\!\!\!\rightarrow {\mathfrak {mz}}$. As we saw in the previous sections, we can pass to the dual spaces using the Z-map and these surjections induce injections ${\mathfrak {mz}}^\vee \hookrightarrow {\mathfrak {fz}}^\vee ={\mathfrak {ds}}$ and ${\overline{{\mathcal{M}\mathcal{Z}}}}^\vee \hookrightarrow {\overline{{\mathcal{F}\mathcal{Z}}}}^\vee ={\mathcal {U}}{\mathfrak {ds}}$ in the dual spaces. The complete situation combining all the surjections, dual inclusions and Z-maps is summarized in the diagram (3.25).

The map from $g_w$ to $\sigma _w$ is to be viewed as a map from genus zero to genus one, see “7.4.4”. The genus zero situation here is represented by the Lie algebra $\textrm{Lie}[x,y]$, which is identified with the graded Lie algebra associated to the pro-unipotent completion of the fundamental group $\pi _1$ of the sphere with three punctures (which is free on two generators). The genus one situation is represented by the completion $\widehat{\textrm{Lie}}[a,b]\subset {\mathbb {Q}}\langle \langle a,b\rangle \rangle$ of the free Lie algebra on two generators $\textrm{Lie}[a,b]$, the graded Lie algebra of the pro-unipotent fundamental group of the once-punctured torus. The topological map from the sphere to the torus obtained by joining two of the punctures passes to the topological fundamental groups, their unipotent completions and then via formality isomorphisms to the corresponding graded Lie algebras, yielding the following Lie algebra morphism:

5.29 $$\begin{aligned} \psi :\textrm{Lie}[x,y]&\rightarrow \widehat{\textrm{Lie}}[a,b] ,\nonumber \\ x&\mapsto t_{12} ,\nonumber \\ y&\mapsto t_{01} , \end{aligned}$$

where letting $\textrm{B}_n$ denote the standard Bernoulli numbers,

5.30 $$\begin{aligned} t_{01}&:=\frac{\textrm{ad}_b}{e^{\textrm{ad}_b}-1}(-a)=-a-\sum _{n\ge 1} \frac{\textrm{B}_n}{n!}\textrm{ad}_b^n(a) \nonumber \\&=-a + \tfrac{1}{2} \textrm{ad}_b(a) - \tfrac{1}{12} \textrm{ad}_b^2(a) + \tfrac{1}{720} \textrm{ad}_b^4(a) +\ldots , \nonumber \\ t_{12}&:=[a,b] . \end{aligned}$$

The map $\psi$ in (5.29) also arises when computing the Knizhnik–Zamolodchikov–Bernard connection on a degeneration limit of the torus (corresponding topologically to the degenerate torus obtained by joining two punctures of the thrice-punctured sphere), and matching the result with the Knizhnik–Zamolodchikov connection on the sphere. This calculation is spelled out in detail in “7.4.4”.

In order to explicitly define the map $\gamma$ in (5.26), we will make use of the notion of a *partner* [29]: for any $g(a,b)\in \textrm{Lie}[a,b]$, we write $g=g_aa+g_bb$ and define the partner of *g* by the formula

5.31 $$\begin{aligned} g' :=\sum _{i\ge 0} \frac{(-1)^{i-1}}{i!} a^ib\,\partial ^i_a\bigl (g_a\bigr )\in {\mathbb {Q}}\langle a,b\rangle , \end{aligned}$$

where $\partial _a$ is the derivation of ${\mathbb {Q}}\langle \langle a,b\rangle \rangle$ defined by $\partial _a(a)=1$ and $\partial _a(b)=0$. It is shown in Lemma 2.1.1 of [29] that the derivation $a\mapsto g$, $b\mapsto g'$ lies in $\textrm{Der}^0\textrm{Lie}[a,b]$ if and only if *g* has a certain property called *push-invariance* to which we will return in Sect. 6 (see (6.21)).

We can now proceed to the explicit definition of the map $\gamma$ of (5.26). Define $\tau _h :=\gamma (h)\in \textrm{Der}^0\widehat{\textrm{Lie}}[a,b]$ to be the derivation obtained from $h\in {\mathfrak {mz}}^\vee$ by the following procedure:

- Let $h=h(x,y)$ be in ${\mathfrak {mz}}^\vee$ and define a derivation $\kappa _h$ of the Lie subalgebra $\textrm{Lie}[t_{12},t_{01}]\subset \widehat{\textrm{Lie}}[a,b]$ by^9^
  5.32 $$\begin{aligned} \kappa _{h}(t_{12})=0 , \ \ \ \ \kappa _{h}(t_{01})= [t_{01},h(t_{12},-t_{01})] . \end{aligned}$$
- By the “extension lemma” 2.1.2 of [29], there exists a unique derivation $\tau _{h}$ of ${\mathbb {Q}}\langle \langle a,b\rangle \rangle$ having the following two properties: firstly
  5.33 $$\begin{aligned} \tau _{h}(t_{01})=\kappa _{h}(t_{01}) , \end{aligned}$$
  and secondly $\tau _{h}(b)$ is (in each degree) the *partner* of $\tau _{h}(a)$ as defined in (5.31).
  Specifically, the action of the derivation $\tau _h$ on *a* can be inferred from (5.33) degree by degree as follows. Suppose *h*(*x*, *y*) is homogeneous of degree *w* in *x*, *y*. We have from (5.30)
  5.34 $$\begin{aligned} \tau _h(t_{01})=\tau _h\bigl (-a+\tfrac{1}{2}[b,a]-\tfrac{1}{12}[b,[b,a]]+\cdots \bigr ) \end{aligned}$$
  so
  5.35 $$\begin{aligned} \tau _h(a)=-\kappa _h(t_{01})+\tfrac{1}{2}\tau _h\big ([b,a]\big )-\tfrac{1}{12}\tau _h\big ([b,[b,a]]\big )+\cdots \end{aligned}$$
  since $\tau _h(t_{01})=\kappa _h(t_{01})$. In particular, the lowest degree part of $\tau _h(a)$ is equal to the lowest degree part of $-\kappa _h(t_{01})$, which is equal to $[a,h^d([a,b],a)]$ from (5.32) and where *d* denotes the minimal *x*-degree of *h* and $h^d(x,y)$ are the contributions to *h*(*x*, *y*) of *x*-degree *d*; the term $[a,h^d([a,b],a)]$ is of degree $w+d+1$ in *a*, *b*. So we have
  5.36 $$\begin{aligned} \tau _h(a)_{w+d+1}=-\kappa _h(t_{01})_{w+d+1}= [a,h^d([a,b],a)] \end{aligned}$$
  in lowest degree, where $g_d$ denotes the degree-*d* contributions to polynomials *g* in *a* and *b*. We set $\tau _h(b)_{w+d+1}$ to be the partner of $\tau _h(a)_{w+d+1}$ using the formula (5.31).
  We then use (5.35) to recursively compute $\tau _h(a)$ in successive degrees $w+d+i$ ($i>1$):
  5.37 $$\begin{aligned} \tau _h(a)_{w+d+2}&=-\kappa _h(t_{01})_{w+d+2}+\tfrac{1}{2}[\tau _h(b)_{w+d+1},a]+\tfrac{1}{2}[b,\tau _h(a)_{w+d+1}] , \nonumber \\ \tau _h(a)_{w+d+3}&=-\kappa _h(t_{01})_{w+d+3}+\tfrac{1}{2}[\tau _h(b)_{w+d+2},a]+\tfrac{1}{2}[b,\tau _h(a)_{w+d+2}]\nonumber \\&\quad -\tfrac{1}{12}[\tau _h(b)_{w+d+1},[b,a]] -\tfrac{1}{12}[b,[\tau _h(b)_{w+d+2},a]]\nonumber \\&\quad -\tfrac{1}{12}[b,[b,\tau _h(a)_{w+d+1}]] , \nonumber \\ \hbox {etc.} , \end{aligned}$$
  defining $\tau _h(b)_{w+d+i}$ to be the partner of $\tau _h(a)_{w+d+i}$ at each successive degree via (5.31). This process yields a unique Lie series $\tau _h(a)$. As observed just after (5.31), if $\tau _h(a)$ has the property of *push-invariance* then $\tau _h\in \textrm{Der}^0\widehat{\textrm{Lie}}[a,b]$, so in particular $\tau _h$ annihilates $[a,b]=t_{12}$, and thus $\tau _h$ is an extension of $\kappa _h$ to all of $\textrm{Der}^0\widehat{\textrm{Lie}}[a,b]$. The fact that $\tau _h(a)$ does indeed possess the necessary property of push-invariance is proved in Theorem 6.1.6 (iii) below.
- For each $h\in {\mathfrak {mz}}^\vee$, we define $\sigma _h\in \textrm{Der}^0\widehat{\textrm{Lie}}[a,b]$ to be the derivation obtained from $\tau _h$ by the switch operator in Definition 5.1.4: we set
  5.38 $$\begin{aligned} \sigma _{h}=\theta (\tau _{h}) , \end{aligned}$$
  or equivalently, $\sigma _{h}$ acts on *a* and *b* via
  5.39 $$\begin{aligned} \sigma _{h}(a)=\theta \bigl (\tau _{h}(b)\bigr ) , \ \ \ \ \sigma _{h}(b)=\theta \bigl (\tau _{h}(a)\bigr ) . \end{aligned}$$

Combining all the steps of the process above then yields explicit versions

5.40 $$\begin{aligned} \gamma :{\mathfrak {mz}}^\vee&\hookrightarrow \textrm{Der}^0\textrm{Lie}[a,b] ,&{\tilde{\gamma }}:{\mathfrak {mz}}^\vee&\hookrightarrow \textrm{Der}^0\textrm{Lie}[a,b] \, , \nonumber \\ h&\mapsto \tau _{h} ,&h&\mapsto \sigma _{h} , \end{aligned}$$

of the maps $\gamma$ from (5.26) and ${\tilde{\gamma }}$ from (5.23).

### The canonical genus one derivations $\sigma _w$

We shall now specialize the above construction of $\gamma (h)$ and ${\tilde{\gamma }}(h)$ for general $h \in {\mathfrak {mz}}^\vee$ to the canonical polynomials $h \rightarrow g_w$ of Definition 3.3.5 for odd $w\ge 3$. The concrete realization of the maps $\gamma ,\tilde{\gamma }$ in (5.40) provided by the previous section allows for an explicit computation of the zeta generators $\sigma _w,\tau _w$ in (5.28). By (5.32) and (5.39), the action of the genus one zeta generators $\sigma _w =\tilde{\gamma }(g_w)$ on the smaller Lie subalgebra $\textrm{Lie}[t_{01},t_{12}]\subset \widehat{\textrm{Lie}}[a,b]$ is given by

5.41 $$\begin{aligned} \sigma _w(t_{12})=0 \, , \ \ \ \ \sigma _w\big ( \theta (t_{01}) \big ) = \theta \big (\big [ t_{01} , g_w(t_{12},- t_{01})\big ] \big ) \end{aligned}$$

obtained from applying the switch $\theta$ to

5.42 $$\begin{aligned} \tau _w(t_{12})=0 , \ \ \ \ \tau _w(t_{01})= \big [ t_{01} , g_w( t_{12},- t_{01})\big ] . \end{aligned}$$

By the discussion in Sect. 3.6, the canonical polynomials $g_w$ are determined by the (modified) Drinfeld associator and the ${\mathbb {Q}}$ relations among MZVs. Hence, the information from iterated integrals in genus zero already fixes the defining relations (5.42) of zeta generators in genus one. Further discussions of the tight interplay between genus zero and genus one can be found in “7.4.4”.

In the previous section, we explained how to infer $\tau _h(a)$ and $\tau _h(b)$ from $\tau _h(t_{01})$ and $\tau _h(t_{12})$ for general $h \in {\mathfrak {mz}}^\vee$ from (5.32) by the extension lemma 2.1.2 of [29]. To compute $\sigma _w(a)$ and $\sigma _w(b)$, we can either apply that method with $h=g_w$ and use the switch $\theta$ or use the same method directly from (5.41).

The derivations $\tau _w$ and $\sigma _w$ associated to $g_w$ for odd *w* have many remarkable properties, of which a number are listed in the following theorem. Several of these are statements for the different degree parts of $\tau _w$ and $\sigma _w$ (where degree refers to the degree as a derivation). The degree 2*w* parts of $\tau _w$ and $\sigma _w$ turn out to play a special role and are called the *key degree* parts $\tau ^{\textrm{key}}_w$ and $\sigma ^{\textrm{key}}_w$. In Sect. 6.1 we will present a brief introduction to mould theory which will enable us to prove the first three of these in Sect. 6.2; the others are proved in Sect. 6.3. Part (i) and (ii) of the theorem below are already known from [29] and implicitly from [26, 27]. Part (iv) follows straightforwardly from Theorem 5.2.1 [27]. Part (v) is essentially in [27], see for instance Remark 20.4. The last two sentences of part (vi) readily follow from Theorem 5.2.1 as can be seen from their proof in Sect. 6.3.2 below. Part (vii) was proven in section 27 of [27] as will be reviewed in Sect. 7.1 below.

#### Theorem 5.4.1

For odd $w\ge 3$, the zeta generators $\tau _w$ and $\sigma _w$ in Definition 5.2.3 satisfy:

(i) Both $\tau _w$ and $\sigma _w$ lie in $\textrm{Der}^0\widehat{\textrm{Lie}}[a,b]$.
(ii) The minimal degree of $\tau _w$ and $\sigma _w$ is $w+1$, and all odd-degree terms are equal to zero. All terms of the power series $\tau _w(a)$ are of constant *a*-degree $w+1$, or equivalently (thanks to the switch), all terms of the power series $\sigma _w(a)$ have constant *b*-degree *w*.
(iii) Both $\tau _w$ and $\sigma _w$ are entirely determined by their parts of degree $<2w$.
(iv) There are no highest-weight vectors of $\mathfrak {sl}_2$ in $\sigma _w$ beyond key degree.
(v) All contributions to $\tau _w$ and $\sigma _w$ of degree different from 2*w* lie in $\mathfrak {u}$. The key-degree parts $\tau ^\textrm{key}_w$ and $\sigma ^{\textrm{key}}_w$ do not lie in $\mathfrak {u}$.
(vi) Define the *arithmetic part* $z_w \in \textrm{Der}^0\widehat{\textrm{Lie}}[a,b]$ of the derivation $\sigma _w$ to be the one-dimensional component of $\sigma ^{\textrm{key}}_w$ as an $\mathfrak {sl}_2$ representation, i.e. which commutes with the generators $\epsilon _0,\epsilon _0^\vee$ of $\mathfrak {sl}_2 \subset \textrm{Der}^0 \widehat{\textrm{Lie}}[a,b]$ in Definition 5.1.3. Then, the difference $\sigma ^{\textrm{key}}_w - z_w$ and by (v) in fact all of $\sigma _w {-} z_w$ lies in $\mathfrak {u}$. Moreover, while the $z_w$ themselves do not lie in $\mathfrak {u}$, the brackets $[z_w,\epsilon _k]$ for any even $k\ge 0$ lie in $\mathfrak {u}$.
(vii) $\sigma _w$ commutes with the infinite series *N* in geometric derivations defined by
  5.43 $$\begin{aligned} N:=-\epsilon _0 + \sum _{k=2}^\infty (2k-1) \frac{\textrm{B}_{2k}}{(2k)!} \epsilon _{2k} . \end{aligned}$$

#### Remark 5.4.2

As pointed out in [10, 27], the characterization of the arithmetic parts $z_w$ in the earlier literature as commuting with $\mathfrak {sl}_2$ and not lying in $\mathfrak {u}$ does not identify the $z_w$ uniquely; ambiguities remain for $w\ge 7$, since one can modify $z_w$ by adding on $\mathfrak {sl}_2$-invariant combinations of $\epsilon _k^{(j)}$ in $\sigma ^{\textrm{key}}_w-z_w$ while keeping the overall $\sigma _w$ unchanged (see for instance Remark 20.3 (ii) of [27]). In order to eliminate this ambiguity, we added the defining property in Theorem 5.4.1 (vi) that $z_w$ exhausts the one-dimensional irreducible $\mathfrak {sl}_2$ representations of $\sigma ^{\textrm{key}}_w$ (or equivalently, $\sigma ^{\textrm{key}}_w-z_w$ contains no one-dimensional irreducible representations of $\mathfrak {sl}_2$). Moreover, the canonical zeta generators $\sigma _w$ established with the help of the polynomials $g_w(x,y)$ resolve an independent class of earlier ambiguities in $z_w$, namely it is no longer possible to add on nested brackets of lower-weight $z_v$ with $v<w$ (e.g. for example, we cannot add a multiple of $[z_3,[z_3,z_5]]$ to $z_{11}$). Hence, the properties in part (vi) of Theorem 5.4.1 single out unique canonical arithmetic derivations $z_w$ at each odd $w\ge 3$.

### Expansions of $\sigma _w$ in low degree

In this section we spell out the explicit low-degree parts of the $\sigma _w$ up to $w=9$, in order to give a feel for their appearance. For this purpose, we rewrite the expansion of $\sigma _w(a)$ and $\sigma _w(b)$ resulting from (5.41) and the extension lemma in terms of the geometric derivations $\epsilon _{k}^{(j)}$ in (5.7) acting on *a* and *b*, up to the arithmetic parts $z_w$ at key degree described in Theorem 5.4.1 (vi). Note that according to Theorem 5.4.1 (v), the “key degree” part $\sigma ^{\textrm{key}}_w$ of $\sigma _w$, which is the part in degree 2*w* (as a derivation) is the only part not consisting of brackets of $\epsilon _{k}^{(j)}$.

In Sect. 5.5.3 below we give a more detailed description of a first computation algorithm, but begin by presenting a few examples to convey an impression of the structure of the $\sigma _w$. In the following examples for $w=3,5,7$, we decompose $\sigma ^{\textrm{key}}_w$ into the unique choice of its $\mathfrak {sl}_2$ invariant part $z_w$ in Theorem 5.4.1 (vi) and nested brackets of $\epsilon _k^{(j)}$ in $(\ge 3)$-dimensional irreducible representations of $\mathfrak {sl}_2$. An alternative algorithm for the computation of $\sigma _w$—in particular how to determine the infinity of terms beyond key degree 2*w*—is described in Sect. 7.3.

#### The case $w=3$

In this situation, we first give the complete calculation of the derivations $\tau _3$ and $\sigma _3$ related by the switch, and specify the arithmetic derivation $z_3$ by directly giving its values on *a* and *b*. Recall that the switch maps $\tau _w$ to $\sigma _w$ via (5.39) and acts on the derivations $\epsilon _k^{(j)}$ according to (5.13). Direct computation based on (5.42) shows that

5.44 $$\begin{aligned} \tau _3&=\epsilon _4+\tau _3^ \textrm{key}-\tfrac{1}{960}[\epsilon _4^{(1)},\epsilon _4^{(2)}]+\tfrac{1}{725760}[\epsilon _4^{(1)},\epsilon _6^{(4)}]-\tfrac{1}{1451520}[\epsilon _4^{(2)},\epsilon _6^{(3)}] \nonumber \\&\quad +\tfrac{1}{1741824000}[\epsilon _4^{(2)},\epsilon _8^{(5)}] -\tfrac{1}{870912000}[\epsilon _4^{(1)},\epsilon _8^{(6)}] +\tfrac{1}{2786918400} [\epsilon _4^{(2)},[\epsilon _4^{(2)},\epsilon _6^{(4)}]] \nonumber \\&\quad +\tfrac{1}{1931334451200}[\epsilon _4^{(1)},\epsilon _{10}^{(8)}] -\tfrac{1}{3862668902400}[\epsilon _4^{(2)},\epsilon _{10}^{(7)}]+\ldots , \end{aligned}$$

with an infinite series in nested brackets of $\epsilon _{k_i}^{(j_i)}$ of total degree $\sum _i k_i \ge 16$ in the ellipsis. Here and in Sect. 5.5.2 below, we have made a choice on how the Pollack relations of Remark 5.1.6 are used to represent the degree $\ge 14$ terms of $\sigma _w$ and $\tau _w$.

The key-degree part $\tau _3^{\textrm{key}}$ concentrated in degree 6 is given explicitly by

5.45 $$\begin{aligned} \tau _3^{\textrm{key}}(a)&= -\tfrac{1}{4}[aaababb] -\tfrac{1}{4}[aaabbab] -\tfrac{1}{12}[aababab] ,\nonumber \\ \tau _3^\textrm{key}(b)&=\tfrac{1}{4}[aababbb]+\tfrac{1}{4}[aabbabb]+\tfrac{1}{4}[aabbbab]+\tfrac{1}{12}[abababb]\, , \end{aligned}$$

where we employ the Lyndon-bracket notation introduced in Theorem 3.5.2.

Applying the switch (5.39) and (5.13) to $\tau _3$ and $\tau _3^{\textrm{key}}$, we obtain the following explicit formula for $\sigma _3$ (again skipping an infinity of contributions at degree $\sum _i k_i \ge 16$):

5.46 $$\begin{aligned} \sigma _3&= -\tfrac{1}{2} \epsilon _4^{(2)}+z_3+\tfrac{1}{480} [\epsilon _4,\epsilon _4^{(1)}]+\tfrac{1}{30240} [\epsilon _4^{(1)},\epsilon _6] -\tfrac{1}{120960} [\epsilon _4,\epsilon _6^{(1)}]+\tfrac{1}{7257600} [\epsilon _4,\epsilon _8^{(1)}] \nonumber \\&\quad -\tfrac{1}{1209600} [\epsilon _4^{(1)},\epsilon _8] -\tfrac{1}{58060800} [\epsilon _4,[\epsilon _4,\epsilon _6]]\nonumber \\&\quad +\tfrac{1}{47900160} [\epsilon _4^{(1)},\epsilon _{10}]-\tfrac{1}{383201280} [\epsilon _4,\epsilon _{10}^{(1)}] + \ldots . \end{aligned}$$

For $w=3$ it turns out that the key-degree part $\sigma _3^\textrm{key}$ is already $\mathfrak {sl}_2$ invariant and therefore coincides with the arithmetic derivation $z_3$ whose action on *a* and *b* is given by

5.47 $$\begin{aligned} z_3(a)&=\tfrac{1}{4}[aaababb] +\tfrac{1}{4}[aaabbab] +\tfrac{1}{12}[aababab] , \nonumber \\ z_3(b)&=-\tfrac{1}{4}[aababbb] -\tfrac{1}{4}[aabbabb] -\tfrac{1}{4}[aabbbab] -\tfrac{1}{12}[abababb] . \end{aligned}$$

An exact expression for the whole of the power series $\sigma _3$ will be given as a closed formula in Sect. 7.4.3 below.

#### The case $w=5,7,9$

Now we give the lowest-degree contributions to the expansions of $\sigma _5$, $\sigma _7$ and $\sigma _9$:

5.48 $$\begin{aligned} \sigma _5&= -\tfrac{1}{24} \epsilon _6^{(4)} -\tfrac{5 }{48} [\epsilon _4^{(1)},\epsilon _4^{(2)}] +z_5 +\tfrac{1}{5760} [\epsilon _4,\epsilon _6^{(3)}] -\tfrac{1}{5760} [\epsilon _4^{(1)},\epsilon _6^{(2)}] +\tfrac{1}{5760} [\epsilon _4^{(2)},\epsilon _6^{(1)}] \nonumber \\&\quad +\tfrac{1}{3456} [\epsilon _4,[\epsilon _4,\epsilon _4^{(2)}]] +\tfrac{1}{6912} [\epsilon _4^{(1)},[\epsilon _4^{(1)},\epsilon _4]] +\tfrac{1}{145152} [\epsilon _6^{(1)},\epsilon _6^{(2)}] -\tfrac{1}{145152} [\epsilon _6,\epsilon _6^{(3)}] \nonumber \\&\quad -\tfrac{1}{2073600} [\epsilon _4,[\epsilon _4,\epsilon _6^{(2)}]] +\tfrac{139 }{72576000} [\epsilon _4^{(1)},[\epsilon _4,\epsilon _6^{(1)}]] -\tfrac{23 }{24192000} [\epsilon _4,[\epsilon _4^{(1)},\epsilon _6^{(1)}]] \nonumber \\&\quad -\tfrac{1007 }{145152000} [\epsilon _4^{(2)},[\epsilon _4,\epsilon _6]] -\tfrac{1}{4147200} [\epsilon _4^{(1)},[\epsilon _4^{(1)},\epsilon _6]] +\tfrac{289 }{48384000} [\epsilon _4,[\epsilon _4^{(2)},\epsilon _6]] \nonumber \\&\quad +\tfrac{1}{145152000} [\epsilon _6,\epsilon _8^{(3)}] -\tfrac{1}{36288000} [\epsilon _6^{(1)},\epsilon _8^{(2)}]\nonumber \\&\quad +\tfrac{1}{14515200} [\epsilon _6^{(2)},\epsilon _8^{(1)}] -\tfrac{1}{7257600} [\epsilon _6^{(3)},\epsilon _8] + \ldots \end{aligned}$$

5.49 $$\begin{aligned} \sigma _7&= -\tfrac{1}{720} \epsilon _8^{(6)} +\tfrac{7 }{1152} [\epsilon _4^{(2)},\epsilon _6^{(3)}] -\tfrac{7 }{1152} [\epsilon _4^{(1)},\epsilon _6^{(4)}] \nonumber \\&\quad -\tfrac{661 }{57600} [\epsilon _4^{(1)},[\epsilon _4^{(1)},\epsilon _4^{(2)}]] -\tfrac{661 }{57600} [\epsilon _4^{(2)},[\epsilon _4^{(2)},\epsilon _4]] \nonumber \\&\quad +\tfrac{1}{172800} [\epsilon _4,\epsilon _8^{(5)}] -\tfrac{1}{172800} [\epsilon _4^{(1)},\epsilon _8^{(4)}] +\tfrac{1}{172800} [\epsilon _4^{(2)},\epsilon _8^{(3)}]\nonumber \\&\quad +\tfrac{1}{13824} [\epsilon _6^{(1)},\epsilon _6^{(4)}] -\tfrac{1}{13824} [\epsilon _6^{(2)},\epsilon _6^{(3)}] \nonumber \\&\quad +z_7 -\tfrac{1}{4354560} [\epsilon _6,\epsilon _8^{(5)}] +\tfrac{1}{4354560} [\epsilon _6^{(1)},\epsilon _8^{(4)}]\nonumber \\&\quad -\tfrac{1}{4354560} [\epsilon _6^{(2)},\epsilon _8^{(3)}] +\tfrac{1}{4354560} [\epsilon _6^{(3)},\epsilon _8^{(2)}] \nonumber \\&\quad -\tfrac{1}{4354560} [\epsilon _6^{(4)},\epsilon _8^{(1)}] +\tfrac{7 }{552960} [\epsilon _4,[\epsilon _4,\epsilon _6^{(4)}]]\nonumber \\&\quad +\tfrac{7 }{552960} [\epsilon _4,[\epsilon _4^{(1)},\epsilon _6^{(3)}]] +\tfrac{7 }{184320} [\epsilon _4^{(1)},[\epsilon _4^{(2)},\epsilon _6^{(1)}]] \nonumber \\&\quad +\tfrac{7 }{552960} [\epsilon _4^{(2)},[\epsilon _4,\epsilon _6^{(2)}]] -\tfrac{7 }{184320}\nonumber \\&\quad [\epsilon _4,[\epsilon _4^{(2)},\epsilon _6^{(2)}]] -\tfrac{7 }{276480} [\epsilon _4^{(2)},[\epsilon _4^{(2)},\epsilon _6]] \nonumber \\&\quad -\tfrac{7 }{552960} [\epsilon _4^{(1)},[\epsilon _4,\epsilon _6^{(3)}]] -\tfrac{7 }{552960} [\epsilon _4^{(2)},[\epsilon _4^{(1)},\epsilon _6^{(1)}]] + \ldots \end{aligned}$$

5.50 $$\begin{aligned} \sigma _9&= -\tfrac{1}{40320} \epsilon _{10}^{(8)} -\tfrac{1}{5184} [\epsilon _4^{(1)},\epsilon _8^{(6)}] +\tfrac{1}{5184} [\epsilon _4^{(2)},\epsilon _8^{(5)}] -\tfrac{7 }{20736}\nonumber \\&\quad [\epsilon _6^{(3)},\epsilon _6^{(4)}] +\tfrac{1}{9676800} [\epsilon _4,\epsilon _{10}^{(7)}] \nonumber \\&\quad -\tfrac{1}{9676800} [\epsilon _4^{(1)},\epsilon _{10}^{(6)}] +\tfrac{1}{9676800} [\epsilon _4^{(2)},\epsilon _{10}^{(5)}]\nonumber \\&\quad +\tfrac{7 }{4147200} [\epsilon _6^{(1)},\epsilon _8^{(6)}] -\tfrac{7 }{4147200} [\epsilon _6^{(2)},\epsilon _8^{(5)}] \nonumber \\&\quad +\tfrac{7 }{4147200} [\epsilon _6^{(3)},\epsilon _8^{(4)}] -\tfrac{7 }{4147200} [\epsilon _6^{(4)},\epsilon _8^{(3)}] \nonumber \\&\quad -\tfrac{529 }{691200} [\epsilon _4,[\epsilon _4^{(2)},\epsilon _6^{(4)}]] +\tfrac{2959 }{2419200} [\epsilon _4^{(1)},[\epsilon _4^{(2)},\epsilon _6^{(3)}]] \nonumber \\&\quad +\tfrac{5891 }{6220800} [\epsilon _4^{(2)},[\epsilon _4,\epsilon _6^{(4)}]] -\tfrac{443 }{967680} [\epsilon _4^{(1)},[\epsilon _4^{(1)},\epsilon _6^{(4)}]]\nonumber \\&\quad -\tfrac{799 }{1088640} [\epsilon _4^{(2)},[\epsilon _4^{(2)},\epsilon _6^{(2)}]] -\tfrac{10651 }{21772800} [\epsilon _4^{(2)},[\epsilon _4^{(1)},\epsilon _6^{(3)}]] + \ldots \end{aligned}$$

In all cases, the ellipsis refers to an infinite series in nested brackets of $\epsilon _{k_i}^{(j_i)}$ of total degree $\sum _i k_i \ge 16$, and the expansion of $\sigma _9$ additionally involves an arithmetic contribution $z_9$ at key degree 18. The action of the arithmetic derivation $z_5$ on the generators *a* is given by

5.51 $$\begin{aligned} z_5(a)&=-\tfrac{[aaaaababbbb]}{240}-\tfrac{[aaaaabbbbab]}{240}+\tfrac{[aaaabaabbbb]}{120}+\tfrac{[aaaabababbb]}{80}-\tfrac{[aaaababbabb]}{30}\nonumber \\&\quad +\tfrac{[aaaababbbab]}{60} +\tfrac{[aaaabbaabbb]}{80} -\tfrac{7[aaaabbababb]}{120}-\tfrac{[aaaabbabbab]}{30} +\tfrac{[aaaabbbaabb]}{80}\nonumber \\&\quad +\tfrac{[aaaabbbabab]}{240}+\tfrac{[aaaabbbbaab]}{240} -\tfrac{[aaabaababbb]}{24}-\tfrac{3 [aaabaabbabb]}{80}-\tfrac{7[aaabaabbbab]}{240}\nonumber \\&\quad -\tfrac{[aaababaabbb]}{240}+\tfrac{73 [aaababababb]}{240}+\tfrac{49[aaabababbab]}{80}\nonumber \\&\quad +\tfrac{3[aaababbaabb]}{80}+\tfrac{149[aaababbabab]}{240}\nonumber \\&\quad +\tfrac{[aaababbbaab]}{240}-\tfrac{[aaabbaababb]}{240}-\tfrac{[aaabbaabbab]}{60}+\tfrac{[aaabbabaabb]}{240}+\tfrac{5 [aaabbababab]}{16}\nonumber \\&\quad -\tfrac{[aaabbabbaab]}{240} +\tfrac{[aaabbbaabab]}{240}+\tfrac{[aaabbbabaab]}{120}+\tfrac{[aabaabaabbb]}{240}+\tfrac{[aabaabababb]}{240}\nonumber \\&\quad -\tfrac{[aabaababbab]}{30}+\tfrac{[aabaabbaabb]}{120}-\tfrac{[aabaabbabab]}{30}-\tfrac{3 [aababaababb]}{80}-\tfrac{3[aababaabbab]}{80}\nonumber \\&\quad -\tfrac{[aabababaabb]}{240} +\tfrac{[aababababab]}{16} , \end{aligned}$$

again using the Lyndon bracket notation of Theorem 3.5.2. A similar expression for $z_5(b)$ can be reconstructed from (5.51) by virtue of the following observation:

##### Remark 5.5.1

The Lie polynomials $z_w(a)$ and $z_w(b)$ at $w=3$, $w=5$ and $w=7$ are related by the switch $\theta$ via

5.52 $$\begin{aligned} z_w(b) = - \theta \big ( z_w(a) \big ) , \ \ \ \ w\le 7 . \end{aligned}$$

Note that an alternative method for the computation of $z_3(a),z_3(b),z_5(a),z_5(b)$ was given by Pollack in [32], though the approach in that reference has not yet led to explicit results for $z_{w\ge 7}$. Machine-readable expressions for $z_w(a)$ and $z_w(b)$ at $w=3,5,7$ can be found in an ancillary file of the arXiv submission of this work.

#### Computational aspects

We close this section by giving more details on the practical implementation of Definition 5.2.3 to determine the canonical zeta generators $\sigma _w$ and their arithmetic parts $z_w$.

The starting point of the construction is to solve the conditions (5.41) degree by degree following (5.36) and (5.37) and the partner condition. We recall from Theorem 5.4.1 that at degree *d* the derivation $(\sigma _w)_d$ has *a*-degree $d{-}w$ and *b*-degree *w*.

For the example of $\sigma _3$ the extension lemma leads at lowest degree to^10^

5.53 $$\begin{aligned} \big (\sigma _3(a) \big )_{5}&= -[aabbb] +[ababb] ,\quad \big (\sigma _3(b) \big )_{5} = -[abbbb] , \end{aligned}$$

by using $g_3$ presented in (3.32) as well as (5.36). We here employ Lyndon bracket notation in the Lie algebra $\textrm{Lie}[a,b]$. From (5.37) we then obtain at the next degree (which is here already key degree):

5.54 $$\begin{aligned} \big (\sigma _3(a) \big )_{7}&= \tfrac{1}{4} [aaababb] + \tfrac{1}{4} [aaabbab] +\tfrac{1}{12} [aababab] ,\nonumber \\ \big (\sigma _3(b) \big )_{7}&= -\tfrac{1}{4} [aababbb] -\tfrac{1}{4}[aabbabb] -\tfrac{1}{4} [aabbbab] -\tfrac{1}{12} [abababb] . \end{aligned}$$

Since (5.53) is not at key degree, we know from Theorem 5.4.1 that it must be possible to rewrite it completely as the action of a geometric derivation, i.e. an element of $\mathfrak {u}$. We know moreover from part (ii) of that theorem that the *total depth*, meaning the total number of $\epsilon _i$ (for $i\ge 0$) of any term is equal to $w=3$. Together with the information on the degree, computable from Lemma 5.1.5, this leaves very few possible terms. For any nested backet of the form $\epsilon _{k_1}^{(j_1)} \cdots \epsilon _{k_r}^{(j_r)}$ (with $k_i\ge 4$ and any allowed placement of brackets) the conditions to be allowed at degree *d* in $\sigma _w$ are

5.55 $$\begin{aligned} r+\sum _{i=1}^r j_i&= w  &   \text {for the total depth and}\nonumber \\ \sum _{i=1}^r k_i&= d  &   \text {for the degree.} \end{aligned}$$

For example, for the lowest degree $d=4$ in (5.53), the only possible term in $\sigma _3$ is proportional to $\epsilon _4^{(2)}$ and the constant of proportionality $c_1$ is fixed by

5.56 $$\begin{aligned} \big (\sigma _3(a) \big )_5 = \left[ \big (c_1 \epsilon _4^{(2)} \big )(a)\right] _5 = c_1\left( 2[aabbb]-2[ababb]\right) \end{aligned}$$

to the value $c_1=-\tfrac{1}{2}$ when comparing to (5.53), in agreement with (5.46) and a general formula to be derived in Corollary 6.2.3.

The next-to-lowest degree in $\sigma _3$, given by (5.54), is the key degree $d=2w=6$ and therefore contains both the arithmetic $z_3$ part, transforming in an $\mathfrak {sl}_2$ singlet, as well as possible geometric contributions. The most general ansatz compatible with (5.55) is

5.57 $$\begin{aligned} \sigma ^{\textrm{key}}_3 = z_3 + c_2 \epsilon _6^{(2)} . \end{aligned}$$

In order to separate out the geometric from the arithmetic term, we use that $z_3$ is a singlet under $\mathfrak {sl}_2$ and thus commutes with $\epsilon _0$. The general relations

5.58 $$\begin{aligned} \epsilon _0\big (\sigma _w(a) \big ) -\sigma _w(b) = [\epsilon _0,\sigma _w](a) ,\quad \quad \epsilon _0\big (\sigma _w(b) \big )=[\epsilon _0,\sigma _w](b) \end{aligned}$$

at key degree depend only on the geometric part due to $[\epsilon _0,\sigma ^{\textrm{key}}_w] = [\epsilon _0,\sigma ^{\textrm{key}}_w-z_w]$. Moreover, the commutator $[\epsilon _0,\sigma ^{\textrm{key}}_w-z_w]$ of the geometric term can be evaluated easily according to general representation theory as in Lemma 5.1.5. The left-hand sides of the general conditions (5.58) only depend on $\sigma _w(a)$ and $\sigma _w(b)$ that are furnished by (5.41) whereas the geometric contribution on the right-hand sides can be computed using the ansatz.

In the case of (5.54) we can use the second equation of (5.58) and find for the left-hand side

5.59 $$\begin{aligned} \epsilon _0\big (\sigma ^{\textrm{key}}_3(b) \big ) = 0 \end{aligned}$$

as well as

5.60 $$\begin{aligned} c_2 \epsilon _6^{(3)}(b) = 12c_2 \big ( 2[aabbbb]+5 [ababbbb] + 2[abbabbb]\big ) \end{aligned}$$

for the right-hand side, implying $c_2=0$ and that the action of $z_3$ is given by (5.54), which agrees with the expression already presented in (5.47).

The ansätze for the degree *d* parts of $\sigma _w$ rapidly grow with *d* and *w*. For instance, the candidate terms for $(\sigma _7)_{12}$ compatible with (5.55) are given by

5.61 $$\begin{aligned} (\sigma _7)_{12}&= c_{1} \epsilon _{12}^{(6)} +c_{2} [\epsilon _4,\epsilon _8^{(5)}] +c_{3} [\epsilon _4^{(1)},\epsilon _8^{(4)}] +c_4 [\epsilon _4^{(2)},\epsilon _8^{(3)}] \nonumber \\&\quad +c_{5} [\epsilon _6^{(1)},\epsilon _6^{(4)}] +c_{6} [\epsilon _6^{(2)},\epsilon _6^{(3)}] +c_{7} [\epsilon _4^{(1)},[\epsilon _4^{(1)},\epsilon _4^{(2)}]] +c_{8} [\epsilon _4^{(2)},[\epsilon _4^{(2)},\epsilon _4]] . \end{aligned}$$

By matching the action of this ansatz on *a* with $\big (\sigma _7(a)\big )_{13}$ computed from (5.41), we find the values of the above $c_i$ noted in the degree 12 parts of (5.49) including a vanishing coefficient $c_1$ of $\epsilon _{12}^{(6)}$. The absence of terms in $\sigma _w$ with a single $\epsilon _k^{(j)}$ at any degree besides the minimal degree $w+1$ will follow from Proposition 7.3.4 (i) below.

In summary, the strategy for converting the result of the extension lemma construction of $\sigma _w$ into expressions in terms of geometric and arithmetic derivations is to make an ansatz for the geometric terms at a given degree subject to the constraints (5.55).^11^ Away from key degree, evaluating this ansatz on *a* and *b* and equating it with the explicit form of $\sigma _w$ then fixes the ansatz (modulo free parameters that are in one-to-one correspondence with the Pollack relations defining $\mathfrak {u}$). At key degree one can separate the geometric from the arithmetic part of $\sigma _w$ using (5.58) by first computing the geometric part; then the arithmetic $z_w$ is simply the difference $z_w=\sigma ^{\textrm{key}}_w-\left( \sigma ^{\textrm{key}}_w |_{\mathfrak {u}}\right)$.

In Sect. 7.3, we will provide additional calculational tools that recursively determine $\sigma _w$ up to highest-weight vectors of $\mathfrak {sl}_2$ (see Definition 5.1.3). In case of (5.61), the ansatz contains two highest-weight vectors $[\epsilon _6^{(1)},\epsilon _6^{(4)}]-[\epsilon _6^{(2)},\epsilon _6^{(3)}]$ and $[\epsilon _4^{(1)},[\epsilon _4^{(1)},\epsilon _4^{(2)}]] +[\epsilon _4^{(2)},[\epsilon _4^{(2)},\epsilon _4]]$, and the method of Sect. 7.3 can efficiently determine 6 out of the 8 parameters $c_i$. By Theorem 5.4.1 (iv), there are no highest-weight vectors in $\sigma _w$ beyond key degree. Hence, a major virtue of the method in Sect. 7.3 is that the evaluation of *infinitely* many contributions $\sigma _w(a)_{d>2w+1}$ via (5.41) can be bypassed, i.e. that the extension lemma construction of Sect. 5.3 only needs to be applied to a *finite* range of degrees where it fixes *all* terms.

## Properties of $\tau _w$ and $\sigma _w$

In this section, we prove the properties of the derivation $\tau _w,\sigma _w$ or zeta generators in genus one listed in Theorem 5.4.1 (i) to (vi). One of the key tools for parts (i)–(iii) will be Écalle’s theory of moulds developed in [28] (see also [53] for an exposition of the basic theory), and the proof of parts (iv)–(vi) will make use of the $\mathfrak {sl}_2$ algebra in Definition 5.1.3.

### Introduction to moulds

For the reader’s convenience, we first review a few basic definitions and facts about moulds, and one fundamental theorem due to Écalle (cf. [28, 53]).

#### Moulds and power series

##### Definition 6.1.1

A *rational mould* over a ring *R* is a family of rational functions $F=\bigl (F_r\bigr )_{r\ge 0}=(F_0,F_1,F_2,\ldots )$ such that

6.1 $$\begin{aligned} F_r(u_1,\ldots ,u_r)\in R(u_1,\ldots ,u_r) , \end{aligned}$$

i.e. $F_r$ is a function of *r* commutative variables $u_i$. The *constant term* of the mould $F_0$ lies in the ring *R*. We will generally refer to a rational mould simply as a “mould”, and most of the time we will work over the base field ${\mathbb {Q}}$. Also, when there is no possibility of confusion, we often write $F(u_1,\ldots ,u_r)$ instead of $F_r(u_1,\ldots ,u_r)$. The function $F_r$ or $F(u_1,\ldots ,u_r)$ is called the *depth* *r* *part of the mould* *F*. When the rational functions $F_r$ are polynomials for all $r>0$, we say that *F* is a *polynomial mould*. Moulds can be added componentwise and multiplied by a constant in *R* componentwise. The moulds with constant term 0 thus form a vector space, denoted *ARI*; its vector subspace of polynomial moulds is denoted $ARI^{pol}$. The names of the various objects, morphisms and properties are due to Écalle [28].

Let $c_i=\textrm{ad}_x^{i-1}y$ for $i\ge 1$. From now on unless otherwise stated we will work with $R={\mathbb {Q}}$. The power series in ${\mathbb {Q}}\langle \langle x,y\rangle \rangle$ that can be written as power series in the $c_i$ are exactly the ring of power series *p* satisfying $\partial _x(p)=0$, where $\partial _x$ is the derivation defined by $\partial _x(x)=1$, $\partial _x(y)=0$. These power series are in bijection with the free ring ${\mathbb {Q}}\langle \langle c_1,c_2,\ldots \rangle \rangle$ of power series on the non-commutative variables $c_i$. All Lie-like and group-like power series in ${\mathbb {Q}}\langle \langle x,y\rangle \rangle$ belong to ${\mathbb {Q}}\langle \langle c_1,c_2,\ldots \rangle \rangle$ and indeed, with the exception of the element *x*, all Lie polynomials in *x*, *y* are in bijection with the Lie polynomials in the $c_i$. There is a simple bijection between power series $p\in {\mathbb {Q}}\langle \langle c_1,c_2,\ldots \rangle \rangle$ and polynomial moulds, given by letting $p^r$ denote the part of *p* of homogeneous degree *r* in the $c_i$ (i.e. homogeneous degree *r* in *y*) and mapping $p^r$ to the space of polynomial moulds of depth *r* by the map on monomials

6.2 $$\begin{aligned} ma:c_{i_1}\dots c_{i_r}\mapsto (-1)^{r+i_1+\cdots +i_r}u_1^{i_1-1}\cdots u_r^{i_r-1} , \end{aligned}$$

extended by linearity. We often use the notation $P=ma(p)$ for the polynomial mould associated to a power series $p\in {\mathbb {Q}}\langle \langle c_1,c_2,\ldots \rangle \rangle$ under the map *ma*. The vector space of power series without constant term maps isomorphically under *ma* to the vector space $ARI^{pol}$.

#### Basic operators on moulds

The space of moulds *ARI* is equipped with many operations. All those given in the following list are natural extensions to moulds of familiar operations on power series in *x* and *y* (see [28] or [53] for complete definitions and details).

- Mould multiplication is defined by:
  6.3 $$\begin{aligned} mu(G,H)(u_1,\ldots ,u_r)=\sum _{i=0}^r G(u_1,\ldots ,u_i)H(u_{i+1},\ldots ,u_r) . \end{aligned}$$
  This multiplication is valid for moulds with non-zero constant term as well, and is compatible with power series multiplication in the sense that if $G=ma(g)$ and $H=ma(h)$ for $g,h\in {\mathbb {Q}}\langle \langle c_1,c_2,\ldots ,\rangle \rangle$, then
  6.4 $$\begin{aligned} ma\bigl (gh\bigr )=mu(G,H) . \end{aligned}$$
- The Lie bracket *lu* on *ARI* is defined by
  6.5 $$\begin{aligned} lu(G,H)=mu(G,H)-mu(H,G) , \end{aligned}$$
  and when *ARI* is considered as a Lie algebra under this bracket, it is denoted $ARI_{lu}$. Again, for $G=ma(g)$ and $H=ma(h)$ as above, we have
  6.6 $$\begin{aligned} ma\bigl ([g,h]\bigr )=lu(G,H) . \end{aligned}$$
- For each mould $G\in ARI$, there is a derivation *arit*(*G*) of the Lie algebra $ARI_{lu}$ which generalizes the Ihara derivation $D_g$ for $g\in \textrm{Lie}[x,y]$ defined by (3.15) in the sense that if $G=ma(g)$ and $H=ma(h)$ for $g,h\in \textrm{Lie}[x,y]$ then
  6.7 $$\begin{aligned} arit(G)\cdot H=-ma\bigl (D_g(h)\bigr ) . \end{aligned}$$
  (The minus sign is due to the original definition of *arit* by Écalle).
- The *ari-bracket* is another Lie bracket on the space *ARI* (besides *lu* introduced in (6.5)), defined by
  6.8 $$\begin{aligned} ari(G,H)=arit(H)\cdot G-arit(G)\cdot H+lu(G,H) . \end{aligned}$$
  The ari-bracket generalizes the Ihara bracket (3.14) on the underlying vector space $\textrm{Lie}[x,y]$ in the sense that if $G=ma(g)$ and $H=ma(h)$ for $g,h\in \textrm{Lie}[x,y]$ then
  6.9 $$\begin{aligned} ari(G,H)=ma\bigl (\{g,h\}\bigr ) . \end{aligned}$$
  We denote the Lie algebra formed by the vector space *ARI* equipped with the *ari*-bracket by $ARI_{ari}$.
- The universal enveloping algebra $\mathcal {U} ARI_{ari}$ of the Lie algebra $ARI_{ari}$ is nothing other than the space of all (rational in the context of this article) moulds; these are essentially the same moulds as in *ARI* except that arbitrary constant terms are allowed. By the Poincaré–Birkhoff–Witt theorem, this universal enveloping algebra is equipped with an associative multiplication law which we denote by $\diamond$. The expression for this multiplication $G\diamond H$ simplifies in the case where $G\in ARI$, in which situation it is given for *G* in $ARI_{ari}$ and *H* in $\mathcal {U}ARI_{ari}$ by
  6.10 $$\begin{aligned} G\diamond H=mu(G,H)-arit(G)\cdot H , \end{aligned}$$
  which thanks to (6.7) generalizes the $\diamond$ multiplication introduced in (3.17):
  6.11 $$\begin{aligned} G\diamond H=ma\bigl (g\diamond h\bigr ). \end{aligned}$$
- The *ari-exponential* map from $ARI_{ari}$ to the group-like elements in the universal enveloping algebra is defined for $F\in ARI$ by
  6.12 $$\begin{aligned} \exp _{ari}(F)=Id+\sum _{n\ge 1} \frac{1}{n!} \bigl (\underbrace{F\diamond F\diamond \ldots \diamond F}_n\bigr ) , \end{aligned}$$
  where the $\diamond$ multiplication must be applied from right to left so that the leftmost element being multiplied is always *F*, and *Id* denotes the *mu*- and $\diamond$-identity mould $(1,0,0,\ldots )$. The image of the space *ARI* under the map $\exp _{ari}$ is called *GARI*, and it consists precisely of the set of all (here rational) moulds with constant term 1. The set *GARI* forms a group with respect to the multiplication obtained from lifting the *ari* Lie bracket to *GARI* using the Baker–Campbell–Hausdorff formula. The ari-exponential has an inverse map, the *ari-logarithm*
  6.13 $$\begin{aligned} \log _{ari}:GARI\rightarrow ARI . \end{aligned}$$
- The group *GARI* acts on the Lie algebra $ARI_{ari}$ via the *adjoint action*, under which each mould $P\in GARI$ gives an isomorphism of the Lie algebra $ARI_{ari}$ via the *adjoint operator* $\textrm{Ad}_{ari}(P)$. Let $L:=\log _{ari}(P)$, so $L\in ARI$. Then the adjoint action of *P* on a mould $A\in ARI$ can be expressed and computed explicitly by the standard formula
  6.14 $$\begin{aligned} \textrm{Ad}_{ari}(P)(A)&=A+ari(L,A)+\tfrac{1}{2}ari(L,ari(L,A))\nonumber \\&\quad +\tfrac{1}{6}ari(L,ari(L,ari(L,A)))+ \cdots \end{aligned}$$
  by exponentiating the *ari* bracket $ari(L,\cdot )$.
- We define an operator *dur* acting on all moulds by $dur(F)(\emptyset )=F(\emptyset )$ and the following formula for $r\ge 1$:
  6.15 $$\begin{aligned} dur(F)(u_1,\ldots ,u_r)=(u_1+\cdots +u_r)F(u_1,\ldots ,u_r) . \end{aligned}$$
  If $F=ma(f)$ for a power series $f\in {\mathbb {Q}}\langle \langle c_1,c_2,\ldots \rangle \rangle$ (considered as a function *f*(*x*, *y*)), then
  6.16 $$\begin{aligned} dur(F)=ma([x,f]) . \end{aligned}$$
- We will also need the mould operator $\Delta$ defined by $\Delta (F)(\emptyset )=F(\emptyset )$ and
  6.17 $$\begin{aligned} \Delta (F)(u_1,\ldots ,u_r)=u_1\cdots u_r(u_1+\cdots +u_r) F(u_1,\ldots ,u_r) . \end{aligned}$$
  If $F=ma(f)$ as above, we have
  6.18 $$\begin{aligned} \Delta (F)=ma\bigl ([x,f(x,[x,y])]\bigr ) . \end{aligned}$$
  The inverse operator of $\Delta$ is given by
  6.19 $$\begin{aligned} \Delta ^{-1}(F)(u_1,\ldots ,u_r) = \frac{1}{u_1\cdots u_r(u_1+\cdots +u_r)} F(u_1,\ldots ,u_r) . \end{aligned}$$
  Of course, the operator $\Delta$ on power series given in (6.18) cannot always be inverted in the world of non-commutative power series.
- The *push-operator* acts on moulds *F* by the formula $push(F)(\emptyset )=F(\emptyset )$ and for $r\ge 1$,
  6.20 $$\begin{aligned} push(F)(u_1,\ldots ,u_r)=F(-u_1-\cdots -u_r,u_1,u_2,\ldots ,u_{r-1})\, . \end{aligned}$$
  The push-operator corresponds to an operation on power series (also called push) monomial by monomial defined as follows:
  6.21 $$\begin{aligned} push(x^{a_1}yx^{a_2}y\cdots yx^{a_r-1}yx^{a_r})=x^{a_r}yx^{a_1}y\cdots yx^{a_{r-2}}yx^{a_{r-1}} \end{aligned}$$
  in the sense that if $h\in {\mathbb {Q}}\langle \langle c_1,c_2,\ldots \rangle \rangle$ then
  6.22 $$\begin{aligned} ma\bigl (push(h)\bigr )=push\bigl (ma(h)\bigr ) , \end{aligned}$$
  where the left-hand push is as in (6.21) and the right-hand one is as in (6.20) (for this equivalence, see [90], section 3.3). In particular, *h* is push-invariant if and only if *ma*(*h*) is.
- The *swap* operator on moulds is defined by the formula $swap(F)(\emptyset )=F(\emptyset )$ and
  6.23 $$\begin{aligned} swap(F)(v_1,v_2,\ldots ,v_r)=F(v_r,v_{r-1}-v_r,\ldots ,v_1-v_2) . \end{aligned}$$
  We could write the mould *swap*(*F*) in the variables $u_i$ instead of $v_i$, of course, but to keep apart a mould and its swap it is convenient to consider the swapped mould parts $swap(F)_r$ as lying in ${\mathbb {Q}}(v_1,\ldots ,v_r)$.
- Finally, we need to define the *alternality* property on moulds. A mould $P\in ARI$ is said to be *alternal* if for all $r\ge 2$ we have 6.24 for all pairs of non-empty words $u=(u_1,\ldots ,u_i)$, $v=(u_{i+1},\ldots ,u_r)$. (There is no condition at $r=1$.) When $P=ma(p)$ for a power series $p\in {\mathbb {Q}}\langle \langle c_1,c_2,\ldots \rangle \rangle$ without constant term, then *P* is alternal if and only if *p* is a Lie element in the $c_i$, or equivalently, if and only if $p(x,y)\in \textrm{Lie}[x,y]$.

##### Example

Recall that the first non-trivial element of ${\mathfrak {mz}}^\vee$ is given by

6.25 $$\begin{aligned} g_3=[x,[x,y]]+[[x,y],y]=c_3+[c_2,c_1]=c_3+c_2c_1-c_1c_2 . \end{aligned}$$

By (6.2), the associated mould $G_3=ma(g_3)\in ARI$ is given by

6.26 $$\begin{aligned} 0&\mapsto 0=G_3(\emptyset )&\hbox {in depth 0} , \nonumber \\ c_3&\mapsto u_1^2=G_3(u_1)&\hbox {in depth 1} ,\nonumber \\ c_2c_1-c_1c_2&\mapsto -u_1+u_2=G_3(u_1,u_2)\  &\hbox {in depth 2} . \end{aligned}$$

The fact that $g_3$ is a Lie polynomial is reflected in the alternality condition satisfied by $G_3$:6.27

#### The fundamental operator $\textrm{Ad}_{ari}(pal)$ and Écalle’s theorem

Écalle defined a remarkable pair of inverse moulds in the group *GARI*, called *pal* and *invpal*, which have the following property: when acting on *ARI* via the adjoint action, *invpal* transforms the double shuffle property into a much simpler property known as *bialternality*, where a bialternal mould is an alternal mould with alternal swap, and *pal* does the opposite (this is a major result due to Écalle, see [28, 91] and an expository version in section 4.6 of [53]). The isomorphisms $\textrm{Ad}_{ari}(invpal)$ and $\textrm{Ad}_{ari}(pal)^{-1}$ are mutually inverse. The action of $\textrm{Ad}_{ari}(invpal)$ on a double shuffle Lie polynomial mould introduces certain denominators, but these are eliminated by the operator $\Delta$ in (6.17), yielding a polynomial mould once again (cf. [92]); in other words, restricted to $ma({\mathfrak {ds}})$, the composition $\Delta \circ \textrm{Ad}_{ari}(invpal)$ takes polynomial moulds to polynomial moulds. The key result for our purposes here is that when restricted to the subspace $ma({\mathfrak {mz}}^\vee )\subset ma({\mathfrak {ds}})$, the map $\Delta \circ \textrm{Ad}_{ari}(invpal)$ is directly related to the morphism

6.28 $$\begin{aligned} \gamma :{\mathfrak {mz}}^\vee \rightarrow \textrm{Der}^0\textrm{Lie}[a,b] \end{aligned}$$

of (5.26) by the following formula: if $h\in {\mathfrak {mz}}^\vee$, then

6.29 $$\begin{aligned} \Delta \circ \textrm{Ad}_{ari}(invpal)\bigl (ma(h)\bigr )=ma\bigl (\gamma (h)(a)\bigr ) , \end{aligned}$$

where

6.30 $$\begin{aligned} \gamma (h)\in \textrm{Der}^0\textrm{Lie}[a,b] \end{aligned}$$

and $\gamma (h)(a)$ denotes the Lie series obtained by applying that derivation to *a* (cf. [29], Thm. 1.3.1). The connection (6.29) enables us to apply the known properties of the operator $\textrm{Ad}_{ari}(invpal)$ to prove properties of the derivations $\tau _w$ and $\sigma _w$ in view of their relation to $\gamma$ in (5.28).

We now proceed to the definition of the moulds *pal* and *invpal*.

##### Definition 6.1.2

Let *dupal* be the mould defined explicitly by $dupal(\emptyset )=0$ and for $r>0$ by

6.31 $$\begin{aligned} dupal(u_1,\ldots ,u_r)=\frac{\textrm{B}_r}{r!} \frac{1}{u_1\cdots u_r} \Biggl (\sum _{j=0}^{r-1}(-1)^j \left( {\begin{array}{c}r-1\\ j\end{array}}\right) u_{j+1}\Biggr ) . \end{aligned}$$

##### Lemma 6.1.3

The mould *dupal* is related to $t_{01}$ in (5.30) by the equation

6.32 $$\begin{aligned} dupal(u_1,\ldots ,u_r)=\frac{1}{u_1\cdots u_r}ma(t_{01}^r) \end{aligned}$$

for all $r\ge 1$, where $t_{01}^r$ is the part of $t_{01}$ of *b*-degree *r*.

##### Proof

The map *ma* maps power series in *a*, *b* to moulds exactly like those in *x*, *y*, namely via (6.2) with $c_i=\textrm{ad}_a^{i-1}(b)$. To prove (6.32), notice that since we have

6.33 $$\begin{aligned} \textrm{ad}_b^{r-1}(a)=-\textrm{ad}_b^{r-2}([a,b])=-\textrm{ad}_{c_1}^{r-1}(c_2)=-\sum _{j=0}^{r-1}(-1)^j \left( {\begin{array}{c}r-1\\ j\end{array}}\right) c_1^jc_2c_1^{r-1-j} , \end{aligned}$$

the associated mould is

6.34 $$\begin{aligned} ma\bigl (\textrm{ad}_b^{r-1}(a)\bigr )=-\sum _{j=0}^{r-1}(-1)^j \left( {\begin{array}{c}r-1\\ j\end{array}}\right) u_{j+1} . \end{aligned}$$

Hence, since the part $t_{01}^r$ of *b*-degree *r* of $t_{01}$ is just given by $-\tfrac{\textrm{B}_r}{r!}\textrm{ad}_b^r(a)$, (6.32) follows from comparing (6.31) and (6.34).$\square$

##### Definition 6.1.4

Let *pal* be the mould defined recursively by $pal(\emptyset )=1$ and the formula

6.35 $$\begin{aligned} dur(pal)=mu(pal,dupal) \end{aligned}$$

with *dur* defined in (6.15) and *dupal* in (6.31).

This formula might look circular but in fact it defines each depth of *pal* successively thanks to the fact that $dupal(\emptyset )=0$. For example, in depth 1, we have

6.36 $$\begin{aligned} dur(pal)(u_1)&=u_1pal(u_1)\nonumber \\&=mu(pal,dupal)(u_1)\nonumber \\&=pal(\emptyset )dupal(u_1)+pal(u_1)dupal(\emptyset )\nonumber \\&=dupal(u_1)\nonumber \\&=-\frac{1}{2} , \end{aligned}$$

so

6.37 $$\begin{aligned} pal(u_1)=-\frac{1}{2u_1} . \end{aligned}$$

Then in depth 2, we have

6.38 $$\begin{aligned} dur(pal)(u_1,u_2)&=(u_1+u_2)pal(u_1,u_2)\nonumber \\&=mu(pal,dupal)(u_1,u_2)\nonumber \\&=pal(\emptyset )dupal(u_1,u_2)+pal(u_1)dupal(u_2)\nonumber \\&=\frac{u_1-u_2}{12u_1u_2} +\frac{1}{4u_1}\nonumber \\&= \frac{u_1+2u_2}{12u_1u_2} , \end{aligned}$$

so

6.39 $$\begin{aligned} pal(u_1,u_2)=\frac{u_1+2u_2}{12u_1u_2(u_1+u_2)} . \end{aligned}$$

##### Definition 6.1.5

Let $lopal=\log _{ari}(pal)$ using the ari-logarithm map defined in (6.13), and recall that *invpal* is the inverse of *pal* in the group $GARI=\exp _{ari}(ARI)$, equipped with the Baker–Campbell–Hausdorff multiplication law, so that we have

6.40 $$\begin{aligned} \log _{ari}(pal)=-\log _{ari}(invpal) . \end{aligned}$$

In lowest depths we have

6.41 $$\begin{aligned} lopal&=\bigg (0,\, -\frac{1}{2u_1}, \, \frac{u_1-u_2}{12u_1u_2(u_1+u_2)},\, \ldots \biggr ) ,\nonumber \\ invpal&=\biggl (1,\, \frac{1}{2u_1},\, \frac{-u_1+4u_2}{12u_1 u_2(u_1+u_2)},\, \ldots \biggr ) . \end{aligned}$$

Both of these moulds will be used below in our computations of $\sigma _w$.

The following theorem summarizes the key results from mould theory needed for the proof of Theorem 5.4.1 (i) to (iii).

##### Theorem 6.1.6

Let $h\in {\mathfrak {ds}}$ and let $H=ma(h)$ denote the associated mould. Let $\tau _h$ be the derivation of $\widehat{\textrm{Lie}}[a,b]$ constructed from *h* as in Sect. 5.3 and write $T_h=ma\bigl (\tau _h(a)\bigr )$. Then,

(i) The mould $\textrm{Ad}_{ari}(invpal)(H)$ is bialternal, i.e. it is alternal and its swap is alternal (cf. [28, 91] and [53], Thm. 4.6.1);
(ii) We have the following equality of moulds in *ARI* (cf. [29], Thm. 1.3.1):
  6.42 $$\begin{aligned} T_h=\Delta \circ \textrm{Ad}_{ari}(invpal)(H) ; \end{aligned}$$
(iii) All bialternal moulds are push-invariant (cf. [28, 53] Lemma 2.5.5); in particular $\textrm{Ad}_{ari}(invpal)(H)$ is push-invariant, and so is $T_h$ since $\Delta$ does not modify push-invariance;
(iv) A bialternal rational mould *A* satisfies
  6.43 $$\begin{aligned} A(-u_1,\ldots ,-u_r)=A(u_1,\ldots ,u_r) \end{aligned}$$
  for all $r\ge 1$. In particular if $A(u_1,\ldots ,u_r)$ is of odd total degree then it is equal to zero (cf. [53], Lemma 2.5.5).

Note that the push invariance of $T_h$ and therefore $\tau _h(a)$ established in part (iii) is crucial to obtain extensions of derivations of the Lie subalgebra $\textrm{Lie}[t_{12},t_{01}]\subset \widehat{\textrm{Lie}}[a,b]$ to all of $\textrm{Der}^0\widehat{\textrm{Lie}}[a,b]$, see the discussion around (5.37).

### Proof of Theorem 5.4.1 (i)–(iii)

For all $h\in {\mathfrak {mz}}^\vee$, let $\tau _h$ denote the associated derivation in $\textrm{Der}^0\textrm{Lie}[a,b]$ constructed in Sect. 5.3. Let $g_w$ for odd $w\ge 3$ be the canonical free generators of ${\mathfrak {mz}}^\vee$; recall that we write $\tau _w$ and $\sigma _w$ for the zeta generators in genus one rather than $\tau _{g_w}$ and $\sigma _{g_w}$. The results of Theorem 6.1.6 are valid for all elements $h\in {\mathfrak {ds}}$, in particular for elements of the subspace ${\mathfrak {mz}}^\vee \subset {\mathfrak {ds}}$, but in this section we will apply them specifically to the elements $g_w$.

#### Corollary 6.2.1

(Theorem 5.4.1 (i)). The derivations $\tau _w$ and $\sigma _w$ satisfy

6.44 $$\begin{aligned} \tau _w([a,b])= \sigma _w([a,b])=0 , \end{aligned}$$

i.e. $\tau _w$ and $\sigma _w$ lie in $\textrm{Der}^0 \textrm{Lie}[a,b]$.

#### Proof

The mould $T_w=ma\bigl (\tau _w(a)\bigr )$ is push-invariant by Theorem 6.1.6 (iii), and we saw in (6.22) that push-invariance for moulds is equivalent to push-invariance of power series. Thus $\tau _w(a)$ is push-invariant. It is shown in Lemma 2.1.1 of [29] that for any derivation $\delta$ of $\textrm{Lie}[a,b]$ such that $\delta (b)$ is the partner of $\delta (a)$ as defined in (5.31), then $\delta ([a,b])=0$ if and only if $\delta (a)$ is push-invariant. Since $\tau _w(b)$ is the partner of $\tau _w(a)$ by construction (i.e. (5.31) with $g=\tau _w(a)$ and $g' = \tau _w(b)$) and $\tau _w(a)$ is push-invariant, we thus have $\tau _w([a,b])=0$ as desired. Then

6.45 $$\begin{aligned} \sigma _w([a,b])=\theta \circ \tau _w\circ \theta ([a,b])=\theta \circ \tau _w([b,a])=0 \end{aligned}$$

as well.$$\square $$

#### Proposition 6.2.2

The mould $T_w$ is zero in all even depths, and in odd depths $r\ge 1$, $T_w(u_1,\ldots ,u_r)$ is a polynomial of homogeneous degree $w+1$ in the variables $u_i$. In particular

6.46 $$\begin{aligned} T_w(u_1)=u_1^{w+1} . \end{aligned}$$

#### Proof

We first show that the mould $T_w$ is of constant degree $w+1$ in $u_1,\ldots ,u_r$ in every depth. For this, we begin by noting that the Lie series

6.47 $$\begin{aligned} \tau _w(t_{01})=[t_{01},g_w(t_{12},-t_{01})] \end{aligned}$$

has constant *a*-degree equal to $w+1$ since $g_w$ is a polynomial of homogeneous degree *w* and both $t_{01}$ and $t_{12}$ have *a*-degree 1. Then, using the degree-by-degree computation of $\tau _w(a)$ given in (5.34) to (5.37) (with $h=g_w$), we see that $\tau _w(a)_n$ is a Lie polynomial of constant *a*-degree $w+1$ in every degree *n* since the *a*-degree of the partner $\tau _w(b)$ is one less than that of $\tau _w(a)$ at every degree. By the defining property $g_w(x,y)|_{x^{w-1}y}=1$ of the canonical polynomials in genus zero and their symmetry property $g_w(x,y) = g_w(y,x)$,^12^ the monomial $y^{w-1}x$ also appears in $g_w(x,y)$ with coefficient 1. Since $g_w(x,y)$ for odd *w* is a Lie polynomial this implies that the Lie word $\textrm{ad}_y^{w-1}(x)$ appears in $g_w$ with coefficient 1. Thus the minimal *x*-degree in $g_w$ is 1 and by (5.36) we have

6.48 $$\begin{aligned} \tau _w(a)_{w+2}=[a,\textrm{ad}_a^{w-1}([a,b])]=\textrm{ad}_a^{w+1}(b) , \end{aligned}$$

where the sign in $t_{01} = -a + \ldots$ disappears since *w* is odd.

Under the map *ma* from power series to commutative variables $u_1,\ldots ,u_r$ defined in (6.2) (with $c_i=\textrm{ad}_a^{i-1}b$ for $i\ge 1$), we see that the *a*-degree corresponds to the degree in $u_1,\ldots ,u_r$ while the *b*-degree corresponds to the mould depth *r*; thus for all $r\ge 1$, the depth *r* part of the mould $T_w=ma\bigl (\tau _w(a)\bigr )$ is a polynomial in $u_1,\ldots ,u_r$ of degree $w+1$. Furthermore, the lowest depth part of $T_w$ appears in depth 1 and is given by

6.49 $$\begin{aligned} T_w(u_1)=ma\bigl (\textrm{ad}_a^{w+1}(b)\bigr )=u_1^{w+1} . \end{aligned}$$

It remains only to prove that $T_w(u_1,\ldots ,u_r)=0$ for all even *r*. For this, we apply Theorem 6.1.6 to the case $h=g_w$ and $H=G_w=ma(g_w)$. By (ii) of that theorem, we have

6.50 $$\begin{aligned} T_w=\Delta \circ \textrm{Ad}_{ari}(invpal)(G_w) . \end{aligned}$$

Therefore for each $r\ge 1$ we have

6.51 $$\begin{aligned} \Delta ^{-1}(T_w)(u_1,\ldots ,u_r)=\frac{T_w(u_1,\ldots ,u_r)}{u_1\cdots u_r(u_1+\cdots +u_r)}=\textrm{Ad}_{ari}(invpal)(G_w)(u_1,\ldots ,u_r) . \end{aligned}$$

By (i) of Theorem 6.1.6, the mould $\textrm{Ad}_{ari}(invpal)(G_w)$ is bialternal, so the rational mould in the middle term is bialternal. The total degree of this rational function is $w-r$, which is odd whenever *r* is even. Thus, by Theorem 6.1.6 (iv), the mould $T_w$ is zero in all even depths *r*. This concludes the proof of the Proposition. $\square$

#### Corollary 6.2.3

(Theorem 5.4.1 (ii)).

(i) The minimal degree part of the Lie series $\tau _w(a)$ is equal to $\textrm{ad}_a^{w+1}(b)$, so the minimal degree part of $\tau _w$ is $\epsilon _{w+1}$. The minimal degree part of $\sigma _w$ is given by $-\tfrac{1}{(w-1)!}\epsilon _{w+1}^{(w-1)}$.
(ii) There are no terms of degree $<w+2$ and no terms of even degree in the Lie series $\tau _w(a)$, $\sigma _w(a)$ and their partners. For all odd $n\ge w+2$, the degree-*n* terms of $\tau _w(a)$ (resp. $\sigma _w(b)$) all have *b*-degree (resp. *a*-degree) equal to $n-w-1$ and constant *a*-degree (resp. constant *b*-degree) equal to $w+1$.

#### Proof

(i) We saw in (6.48) that the lowest degree of $\tau _w(a)$ is $w+2$ and $(\tau _w(a))_{w+2}=\textrm{ad}_a^{w+1}(b)$, which is also equal to $\epsilon _w(a)$ by (5.2). The switch formula is given in (5.13).

(ii) The statement is a direct translation of the corresponding statement of the previous proposition into terms of the non-commutative variables *a*, *b*. The minimal degree of $\tau _w$ and $\sigma _w$ as a derivations is $w+1$ by part (i), so the minimal degree of the Lie series $\tau _w(a)$ and $\sigma _w(a)$ is $w+2$. For the other terms, the map *ma* sends a polynomial $h\in {\mathbb {Q}}\langle c_1,c_2,\ldots \rangle$ (with $c_i=\textrm{ad}_a^{i-1}(b)$) of homogeneous degree *n* in *a*, *b* and homogeneous depth *r* to a mould *ma*(*h*) concentrated in depth *r* of homogeneous degree $n-r$ in the variables $u_1,\ldots ,u_r$. Since the degree of $T_w(u_1,\ldots ,u_r)$ is always $w+1$ by the previous Proposition, the *a*-degree of every term of $\tau _w(a)$ is $w+1$. The depth *r* part of the mould $T_w$ corresponds to the *b*-degree *r* part of the power series $\tau _w(a)$. We first observe that if *r* is even then $T_w(u_1,\ldots ,u_r)=0$ by the previous proposition, so all terms of $\tau _w(a)$ of even *b*-degree *r* are zero, but these are precisely all the terms of total degree $w+1+r$, which are all of the even-degree terms. If we have a term $\tau _w(a)$ of odd total degree *n*, then since it has *a*-degree $w+1$ its *b*-degree is equal to $n-w-1$. This concludes the proof for $\tau _w(a)$ and the switch gives the analogous result for $\sigma _w(b)$ with *b*-degree $w+1$ and *a*-degree $n-w-1$. $\square$

#### Proposition 6.2.4

For each odd $w\ge 3$, the mould $T_w=ma\bigl (\tau _w(a)\bigr )$ is entirely determined by its parts of depth $r\le w-1$.

#### Proof

By Theorem 6.1.6 (ii), the mould $\Delta ^{-1}T_w$ is equal to $\textrm{Ad}_{ari}(invpal)(G_w)$ where $G_w=ma(g_w)$ and $g_w$ is the canonical polynomial in genus zero. For any moulds $P\in GARI$ and $A\in ARI$, set $L=\log _{ari}(P)$ and recall the adjoint operator formula (6.14). Since *L* has no constant term, taking the *ari*-bracket with *L* increases the depth, so the adjoint operator formula shows that for any given depth *r*, only the terms of *A* of depth $\le r$ contribute to the depth *r* part of $\textrm{Ad}_{ari}(P)(A)$. Now let $A=\textrm{Ad}_{ari}(invpal)(G_w)$ and $P=pal$, so that

6.52 $$\begin{aligned} \textrm{Ad}_{ari}(P)(A)=\textrm{Ad}_{ari}(pal)\bigl (\textrm{Ad}_{ari}(invpal)(G_w)\bigr )=G_w\, .\end{aligned}$$

Since $g_w$ is a Lie polynomial of degree *w* it has no terms of depth $\ge w$, so the same is true for the associated mould $G_w=ma(g_w)$. Thus, $G_w$ is determined entirely by its parts of depth $\le w-1$, which in turn by the adjoint action formula are determined entirely by the parts of $A=\textrm{Ad}_{ari}(invpal)(G_w)$ in depths $\le w-1$. The parts of $T_w$ of depth $\le w-1$ determine those of $A=\textrm{Ad}_{ari}(invpal)(G_w)$ by applying $\Delta ^{-1}$, and the parts of *A* of depths $\le w-1$ then determine $G_w$ up to depth $w-1$ by the adjoint action formula (6.52) – but this is all of $G_w$, which then in turn determines all of $T_w$ by the formula

6.53 $$\begin{aligned} T_w=\Delta \circ \textrm{Ad}_{ari}(invpal)(G_w) , \end{aligned}$$

concluding the proof of the proposition.$\square$

#### Corollary 6.2.5

(Theorem 5.4.1 (iii)). Both of the derivations $\tau _w$ and $\sigma _w$ are entirely determined by their parts of degree $\le 2w-1$ (as derivations).

#### Proof

By the above Proposition, $T_w$ is entirely determined by its parts of depth $\le w-1$, so the same holds for the Lie series $\tau _w(a)$. But we saw above that for all $r\ge 1$ the *b*-degree *r* part of the Lie series $\tau _w(a)$ is of polynomial degree $w+r+1$ in *a* and *b*, so in particular the *b*-degree $w-1$ part of $\tau _w(a)$ is of degree 2*w*. Saying that $\tau _w(a)$ is determined by its parts of *b*-degree $\le w-1$ is equivalent to saying that it is determined by its parts of total degree $\le 2w$. Since $\tau _w([a,b])=0$ by Corollary 6.2.1, knowing $\tau _w(a)$ determines $\tau _w$ completely. The part of $\tau _w(a)$ of given polynomial degree *n* corresponds to the part of $\tau _w$ of degree $n-1$ as a derivation; thus the derivation $\tau _w$ is entirely determined by its parts of degree $\le 2w-1$, and the same holds for $\sigma _w$.$\square$

### Proof of Theorem 5.4.1 (iv)–(vi)

In this section, we use properties of the $\mathfrak {sl}_2$ algebra in Definition 5.1.3 with generators $\epsilon _0,\epsilon _0^\vee ,\textrm{h}$ to prove parts (iv)-(vi) of Theorem 5.4.1.

Since the element $\textrm{h}=[\epsilon _0,\epsilon _0^\vee ]\in \mathfrak {sl}_2\subset \textrm{Der}^0\textrm{Lie}[a,b]$ acts by $\textrm{h}(a)=-a$ and $\textrm{h}(b)=b$, any derivation $\delta$ of $\widehat{\textrm{Lie}}[a,b]$ of homogeneous *a*-degree $\alpha$ and *b*-degree $\beta$ is an eigenvector for $\textrm{h}$, with eigenvalue given by

6.54 $$\begin{aligned} {[}\textrm{h},\delta ]=(\beta -\alpha )\delta . \end{aligned}$$

In particular, for the action of $\textrm{h}$ on $\mathfrak {u}$, we have $[ \textrm{h}, \epsilon _k^{(j)}] = (2j{+}2{-}k) \epsilon _k^{(j)}$ from (5.11), so $\textrm{h}$ has eigenvalues covering the spectrum of values ${-}k{+}2,\, {-}k{+}4,\ldots ,-2,0,2,\ldots ,k{-}4,\, k{-}2$ within the $(k-1)$-dimensional irreducible representations $\{ \epsilon _k^{(j)}, \ j=0,1,\ldots ,k-2\}$ of $\mathfrak {sl}_2$ at fixed *k*. Similarly, $(r-1)$-dimensional irreducible subrepresentations in $\mathfrak {u}$ built from brackets of $\epsilon _{k_1}^{(j_1)} \epsilon _{k_2}^{(j_2)}\ldots \epsilon _{k_m}^{(j_m)}$ will have the spectrum of $\textrm{h}$-eigenvalues ${-}r{+}2,\, {-}r{+}4,\ldots ,-2,0,2,$ $\ldots ,r{-}4,\, r{-}2,$ always including the eigenvalue zero since *r* is even as will become clear from the discussion around (7.3).

By $[\textrm{h},\epsilon _0]= 2 \epsilon _0$ and $[\textrm{h},\epsilon _0^\vee ]= - 2 \epsilon _0^\vee$, adjoint action of $\epsilon _0$ and $\epsilon _0^\vee$ shifts the $\textrm{h}$ eigenvalue of any derivation $\delta \in \textrm{Der}^0\widehat{\textrm{Lie}}[a,b]$ (not necessarily $\delta \in \mathfrak {u}$) by 2 and $-2$, respectively (except for highest- and lowest-weight vectors annihilated by $\textrm{ad}_{\epsilon _0}$ and $\textrm{ad}_{\epsilon ^\vee _0}$, respectively).

#### Lemma 6.3.1

By the above spectra of $\textrm{h}$ eigenvalues in irreducible representations of $\mathfrak {sl}_2$ and the action (5.12) as well as the fact that $\textrm{ad}_{\epsilon _0}\epsilon _k^{(j)}=\epsilon _k^{(j+1)}$ and $\epsilon _k^{(k-1)}=0$, we have:

(i) for any $Y \in \textrm{ad}_{\epsilon _0}\mathfrak {u}$, the equation $\textrm{ad}_{\epsilon _0}X=Y$ has a unique solution $X\in \textrm{ad}_{\epsilon _0^\vee }\mathfrak {u}$. In particular, $\textrm{ad}_{\epsilon _0}$ has no kernel within eigenspaces at negative eigenvalues of $\textrm{h}$.
(ii) for any $Y \in \textrm{ad}_{\epsilon _0^\vee }\mathfrak {u}$, the equation $\textrm{ad}_{\epsilon _0^\vee }X=Y$ has a unique solution $X\in \textrm{ad}_{\epsilon _0}\mathfrak {u}$. In particular, $\textrm{ad}_{\epsilon _0^\vee }$ has no kernel at positive eigenvalues of $\textrm{h}$.

#### Proof of Theorem 5.4.1 (iv)

For any term of $\sigma _w$ of total degree *n*, since by Theorem 5.4.1 (ii) the *b*-degree is *w*, the *a*-degree must be $n-w$, and thus by (6.54) this term is an $\textrm{h}$-eigenvector with $\textrm{h}$-eigenvalue equal to $2w - n$. Thus any term of $\sigma _w$ of bihomogeneous degree in *a* and *b* and total degree *n* is an eigenvector for $\textrm{h}$, and we have:

6.55 $$\begin{aligned}&\hbox {if }n<2w, \hbox {the eigenvalue of }\textrm{h}\hbox { is strictly positive,} \nonumber \\&\hbox {if }n=2w,\hbox { the eigenvalue of }\textrm{h}\hbox { is zero,}\nonumber \\&\hbox {if }n>2w,\hbox { the eigenvalue of }\textrm{h}\hbox { is negative}. \end{aligned}$$

##### Lemma 6.3.2

(Theorem 5.4.1 (iv)). The derivation $\sigma _w$ has no highest-weight vectors in degrees $n>2w$.

##### Proof

Since $\textrm{ad}_{\epsilon _0}$ has no kernel at negative $\textrm{h}$-eigenvalues by Lemma 6.3.1 (i), the infinite Lie series of geometric contributions to $\sigma _w$ above key degree 2*w* does not involve any highest-weight vectors. $\square$

#### Proof of Theorem 5.4.1 (v) and (vi)

We shall next prove parts (v) and (vi) of Theorem 5.4.1 based on Theorem 5.2.1. In a notation where

6.56 $$\begin{aligned} \mathfrak {g}&:=\mathfrak {u} \rtimes \mathfrak {sl}_2 , \end{aligned}$$

and ${{\mathcal {S}}}$ denotes the free Lie algebra of zeta generators $\sigma _w$, Theorem 5.2.1 implies that

6.57 $$\begin{aligned} {[}\mathfrak {g},{{\mathcal {S}}}]\subset \mathfrak {g} . \end{aligned}$$

Following the notation $p_d$ for degree-*d* parts of polynomials *p* in *a*, *b*, we shall write $(\sigma _w)_d$ for the degree-*d* part of genus one zeta generators, so that in particular $\sigma _w^{\textrm{key}} = (\sigma _w)_{2w}$.

##### Proposition 6.3.3

(Theorem 5.4.1 (v) and (vi)).

(i) All terms of $\sigma _w$ in degrees $\ne 2w$ lie in $\mathfrak {u}$, but $\sigma _w^{\textrm{key}}\notin \mathfrak {u}$.
(ii) The terms of $\sigma _w$ in key degree 2*w* that lie in irreducible $\mathfrak {sl}_2$ representations of dimension $\ge 3$ lie in $\mathfrak {u}$.
(iii) The brackets $[z_w,\epsilon _k]$ of the $\mathfrak {sl}_2$-invariant part $z_w$ of $\sigma _w$ lie in $\mathfrak {u}$.

##### Proof

(i) Recall from Theorem 5.4.1 (ii) that every term of $\sigma _w$ is of *b*-degree *w* and that the minimum total degree of any term is given by $n=w+1$. Let

6.58 $$\begin{aligned} \sigma _w = \sum _{n=w+1}^\infty (\sigma _w)_n \end{aligned}$$

denote the expansion of $\sigma _w$ according to total degree. Then by (6.54), for each $n\ge w+1$, we have

6.59 $$\begin{aligned} {[}\textrm{h}, (\sigma _w)_n] = (2w-n) (\sigma _w)_n . \end{aligned}$$

Note that, instead of (6.57), we actually have the stronger statement

6.60 $$\begin{aligned} {[}\mathfrak {g},{{\mathcal {S}}}]\subset \mathfrak {u} \end{aligned}$$

since the brackets on the left-hand cannot have any terms of degree zero and $\mathfrak {u}$ is the part of $\mathfrak {g}$ of degree $>0$. Thus, the bracket $[\textrm{h},\sigma _w]$ must lie in $\mathfrak {u}$ and indeed each separate term $[\textrm{h},(\sigma _w)_n ]$ must already lie in $\mathfrak {u}$ since there are no linear relations between terms of different degree. Hence, by (6.59), we must have

6.61 $$\begin{aligned} (2w-n) (\sigma _w)_n \in \mathfrak {u} \end{aligned}$$

for all $n\ge w+1$, i.e. for all terms of $\sigma _w$. In particular, whenever $2w-n\ne 0$, (6.61) implies that $(\sigma _w)_n \in \mathfrak {u}$. Terms of $\sigma _w$ not in $\mathfrak {u}$ can thus only occur when $n=2w$, i.e. in key degree. The fact that $\sigma _w^{\textrm{key}}\notin \mathfrak {u}$ follows directly from Theorem 5.2.1, since if $\sigma _w^{\textrm{key}}$ lied in $\mathfrak {u}$ then we would have $\sigma _w \in \mathfrak {u}$, so $\mathfrak {u}$ together with the $\sigma _w$ could not generate a semi-direct product as in Theorem 5.2.1 (ii).

(ii) Once again, by (6.60), any bracket of $\mathfrak {sl}_2$ elements and $\sigma _w$, and therefore in particular $[\epsilon _0,(\sigma _w)_{2w}]$ must lie in $\mathfrak {u}$. If we decompose

6.62 $$\begin{aligned} (\sigma _w)_{2w}= \sum _{\textrm{odd}\ d\ge 1} (\sigma _w)_{2w}^{(d)} , \end{aligned}$$

where $(\sigma _w)_{2w}^{(d)}$ collects the key-degree terms in $\sigma _w$ that lie in *d*-dimensional irreducible representations of $\mathfrak {sl}_2$, we must then have

6.63 $$\begin{aligned} {[}\epsilon _0, (\sigma _w)_{2w}^{(d)}] \in \mathfrak {u} \end{aligned}$$

separately for each (odd) $d\ge 1$. When $d\ge 3$, the terms $[\epsilon _0, (\sigma _w)_{2w}^{(d)} ]\in \mathfrak {u}$ are non-zero since highest-weight vectors of $(d\ge 3)$-dimensional $\mathfrak {sl}_2$ representations have $\textrm{h}$-eigenvalue $\ge 2$. Then, thanks to the equality^13^

6.64 $$\begin{aligned} (\sigma _w)_{2w}^{(d)} = \frac{4}{(d-1)(d+1)} [\epsilon _0^\vee ,[\epsilon _0, (\sigma _w)_{2w}^{(d)}]] , \end{aligned}$$

we see that for $d\ge 3$, the term $(\sigma _w)_{2w}^{(d)}$ itself lies in $\mathfrak {u}$ since $\mathfrak {u}$ is an $\mathfrak {sl}_2$-module by Theorem 5.2.1.

When $d=1$, the term $[\epsilon _0,(\sigma _w)_{2w}^{(1)}]=0$ and therefore we cannot use (6.63) to conclude that $(\sigma _w)_{2w}^{(1)}$ lies in $\mathfrak {u}$; indeed we know that it cannot lie in $\mathfrak {u}$ since otherwise all of $\sigma _w$ would, contradicting (i). This proves that the arithmetic terms $z_w$ of $\sigma _w$ form a one-dimensional $\mathfrak {sl}_2$ representation in key degree.

Finally, (iii) follows directly from (6.60), since this shows that $[\epsilon _k,\sigma _w]\in \mathfrak {u}$ and $z_w$ is the only term of $\sigma _w$ not already in $\mathfrak {u}$. $\square$

## Recursive High-Order Computations of $\sigma _w$ and [$z_w,\epsilon _k$]

In this section, we combine representation theory of $\mathfrak {sl}_2$ with Theorem 5.4.1, particularly part (vii) recalled below, to perform explicit high-order computations of $\sigma _w$ and $[z_w,\epsilon _k]$ in terms of nested brackets of $\epsilon _k^{(j)}$.

### Proof and first consequences of Theorem 5.4.1 (vii)

#### Proposition 7.1.1

(Theorem 5.4.1 (vii)). Let $\textrm{BF}_k :=\frac{ \textrm{B}_k}{k!}$ for $k\ge 2$, and set

7.1 $$\begin{aligned} N :=-\epsilon _0 + \sum _{k=4}^{\infty } (k-1)\textrm{BF}_k \epsilon _k . \end{aligned}$$

Then for all odd $w\ge 3$ we have

7.2 $$\begin{aligned} {[}N,\sigma _w]=0\in \textrm{Der}^0\widehat{\textrm{Lie}}[a,b] . \end{aligned}$$

#### Proof

The proof of this result is given in section 27 of [27] based on sections 12 and 13 of [89], so we simply indicate the essential argument here. In the framework set forth in Remark 5.2.2, we noted that the two profinite groups $\widehat{\textrm{SL}}_2(\mathbb {Z})$ and the absolute Galois group $\textrm{Gal}({\overline{{\mathbb {Q}}}}/{\mathbb {Q}})$ both act naturally as automorphisms on the profinite fundamental group ${\hat{\pi }}_1(E_\infty )$ of the nodal elliptic curve, where $\textrm{SL}_2(\mathbb {Z})$ is identified with the fundamental group of the moduli space ${{\mathcal {M}}}_{1,1}$. There is a distinguished element in $\widehat{\textrm{SL}}_2(\mathbb {Z})$ on which $\textrm{Gal}({\overline{{\mathbb {Q}}}}/{\mathbb {Q}})$ acts via its abelian quotient $\textrm{Gal}({\overline{{\mathbb {Q}}}}^{\textrm{ab}}/{\mathbb {Q}})$: this is the element corresponding to a small loop around the degenerate point $\tau =i\infty$ in the moduli space (or as Hain–Matsumoto describe it, a small loop around $q=0$ in the *q*-disk where $q=e^{2\pi i\tau }$). Thus in the pro-unipotent version, or rather the associated Lie algebra version, the arithmetic part ${{\mathcal {S}}}$ corresponding to the Galois action commutes with the image of this element in the Lie algebra $\mathfrak {u}\rtimes \mathfrak {sl}_2$. There are various ways of showing that this image is equal to the element *N* defined in (7.1); the method used in section 12 of [89] is to identify it as the residue at $q=0$ of the restriction of the KZB connection (see “7.4.4”) to a first order neighborhood of the degenerate nodal curve. $\square$

In the remainder of this section, the commutation relation (7.2) will be applied to recursively determine the *infinite* series expansions of $\sigma _w$ as in (5.46) to (5.50) from the *finitely* many terms in degree $\le 2w$. The finitely many contributions to $\sigma _w$ not yet determined by (7.2) are precisely the highest-weight vectors of $\mathfrak {sl}_2$, i.e. the elements in the kernel of $\textrm{ad}_{\epsilon _0}$. By Theorem 5.4.1 (iv), these highest-weight-vector contributions to $\sigma _w$ occur only up to and including key degree 2*w* which explains the finite number of them for each *w*.

For example, when $w=3$, the key degree is 6 and feeding the highest-weight vector contributions $-\frac{1}{2} \epsilon _4^{(2)}$ and $z_3$ into (7.2) determines all of $\sigma _3$, see (7.22) below for the exact result. When $w=5$, the highest-weight vectors $-\frac{1}{24} \epsilon _6^{(4)}, \, -\frac{5 }{48} [\epsilon _4^{(1)},\epsilon _4^{(2)}]$ and $z_5$ occurring in the low-degree part of $\sigma _5$ feed into (7.2) and determine all of $\sigma _5$.^14^

Our construction of $\sigma _w$ from finitely many highest-weight vectors will be recursive in the *modular depth* of its geometric contributions which we define as follows:

#### Definition 7.1.2

Nested brackets $[[\ldots [[\epsilon _{k_1}^{(j_1)} ,\epsilon _{k_2}^{(j_2)} ], \epsilon _{k_3}^{(j_3)} ] ,\ldots ], \epsilon _{k_r}^{(j_r)}]$ of *r* derivations $\epsilon _k^{(j)}$ in $\mathfrak {u}$ are said to have *modular depth* *r*. The modular depth forms a natural increasing filtration on $\mathfrak {u}$, but not a grading, as shown for example by the Pollack relation (5.19) which can be viewed as an equality between linear combinations of terms of modular depth 2 with two terms of modular depth 3.

In addition to the infinitely many terms in the series expansion of $\sigma _w$ above key degree, the recursive method of Sect. 7.3 will completely determine the explicit form of the brackets $[z_w,\epsilon _k]$ of the arithmetic contributions $z_w$ to the zeta generators. We reiterate that, by Theorem 5.4.1 (v) and (vi), the non-geometric part $z_w$ of $\sigma _w$ is concentrated in a one-dimensional $\mathfrak {sl}_2$ representation at key degree 2*w* and gives rise to brackets $[z_w,\epsilon _k] \in \mathfrak {u}$.

### $\mathfrak {sl}_2$ prerequisites

We start by organizing $\mathfrak {u}$ into representations of the subalgebra $\mathfrak {sl}_2$ of $\textrm{Der}^0\textrm{Lie}[a,b]$, and describing its irreducible pieces; in particular we determine the highest- and lowest-weight vectors of each one.

In view of the nilpotency $\textrm{ad}_{\epsilon _0}^{k-1} \epsilon _k =0$ (see (5.8)), the non-zero $\epsilon _k^{(j)}=\textrm{ad}_{\epsilon _0}^{j} \epsilon _k$ for fixed even *k* and $j=0,1,\ldots ,k-2$ form a $(k-1)$-dimensional irreducible representation of $\mathfrak {sl}_2$, which we denote by $V(\epsilon _k)$. The generators $\epsilon _0,\epsilon _0^\vee ,\textrm{h}$ of $\mathfrak {sl}_2$ permute the elements of $V(\epsilon _k)$ simply by $\textrm{ad}_{\epsilon _0}\epsilon _k^{(j)}=\epsilon _k^{(j+1)}$, (5.11) and (5.12), identifying $\textrm{ad}_{\epsilon _0}$ and $\textrm{ad}_{\epsilon _0^\vee }$ as the raising and lowering operators for the eigenvalues of $\textrm{h}$, respectively. All irreducible representations of $\mathfrak {sl}_2$ inside $\mathfrak {u}$ are formed from nested commutators of the $\epsilon _k^{(j)}$, and they are all isomorphic (as $\mathfrak {sl}_2$-representations) to some $V(\epsilon _k)$ for even $k\ge 2$. Note that each odd-dimensional $\mathfrak {sl}_2$-representation occurs infinitely many times in $\mathfrak {u}$, and they can be arranged by modular depth.

The collections of commutators $[\epsilon _{k_1}^{(j_1)},\epsilon _{k_2}^{(j_2)}]$ for fixed $k_1,k_2$ and $j_i=0,1,\ldots ,k_i-2$ sit inside the reducible tensor-product representations $V(\epsilon _{k_1})\otimes V(\epsilon _{k_2})$ of $\mathfrak {sl}_2$ which can be decomposed into the following $(r-1)$-dimensional irreducible representations $V(\epsilon _r)$ of $\mathfrak {sl}_2$:

7.3 $$\begin{aligned} V(\epsilon _{k_1})\otimes V(\epsilon _{k_2}) = \bigoplus _{\begin{array}{c} r= |k_1-k_2|+2\\ r \in 2{\mathbb {Z}} \end{array}}^{k_1+k_2-2} V(\epsilon _r) . \end{aligned}$$

Since *r* is restricted to even values, the dimensions of the irreducible representations of $\mathfrak {sl}_2$ in iterated tensor products of $V(\epsilon _{k_i})$ are always odd.

#### Projectors to lowest-weight vectors

The projection of the commutators $[\epsilon _{k_1}^{(j_1)},\epsilon _{k_2}^{(j_2)}]$ at modular depth two into the irreducible representations $V(\epsilon _r)$ on the right-hand side of (7.3) is implemented by

7.4 $$\begin{aligned} t^d(\epsilon _{k_1},\epsilon _{k_2}) :=\frac{(d{-}2)!}{(k_1{-}2)!(k_2{-}2)!}\sum _{i=0}^{d-2} (-1)^i \frac{(k_1{-}2{-}i)! (k_2{-}d{+}i)!}{i! (d{-}2{-}i)!} [ \epsilon _{k_1}^{(i)} , \epsilon _{k_2}^{(d-2-i)} ] \end{aligned}$$

with $d = \frac{1}{2}(k_1+k_2-r+2)$ and therefore $2\le d \le \textrm{min}(k_1,k_2)$. In case of $k_1=k_2$, the $t^d(\epsilon _{k},\epsilon _{k})$ at even values of *d* vanish.

The outcomes $t^d(\epsilon _{k_1},\epsilon _{k_2})$ of the projectors in (7.4) are lowest-weight vectors (see Definition 5.1.3) of the $V(\epsilon _r)$ in the tensor product (7.3). The rest of the $(r-1)$-dimensional irreducible representations in $\mathfrak {u}$ at modular depth two is obtained from $\textrm{ad}_{\epsilon _0}^j t^d(\epsilon _{k_1},\epsilon _{k_2})$ with $j=0,1,\ldots ,r-2$ and terminates due to $\textrm{ad}_{\epsilon _0}^{r-1} t^d(\epsilon _{k_1},\epsilon _{k_2})=0$.

Since $t^{d_1}(\epsilon _{k_1},\epsilon _{k_2})$ is a lowest-weight vector it can be inserted on the same footing as $\epsilon _r$ with $r=k_1+k_2-2d_1+2$ into another operation (7.4). For instance,

7.5 $$\begin{aligned} t^{d_2}(\epsilon _{k_3},t^{d_1}(\epsilon _{k_1},\epsilon _{k_2}))&= \frac{(d_2{-}2)!}{(k_3{-}2)!(r{-}2)!}\sum _{i=0}^{d_2-2} (-1)^i \frac{(k_3{-}2{-}i)! (r{-}d_2{+}i)!}{i! (d_2{-}2{-}i)!}\nonumber \\&\quad \times [ \epsilon _{k_3}^{(i)} , \textrm{ad}_{\epsilon _0}^{d_2-2-i} t^{d_1}(\epsilon _{k_1},\epsilon _{k_2})] \end{aligned}$$

is the lowest-weight vector of a $(k_1+k_2+k_3-2d_1-2d_2+3)$-dimensional irreducible $\mathfrak {sl}_2$ representation in the triple tensor product $V(\epsilon _{k_1})\otimes V(\epsilon _{k_2})\otimes V(\epsilon _{k_3})$ which may be decomposed into irreducibles by iterating (7.3). Iterations of the $t^d$ projectors (7.4) as exemplified in (7.5) are instrumental for compactly representing the contributions to $[z_w,\epsilon _k]$ at modular depth three in Sect. 7.4.2 below.

#### Projectors to highest-weight vectors

One can similarly generate highest-weight vectors of the the irreducible representations $V(\epsilon _r)$ in $V(\epsilon _{k_1})\otimes V(\epsilon _{k_2})$ and tensor products at higher modular depth via

7.6 $$\begin{aligned} s^d(\epsilon _{k_1},\epsilon _{k_2}) :=\frac{(d{-}2)! }{(k_1{-}2)! (k_2{-}2)!} \sum _{i=0}^{d-2} (-1)^i [ \epsilon _{k_1}^{(k_1-2-i)}, \epsilon _{k_2}^{(k_2-d+i)}] \end{aligned}$$

where again $d = \frac{1}{2}(k_1+k_2-r+2)$, as long as $2\le d \le \textrm{min}(k_1,k_2)$. Nevertheless, we will see that an extension of (7.6) to $d>\textrm{min}(k_1,k_2)$ will be useful to bring certain contributions to $\sigma _w$ into a convenient form, though the highest-weight vector property $[\epsilon _0, s^d(\epsilon _{k_1},\epsilon _{k_2})]=0$ only holds for $d \le \textrm{min}(k_1,k_2)$. Since the entries $\epsilon _{k_1},\epsilon _{k_2}$ of the $s^d$-operation in (7.6) are lowest-weight vectors, the nested brackets relevant to modular depth $m\ge 3$ are generated by *m* iterations of $t^{d_i}$ and a single $s^d$ operation for the outermost bracket. For instance,

7.7 $$\begin{aligned} s^{d_2}(\epsilon _{k_3},t^{d_1}(\epsilon _{k_1},\epsilon _{k_2}))&= \frac{(d_2{-}2)! }{(k_3{-}2)! (r{-}2)!} \sum _{i=0}^{d_2-2} (-1)^i [ \epsilon _{k_3}^{(k_3-2-i)},\textrm{ad}_{\epsilon _0}^{k_2-d_2+i} t^{d_1}(\epsilon _{k_1},\epsilon _{k_2}) ] \end{aligned}$$

at suitable values for $d_1,d_2$ (with $r=k_1+k_2-2d_1+2$) generate all highest-weight vectors of the irreducible $\mathfrak {sl}_2$ representations in $V(\epsilon _{k_1})\otimes V(\epsilon _{k_2}) \otimes V(\epsilon _{k_3})$. In general, iterations of $s^{d_{m-1}} t^{d_{m-2}}\ldots t^{d_1}$ conveniently capture the highest-weight-vector contributions to $\sigma _w$ at each modular depth that are not yet determined by the recursion below based on $[N,\sigma _w]=0$ (see Theorem 5.4.1 (vii)).

#### $\mathfrak {sl}_2$ representations of Pollack relations

The Pollack relations among $\epsilon _k^{(j)}$ with $k\ge 4$ and $0\le j \le k-2$ in Remark 5.1.6 fall into irreducible $\mathfrak {sl}_2$ representations of dimension $\ge 11$.^15^ As exemplified by the second relation in (5.18), Pollack relations generically mix contributions of different modular depths $\ge 2$.

### Recursive higher-order computations of $\sigma _w$ and $[z_w,\epsilon _k]$

Based on the vanishing of $[N,\sigma _w]$ in Sect. 7.1 and the $\mathfrak {sl}_2$ prerequisites of Sect. 7.2, we shall now set up the recursive high-order computations of $\sigma _w$ and $[z_w,\epsilon _k]$ in terms of nested brackets of $\epsilon _k^{(j)}$. For this purpose, we parametrize the desired expressions according to modular depth.

#### Definition 7.3.1

Given that $\sigma _w - z_w$ and $[z_w,\epsilon _k]$ both lie in $\mathfrak {u}$ for any odd $w\ge 3$ and even $k \ge 4$ by Theorem 5.4.1 (v) and (vi), we expand

7.8 $$\begin{aligned} \sigma _w&= z_w + \sigma _w^{\{ 1 \}}+ \sigma _w^{\{ 2 \}}+ \sigma _w^{\{ 3 \}}+\cdots + \sigma _w^{\{ w \}} ,\nonumber \\ {[}z_w,\epsilon _k]&= [z_w,\epsilon _k]^{\{ 1 \}}+[z_w,\epsilon _k]^{\{ 2 \}}+ [z_w,\epsilon _k]^{\{ 3 \}} + \cdots + [z_w,\epsilon _k]^{\{ w+1 \}} , \end{aligned}$$

where $\sigma _w^{\{ m\}}$ and $[z_w,\epsilon _k]^{\{ m \}}$ refer to combinations of $[[\ldots [[\epsilon _{k_1}^{(j_1)},\epsilon _{k_2}^{(j_2)}],\epsilon _{k_3}^{(j_3)}],\ldots ], \epsilon _{k_m}^{(j_m)} ]\in \mathfrak {u}$ at modular depth $m=1,2,\ldots ,w+1$. The properties of the arithmetic derivations $z_w \in \textrm{Der}^0\widehat{\textrm{Lie}}[a,b]$ outside $\mathfrak {u}$ can be found in Theorem 5.4.1 (vi) — *a*- and *b*-degree *w* and vanishing commutators $[z_w,\epsilon _0]=[z_w,\epsilon _0^\vee ]=0$.

#### Remark 7.3.2

The maximum modular depth *w* of $\sigma _w$ and $w+1$ of $[z_w,\epsilon _k]$ in (7.8) both follow from the fact that each $\epsilon _m$ with $m\ge 0$ has *b*-degree 1: the *b*-degrees *w* of $\sigma _w$ (see Theorem 5.4.1 (ii)) and $w+1$ of $[z_w,\epsilon _k]$ are incompatible with modular depths $\sigma _w^{\{ m\ge w+1\}}$ and $[z_w,\epsilon _k]^{\{ m\ge w+2 \}}$. The well-known vanishing of $[z_w,\epsilon _k]^{\{ 1 \}}$ [27, 32, 33] follows from the fact that only expression in $\mathfrak {u}$ compatible with its *a*- and *b*-degrees is $\epsilon _{2w+k}^{(w)}$ which violates the lowest-weight-vector property of $z_w$ and $\epsilon _k$.

#### Remark 7.3.3

We recall that generic Pollack relations among $\epsilon _k^{(j)}$ with $k\ge 4$ and $0\le j \le k-2$ in Remark 5.1.6 relate nested brackets of different modular depth $\ge 2$. Accordingly, the individual contributions $\sigma _w^{\{ m\ge 2 \}}$ and $[z_w,\epsilon _k]^{\{ m\ge 2 \}}$ to the right-hand side of (7.8) are usually not well-defined before specifying a scheme of applying those Pollack relations that mix modular depths.^16^ We will specify a choice of $\sigma _{w}^{ \{2\} }$ and $[z_{w},\epsilon _k]^{ \{2\} }$ for all odd $w\ge 3$ in (7.15) and (7.18) below which eliminates some of the ambiguities in $\sigma _{w}^{ \{3\} }$ and $[z_{w},\epsilon _k]^{ \{3\} }$ (those that descend from Pollack relations involving terms of modular depth two). Nevertheless, the recursive relations among $\sigma _{w}^{ \{m\} }$ to be derived below are valid for any scheme of applying Pollack relations that mix different modular depths as long as the same choice is consistently applied to all modular depths $m\ge 2$.

In the companion paper [12], we study uplifts of zeta generators $\sigma _w \rightarrow {\hat{\sigma _w}}$ which no longer act on $\widehat{\textrm{Lie}}[a,b]$ and where the $\epsilon _k^{(j)}$ in their series expansion in $\mathfrak {u}$ are promoted to free-algebra generators $\textrm{e}_k^{(j)}$ with $k\ge 4$ and $0\le j \le k-2$. The expansion of the uplifted ${\hat{\sigma _w}}$ in terms of $\textrm{e}_k^{(j)}$ is determined from considerations of non-holomorphic modular forms and does not share the ambiguities from Pollack relations. Accordingly, the uplifted ${\hat{\sigma _w}}$ induce preferred representations of the $\sigma _{w}^{ \{m\} }$ and $[z_w,\epsilon _k]^{ \{m\} }$ at $m= 2$ and partially at $m=3$ which will be followed in Sect. 7.4.

With the notation of Definition 7.3.1 for the contributions of fixed modular depth *m*, we organize the property $[N,\sigma _w]=0$ as written in (7.2) according to modular depth

7.9 $$\begin{aligned} 0 = [N,\sigma _w]&= {-} [\epsilon _0, \sigma _w^{\{ 1 \}}+\sigma _w^{\{ 2 \}}+\ldots +\sigma _w^{\{ w \}}] \nonumber \\&\quad + \sum _{k=4}^{\infty } (k-1) \textrm{BF}_k \Big ([\epsilon _k,\sigma _w^{\{ 1 \}}] +[\epsilon _k,\sigma _w^{\{ 2 \}}]+\ldots +[\epsilon _k,\sigma _w^{\{ w \}}] \nonumber \\&\quad -[z_w,\epsilon _k]^{\{ 1 \}}-[z_w,\epsilon _k]^{\{ 2 \}}-\ldots -[z_w,\epsilon _k]^{\{ w+1 \}} \Big ) , \end{aligned}$$

where $\textrm{BF}_k :=\frac{ \textrm{B}_k}{k!}$, and we have used $\mathfrak {sl}_2$ invariance $[\epsilon _0,z_w]=0$.

#### Proposition 7.3.4

Upon isolating the contributions to (7.9) at fixed modular depth $m=1,2,\ldots ,{w+1}$, we deduce

7.10 $$\begin{aligned} {[}\epsilon _0,\sigma _w^{\{ m \}}] + \sum _{k=4}^{\infty } (k-1) \textrm{BF}_k [z_w,\epsilon _k]^{\{ m\}} = \sum _{k=4}^{\infty } (k-1) \textrm{BF}_k [\epsilon _k,\sigma _w^{\{ m-1 \}}] . \end{aligned}$$

In particular:

(i) By $\sigma _w^{\{ 0 \}}=0$ and $[z_w,\epsilon _k]^{\{ 1\}}=0$ (see Remark 7.3.2), the $m=1$ instance of (7.10) enforces $[\epsilon _0,\sigma _w^{\{ 1 \}}]=0$. Hence, the only term in $\sigma _w^{\{ 1 \}}$ of modular depth one compatible with the *b*-degree *w* of $\sigma _w$ and (7.10) is the highest-weight vector $\sigma _w^{\{ 1 \}}= - \frac{1}{(w-1)!} \epsilon _{w+1}^{(w-1)}$ identified in Corollary 6.2.3 (i).
(ii) Applying $\textrm{ad}_{\epsilon _0^\vee }$ to both sides of (7.10) implies ($m=2,3,\ldots ,w+1$)
  7.11 $$\begin{aligned} {[}\epsilon _0^\vee ,[\epsilon _0,\sigma _w^{\{ m \}}] ] = \sum _{k=4}^{\infty } (k-1) \textrm{BF}_k [\epsilon _k,[\epsilon _0^\vee ,\sigma _w^{\{ m-1 \}}]] \end{aligned}$$
  since both $z_w$ and $\epsilon _k$ are annihilated by $\textrm{ad}_{\epsilon _0^\vee }$. This is the recursive approach announced earlier on to determine $\sigma _w^{\{ m \}}$ from its precursor at lower modular depth $\sigma _w^{\{ m-1 \}}$ up to the kernel of $\textrm{ad}_{\epsilon _0^\vee } \textrm{ad}_{\epsilon _0}$. Since $\textrm{ad}_{\epsilon _0^\vee }$ is invertible on the image of $\textrm{ad}_{\epsilon _0}$, see (ii) of Corollary 6.3.1 with $Y \in \textrm{ad}_{\epsilon _0^\vee }\mathfrak {u}$ on the right-hand side composed of $[\epsilon _k,[\epsilon _0^\vee ,\sigma _w^{\{ m-1 \}}]]=[\epsilon _0^\vee ,[\epsilon _k,\sigma _w^{\{ m-1 \}}]]$, the only part of $\sigma _w^{\{ m \}}$ which is not yet determined by (7.11) is in the kernel of $\textrm{ad}_{\epsilon _0}$, i.e. a combination of highest-weight vectors of $\mathfrak {sl}_2$. By Theorem 5.4.1 (iv) proven in Sect. 6.3.1, the highest-weight vectors in $\sigma _w$ all occur below or at key degree. In fact, $z_w$ gathers all highest-weight vectors in $\sigma ^{\textrm{key}}_w$ by definition, so $\sigma _w^{\{ m \}}$ at degree 2*w* is free of highest-weight vectors. Hence, the missing information on $\sigma _w^{\{ m \}}$ inaccessible from (7.11) amounts to a finite number of terms at degree $\le 2w-2$.
(iii) By inserting the expression for $\sigma _w^{\{ m \}}$ modulo highest-weight vectors found in (ii) into (7.10) and isolating terms of degree $2w+k$, one can solve for $[z_w,\epsilon _k]^{\{ m\}}$. Note that contributions to $[z_w,\epsilon _k]$ of modular depth *m* determined from $[N,\sigma _w]=0$ only depend on the highest-weight vectors in $\sigma _w$ up to and including modular depth $m-1$.
(iv) Given that $\sigma _w^{\{ 1 \}}= - \frac{1}{(w-1)!} \epsilon _{w+1}^{(w-1)}$, the $m=2$ instances of (7.10) and (7.11) can be written more explicitly as
  7.12 $$\begin{aligned} {[}\epsilon _0,\sigma _w^{\{ 2 \}}] + \sum _{k=4}^{\infty } (k-1) \textrm{BF}_k [z_w,\epsilon _k]^{\{ 2\}} = -\frac{1}{(w-1)!} \sum _{k=4}^{\infty } (k-1) \textrm{BF}_k [\epsilon _k,\epsilon _{w+1}^{(w-1)}] \end{aligned}$$
  and
  7.13 $$\begin{aligned} {[}\epsilon _0^\vee ,[\epsilon _0,\sigma _w^{\{ 2 \}}]] = -\frac{1}{(w-2)!} \sum _{k=4}^{\infty } (k-1) \textrm{BF}_k [\epsilon _k,\epsilon _{w+1}^{(w-2)}] . \end{aligned}$$
  Inverting the operation $\textrm{ad}_{\epsilon _0^\vee } \textrm{ad}_{\epsilon _0}$ determines
  7.14 $$\begin{aligned} \sigma _w^{\{2\}}&= - \sum _{d=5}^w \textrm{BF}_{d-1} s^d(\epsilon _{d-1},\epsilon _{w+1}) - \frac{1}{2} \textrm{BF}_{w+1} s^{w+2}(\epsilon _{w+1},\epsilon _{w+1}) \nonumber \\&\quad + \sum _{k=w+3}^\infty \textrm{BF}_k \sum _{j=0}^{w-2} \frac{(-1)^j \left( {\begin{array}{c}k{-}2\\ j\end{array}}\right) ^{-1} }{j! (w{-}2{-}j)! } \, [ \epsilon _{w+1}^{(w-2-j)} , \epsilon _k^{(j)} ] \ \textrm{mod} \ \textrm{Ker}(\textrm{ad}_{\epsilon _0}) , \end{aligned}$$
  where $\textrm{mod} \ \textrm{Ker}(\textrm{ad}_{\epsilon _0})$ refers to highest-weight vectors to be proposed in (7.18) below. All instances of the brackets $s^d(\epsilon _{k_1},\epsilon _{k_2})$ defined by (7.6) that occur in (7.14) have $d> \textrm{min}(k_1,k_2)$ and are therefore not highest-weight vectors. Upon insertion of (7.14) into (7.12) and isolating terms of degree $2w+k$, we reproduce the closed-form expression at modular depth two known form [27]
  7.15 $$\begin{aligned} {[}z_w,\epsilon _k]^{\{ 2\}} = \frac{\textrm{BF}_{w+k-1}}{ \textrm{BF}_{k} } t^{w+1}(\epsilon _{w+1},\epsilon _{w+k-1}) . \end{aligned}$$
(v) The instance of (7.10) at the maximum value $m=w+1$ simplifies to
  7.16 $$\begin{aligned} \sum _{k=4}^{\infty } (k-1) \textrm{BF}_k [z_w,\epsilon _k]^{\{ w+1\}} = \sum _{k=4}^{\infty } (k-1) \textrm{BF}_k [\epsilon _k,\sigma _w^{\{ w \}}] \end{aligned}$$
  by $\sigma _w^{\{ w+1\}}=0$. Hence, the contribution to $[z_w,\epsilon _k]$ of highest modular depth $w+1$ can simply be determined from the highest-modular depth terms in $\sigma _w$ by isolating the parts of degree $2w+k$ in (7.16). Validity of (7.10) at $m=1,2,\ldots ,w+1$ — finitely many steps in the recursion in the modular depth — is sufficient for $[N,\sigma _w]=0$, see (7.9).

Note that parts (ii) and (iii) of Proposition 7.3.4 can also be unified by the decomposition of $[\epsilon _k,\sigma _w^{\{ m-1 \}}]$ on the right-hand side of (7.10) into the image of $\textrm{ad}_{\epsilon _0}$ and the kernel of $\textrm{ad}_{\epsilon _0^\vee }$,

7.17 $$\begin{aligned} {[}\epsilon _0,\sigma _w^{\{ m \}}]&= \sum _{k=4}^{\infty } (k-1) \textrm{BF}_k [\epsilon _k,\sigma _w^{\{ m-1 \}}] \, \big |_{\textrm{Im}(\textrm{ad}_{\epsilon _0})} , \nonumber \\ \sum _{k=4}^{\infty } (k-1) \textrm{BF}_k [z_w,\epsilon _k]^{\{ m\}}&= \sum _{k=4}^{\infty } (k-1) \textrm{BF}_k [\epsilon _k,\sigma _w^{\{ m-1 \}}] \, \big |_{\textrm{Ker}(\textrm{ad}_{\epsilon _0^\vee })} . \end{aligned}$$

This decomposition is unique since $\textrm{Ker}(\textrm{ad}_{\epsilon _0^\vee })$ projects the individual terms of $[\epsilon _k,\sigma _w^{\{ m-1 \}}]$ to lowest-weight vectors which do not occur in the image of $\textrm{ad}_{\epsilon _0}$.

### Applying the recursion for $\sigma _w^{\{ m\}}$ and $[z_w,\epsilon _k]^{\{ m\}}$

In this section, we gather explicit results for zeta generators and commutators $[z_w,\epsilon _k]$ at modular depth $2\le m \le 4$ that go considerably beyond the state of the art and found fruitful applications in the construction of non-holomorphic modular forms [12].

#### Zeta generators at modular depth two

The relation (7.13) for the modular-depth-two contributions $\sigma _w^{\{2\}}$ to the zeta generators determines the infinite series of terms in (7.14) that are not highest-weight vectors. We shall now augment these terms by a conjectural closed formula for the highest-weight vectors in $\sigma _w^{\{2\}}$ given by the first line of

7.18 $$\begin{aligned} \sigma _w^{\{2\}}&= -\frac{1}{2} \sum _{d=3}^{w-2} \frac{ \textrm{BF}_{d-1} }{\textrm{BF}_{w-d+2} } \sum _{k=d+1}^{w-1} \textrm{BF}_{k-d+1} \textrm{BF}_{w-k+1} s^d(\epsilon _k,\epsilon _{w-k+d}) \nonumber \\&\quad - \sum _{d=5}^w \textrm{BF}_{d-1} s^d(\epsilon _{d-1},\epsilon _{w+1}) - \frac{1}{2} \textrm{BF}_{w+1} s^{w+2}(\epsilon _{w+1},\epsilon _{w+1}) \nonumber \\&\quad + \sum _{k=w+3}^\infty \textrm{BF}_k \sum _{j=0}^{w-2} \frac{(-1)^j \left( {\begin{array}{c}k{-}2\\ j\end{array}}\right) ^{-1} }{j! (w{-}2{-}j)! } \, [ \epsilon _{w+1}^{(w-2-j)} , \epsilon _k^{(j)} ] . \end{aligned}$$

This conjecture for the complete parts $\sigma _w^{\{2\}}$ of modular depth two is readily checked to reproduce the terms $[\epsilon _{k_1}^{(j_1)},\epsilon _{k_2}^{(j_2)}]$ in the examples (5.46) to (5.50) at $w\le 9$. The first line of (7.18) gathers highest-weight vectors such as $-\frac{5 }{48} [\epsilon _4^{(1)},\epsilon _4^{(2)}]$ in $\sigma _5^{\{2\}}$ and $\frac{7 }{1152} ( [\epsilon _4^{(2)},\epsilon _6^{(3)}] -[\epsilon _4^{(1)},\epsilon _6^{(4)}])+\frac{1}{13{,}824}([\epsilon _6^{(1)},\epsilon _6^{(4)}] - [\epsilon _6^{(2)},\epsilon _6^{(3)}])$ in $\sigma _7^{\{2\}}$^17^ which have been tested for all cases of degree $\le 22$ and are in general conjectural. Note that the highest-weight-vector contributions to $\sigma _w^{\{2\}}$ in the first line of (7.18) are in one-to-one correspondence with the $\tau \rightarrow i\infty$ asymptotics of the generalized Eisenstein series $\textrm{F}^{+(s)}_{m,k}$ in [93, 94] at $m+k+s=w+1$ upon assembling their iterated-integral representations from the generating series of [12].

The images of the terms $s^d(\epsilon _{k_1},\epsilon _{k_2})$ under the switch operation in Definition 5.1.4 have *b*-degree or depth *d*, and their $d=3$ instances line up with Brown’s general formula for the depth-three contributions to $\tau _w$ [33]. However, the choice of $\tau _{w\ge 11}$ in the reference does not match the *canonical* zeta generators in this work since redefinitions via nested brackets of $\tau _v$ at $v<w$ have been used in [33] to remove contributions of modular depth and *b*-degree three. The second and third line of (7.18) are rigorously derived by solving (7.13) and, together with the conjectural highest-weight vectors at depth $d\ge 5$ in the first line, furnish a partial generalization of Brown’s result beyond depth three: On the one hand, (7.18) is claimed to capture all contributions $[\epsilon _{k_1}^{(j_1)},\epsilon _{k_2}^{(j_2)}]$ to $\sigma _w$, regardless of their values of $j_1,j_2,k_1,k_2$ or depth in the sense of [33]. On the other hand, terms in $\sigma _w$ at depth or *b*-degree *d* involve contributions of modular depth up to and including *d*, and closed formulae for $\sigma _w^{\{ m\ge 3\}}$ akin to (7.18) are currently out of reach.

Note that, following the comments below (7.6), the $s^{d}(\epsilon _{k_1},\epsilon _{k_2})$ in the second line of (7.18) have $d>\textrm{min}(k_1,k_2)$ and are therefore not highest-weight vectors. Moreover, the expression (7.18) for contributions to $\sigma _w$ of modular depth two can be rewritten in a variety of ways via Pollack relations among $\epsilon _k^{(j)}$, see Remark 7.3.3. Hence, the closed formula (7.18) for $\sigma _w^{\{ 2 \}}$ realizes a specific choice of distributing terms between different modular depths.

#### Commutators of arithmetic derivations at modular depth three

By Proposition 7.3.4 (iii), the highest-weight vectors in $\sigma _w$ at modular depth *m* determine the contributions to the brackets $[z_w,\epsilon _k]$ at modular depth $m+1$ via (7.10). The conjectural expressions (7.18) for $\sigma _w^{\{2\}}$ therefore translate into expressions for $[z_w,\epsilon _k]^{\{3\}}$ that generalize the simple closed formula (7.15) for terms of modular depth two.

Contributions to $[z_3,\epsilon _k]$ and $[z_5,\epsilon _k]$ at modular depth $\ge 3$ and low values of *k* have been firstly reported in [32] and the ancillary files of [11], respectively. Moreover, the combinatorial tools developed in [32] can be used to determine more general expressions for $[z_w,\epsilon _k]$. Our conjecture (7.18) for $\sigma _w^{\{2\}}$ gives access to arbitrary $[z_w,\epsilon _k]^{\{3\}}$, but the expressions resulting from the representation-theoretic manipulations become increasingly unwieldy with growing *w*. Hence, we content ourselves to giving the following two infinite families of commutation relations beyond the state of the art with arbitrary even $k\ge 4$ (see (7.5) for the iteration of the projector $t^d$ to lowest-weight vectors),

7.19 $$\begin{aligned} {[}z_3,\epsilon _k]^{\{3\}}&= \frac{3 \textrm{BF}_4 \textrm{BF}_{k-2} }{\textrm{BF}_k} \bigg \{ {-} \frac{(k{-}3) }{(k{-}1)} \, t^2 (\epsilon _4, t^3(\epsilon _4,\epsilon _{k-2})) + \frac{(k{-}2) }{k} \, t^3 (\epsilon _4, t^2(\epsilon _4,\epsilon _{k-2})) \bigg \} \nonumber \\&\quad + \frac{1}{(k{-}1) \textrm{BF}_k} \sum _{\ell =6}^{k-4} (\ell {-}1) \textrm{BF}_\ell \textrm{BF}_{k+2-\ell } \nonumber \\&\quad \times \bigg \{ {-} \frac{2(k{-}\ell {+}1) }{(k{-}\ell {+}2) } \, t^2 (\epsilon _\ell , t^3(\epsilon _4,\epsilon _{k+2-\ell })) +\frac{\ell {-}2 }{k} \, t^3(\epsilon _\ell , t^2(\epsilon _4,\epsilon _{k+2-\ell })) \bigg \} \end{aligned}$$

and

7.20 $$\begin{aligned} {[}z_5,\epsilon _k]^{\{3\}}&= \frac{ \textrm{BF}_{k+2} \textrm{BF}_2^3 }{2 \textrm{BF}_4 \textrm{BF}_k } \, t^4(\epsilon _{k+2},t^3(\epsilon _4,\epsilon _4)) \nonumber \\&\quad + \frac{5 \textrm{BF}_6 \textrm{BF}_{k-2} }{\textrm{BF}_k} \bigg \{ {-}\frac{ (k{-}5)}{(k{-}1)} \, t^2 (\epsilon _6, t^5(\epsilon _6,\epsilon _{k-2}))\nonumber \\&\quad +\frac{2(k{-}3)(k{-}4) }{k(k{-}1)} \, t^3 (\epsilon _6, t^4(\epsilon _6,\epsilon _{k-2})) \nonumber \\&\quad - \frac{2 (k{-}2)(k{-}3) }{k (k{+}1)} \, t^4 (\epsilon _6, t^3(\epsilon _6,\epsilon _{k-2})) +\frac{ (k{-}2) }{(k{+}2)} \, t^5 (\epsilon _6, t^2(\epsilon _6,\epsilon _{k-2})) \bigg \} \nonumber \\&\quad + \textrm{BF}_4 \bigg \{ {-}\frac{12(k{-}3) }{k(k{-}1)}\, t^2(\epsilon _4, t^5(\epsilon _6,\epsilon _k)) +\frac{ 36 (k{-}2) }{k^2(k{+}1)} \, t^3(\epsilon _4, t^4(\epsilon _6,\epsilon _k)) \nonumber \\&\quad - \frac{24}{k(k{+}1)(k{+}2)} \, t^4(\epsilon _4, t^3(\epsilon _6,\epsilon _k)) -\frac{9(k{-}2) }{5k} \, t^3(\epsilon _k, t^4(\epsilon _4,\epsilon _6)) \nonumber \\&\quad - \frac{2(k{-}2)(k{-}3) }{k(k{+}1)} \, t^4(\epsilon _k, t^3(\epsilon _4,\epsilon _6)) - \frac{(k{-}2)(k{-}3)(k{-}4) }{k(k{+}1)(k{+}2) } t^5(\epsilon _k, t^2(\epsilon _4,\epsilon _6)) \bigg \} \nonumber \\&\quad + \frac{1}{(k{-}1) \textrm{BF}_k} \sum _{\ell =8}^{k-4} (\ell {-}1) \textrm{BF}_\ell \textrm{BF}_{k+4-\ell } \bigg \{ {-}\frac{4(k{-}\ell {+}1) }{(k{-}\ell {+}4)} \, t^2(\epsilon _\ell , t^5(\epsilon _6,\epsilon _{k+4-\ell })) \nonumber \\&\quad + \frac{6(\ell {-}2) (k{-}\ell {+}2)(k{-}\ell {+}3) }{k(k{-}\ell {+}4)(k{-}\ell {+}5) } \, t^3(\epsilon _\ell , t^4(\epsilon _6,\epsilon _{k+4-\ell })) \nonumber \\&\quad -\frac{4 (\ell {-}3)(\ell {-}2) (k{-}\ell {+}3) }{k(k{+}1)(k{-}\ell {+}6) } \, t^4(\epsilon _\ell , t^3(\epsilon _6,\epsilon _{k+4-\ell })) \nonumber \\&\quad +\frac{(\ell {-}2)(\ell {-}3)(\ell {-}4) }{k(k{+}1)(k{+}2) } \, t^5(\epsilon _\ell , t^2(\epsilon _6,\epsilon _{k+4-\ell })) \bigg \} . \end{aligned}$$

The remaining brackets $[z_w,\epsilon _k]^{\{3\}}$ at degree $\le 20$ are given by

7.21 $$\begin{aligned} {[}z_7,\epsilon _4]^{\{3\}}&= \frac{ \textrm{BF}_8 \textrm{BF}_2^2 }{\textrm{BF}_6} \, t^6(\epsilon _8,t^3(\epsilon _4,\epsilon _6)) + \frac{ \textrm{BF}_6 \textrm{BF}_2^2 }{2 \textrm{BF}_4} \, t^4(\epsilon _6,t^5(\epsilon _6,\epsilon _6)) \nonumber \\&\quad - \textrm{BF}_6 \bigg \{ \frac{15}{14} \, t^3(\epsilon _4, t^6(\epsilon _6,\epsilon _8)) + \frac{5}{14} \, t^4(\epsilon _4, t^5(\epsilon _6,\epsilon _8)) \nonumber \\&\quad +\frac{5}{7} \, t^5(\epsilon _4, t^4(\epsilon _6,\epsilon _8)) +\frac{3}{28} \,t^6(\epsilon _4, t^3(\epsilon _6,\epsilon _8)) \bigg \} , \nonumber \\ {[}z_7,\epsilon _6]^{\{3\}}&= \frac{ \textrm{BF}_{10} \textrm{BF}_4 \textrm{BF}_2^2 }{\textrm{BF}_6^2} \, t^6(\epsilon _{10},t^3(\epsilon _4,\epsilon _6)) + \frac{ \textrm{BF}_8 \textrm{BF}_2^2 }{2 \textrm{BF}_6} \, t^4(\epsilon _8,t^5(\epsilon _6,\epsilon _6)) \nonumber \\&\quad - \frac{ \textrm{BF}_4 \textrm{BF}_8 }{\textrm{BF}_6} \bigg \{ \frac{5}{2}\, t^5(\epsilon _8,t^4(\epsilon _4,\epsilon _8)) + \frac{7}{2}\, t^6(\epsilon _8,t^3(\epsilon _4,\epsilon _8)) +\frac{14}{5}\, t^7(\epsilon _8,t^2(\epsilon _4,\epsilon _8)) \bigg \} \nonumber \\&\quad - \textrm{BF}_6 \bigg \{ \frac{10}{7} \, t^3(\epsilon _6,t^6(\epsilon _6,\epsilon _8)) + \frac{50}{49} \, t^4(\epsilon _6,t^5(\epsilon _6,\epsilon _8)) \nonumber \\&\quad +\frac{25}{84} \, t^5(\epsilon _6,t^4(\epsilon _6,\epsilon _8)) +\frac{1}{42} \, t^6(\epsilon _6,t^3(\epsilon _6,\epsilon _8)) \bigg \} . \end{aligned}$$

#### Exact results for $\sigma _3$ and $z_3$

Once the complete set of highest-weight vectors for a given $\sigma _w$ is available, then the recursion (7.10) determines all-degree expressions for both $\sigma _w^{\{ 2 \}},\sigma _w^{\{ 3 \}},\ldots ,\sigma _w^{\{ w \}}$ and $[z_w,\epsilon _k]^{\{2\}},[z_w,\epsilon _k]^{\{3\}},$ $\ldots , [z_w,\epsilon _k]^{\{w+1\}}$. With the highest-weight vectors for $\sigma _3,\sigma _5,\sigma _7$ noted in Sect. 7.1, there is no obstruction to algorithmically assembling the exact results for the expansions of $\sigma _w$ and $[z_w,\epsilon _k]$ at $w\le 7$.

We shall here display the exact results for $\sigma _3$ and $[z_3,\epsilon _k]$ which terminate with modular depth three and four, respectively. The all-order expansion of $\sigma _3$ is given by,

7.22 $$\begin{aligned} \sigma _3&= - \frac{1}{2} \epsilon _4^{(2)} + z_3 + \frac{1}{480} [\epsilon _4,\epsilon _4^{(1)}] + \sum _{k=6}^\infty \textrm{BF}_k \bigg ( [\epsilon _4^{(1)} , \epsilon _k ] -\frac{ [ \epsilon _4 , \epsilon _k^{(1)} ] }{k{-}2} \bigg ) \nonumber \\&\quad + \sum _{m=4}^{\infty } \sum _{r=6}^{\infty } \frac{(m{-}1)\textrm{BF}_m \textrm{BF}_r}{m{+}r{-}2} \big [ \epsilon _m ,[\epsilon _4, \epsilon _r]\big ] , \end{aligned}$$

where the second line is obtained by solving (7.11) at $m=w=3$ for $\sigma _3^{\{3\}}$ with the expression for $\sigma _3^{\{2\}}$ determined by the first line. The action of the arithmetic part $z_3$ on *a*, *b* can be found in (5.47). The expression for $[z_3,\epsilon _{k}]$ resulting from $[N,\sigma _3]=0$ can be assembled by combining $[z_3,\epsilon _k]^{\{2\}} = \frac{\textrm{BF}_{k+2}}{ \textrm{BF}_{k} } t^{4}(\epsilon _{4},\epsilon _{k+2})$ from (7.15) with the expression (7.19) for $[z_3,\epsilon _k]^{\{3\}}$ and the degree-$(2w+k)$ parts of

7.23 $$\begin{aligned}&\sum _{k=4}^{\infty }(k-1)\textrm{BF}_k [z_3,\epsilon _k]^{\{4\}} = \sum _{k=4}^{\infty }(k-1)\textrm{BF}_k \sum _{m=4}^{\infty } \sum _{r=6}^{\infty } \frac{ (m-1) \textrm{BF}_m \textrm{BF}_r }{(m + r - 2) } [\epsilon _k,[\epsilon _m,[\epsilon _4, \epsilon _r]]] \end{aligned}$$

which follows from (7.16) at $w=3$. The lowest-degree examples of $[z_3,\epsilon _k]^{\{4\}}$ occur in

$$\begin{aligned} {[}z_3,\epsilon _{12}]&= \frac{\textrm{BF}_{14}}{\textrm{BF}_{12}} t^4(\epsilon _{4}, \epsilon _{14}) +\frac{ \textrm{BF}_4 \textrm{BF}_{10} }{\textrm{BF}_{12}} \bigg \{ {-}\frac{27}{11} t^2(\epsilon _{4}, t^3(\epsilon _4,\epsilon _{10})) + \frac{ 5}{2} t^3(\epsilon _{4}, t^2(\epsilon _4,\epsilon _{10})) \bigg \} \\&\quad + \frac{ \textrm{BF}_6 \textrm{BF}_8 }{\textrm{BF}_{12}} \bigg \{ {-}\frac{35}{44} t^2(\epsilon _{6}, t^3(\epsilon _4,\epsilon _{8})) +\frac{5}{33} t^3(\epsilon _{6}, t^2(\epsilon _4,\epsilon _{8})) \\&\quad - \frac{35}{33} t^2(\epsilon _{8}, t^3(\epsilon _4,\epsilon _{6})) +\frac{7}{22} t^3(\epsilon _{8}, t^2(\epsilon _4,\epsilon _{6})) \bigg \} \\  &\quad +\frac{ 9 \textrm{BF}_{4}^2 \textrm{BF}_{6} }{88 \textrm{BF}_{12} } [\epsilon _{4}, [\epsilon _{4}, [\epsilon _{4}, \epsilon _{6} ] ] ] \end{aligned}$$

as well as

$$\begin{aligned} {[}z_3,\epsilon _{14}]&= \frac{ \textrm{BF}_{16}}{\textrm{BF}_{14}} t^4(\epsilon _4,\epsilon _{16}) + \frac{ \textrm{BF}_{4} \textrm{BF}_{12}}{\textrm{BF}_{14}} \bigg \{ \frac{18}{7} t^3( \epsilon _4, t^2(\epsilon _4,\epsilon _{12})) - \frac{33}{13}t^2( \epsilon _4, t^3(\epsilon _4,\epsilon _{12})) \bigg \} \\&\quad + \frac{ \textrm{BF}_{6}\textrm{BF}_{10}}{\textrm{BF}_{14}} \bigg \{ \frac{10}{91} t^3( \epsilon _6, t^2(\epsilon _4,\epsilon _{10})) - \frac{9}{13} t^2( \epsilon _6, t^3(\epsilon _4,\epsilon _{10})) \\&\quad + \frac{36}{91} t^3( \epsilon _{10}, t^2(\epsilon _4,\epsilon _{6})) - \frac{15}{13} t^2( \epsilon _{10}, t^3(\epsilon _4,\epsilon _{6})) \bigg \} \\&\quad + \frac{ \textrm{BF}_{8}^2}{ \textrm{BF}_{14} } \bigg \{ \frac{3}{13} t^3( \epsilon _8, t^2(\epsilon _4,\epsilon _{8})) - \frac{49}{52} t^2( \epsilon _8, t^3(\epsilon _4,\epsilon _{8})) \bigg \} \\&\quad + \frac{ 9 \textrm{BF}_{4}^2 \textrm{BF}_{8} }{ 130 \textrm{BF}_{14}} [\epsilon _{4}, [\epsilon _{4}, [\epsilon _{4}, \epsilon _{8} ] ] ] + \frac{ 27 \textrm{BF}_{4} \textrm{BF}_{6}^2 }{ 104 \textrm{BF}_{14}} [\epsilon _{4}, [\epsilon _{6}, [\epsilon _{4}, \epsilon _{6} ] ] ] , \end{aligned}$$

also see appendix E.1 of [12] for $[z_3,\epsilon _{k}]$ at $k=4,6,8,10$.

#### Highest-weight vectors at modular depth three

While a comprehensive study of highest-weight vector contributions to $\sigma _w^{\{m\ge 3\}}$ is left for the future, their instances at $w\le 11$ are accessible from the ancillary files of [12]. The simplest highest-weight vector at modular depth three occurs in the expansion (5.49) of $\sigma _7$ at degree 12 and can be compactly written as $- \frac{661 }{14400} s^{3}(\epsilon _ 4, t^{3}(\epsilon _4, \epsilon _4))$ through the combination (7.7) of $s^d$ and $t^d$ operations. This shorthand also streamlines the expansions of $\sigma _9,\sigma _{11}$ to

7.24 $$\begin{aligned} \sigma _9&= -\frac{\epsilon _{10}^{(8)}}{ 8! } + \frac{5 s^3(\epsilon _{4},\epsilon _{8})}{18} + \frac{7 s^3(\epsilon _{6},\epsilon _{6}) }{72} + \frac{s^5(\epsilon _{4},\epsilon _{10})}{720} - \frac{7 s^5(\epsilon _{6},\epsilon _{8})}{1440} \nonumber \\&\quad + \frac{34921 s^{2}(\epsilon _ 4, t^{4}(\epsilon _4, \epsilon _6))}{1134000} + \frac{ 2587 s^{3}(\epsilon _ 4, t^{3}(\epsilon _4, \epsilon _6)) }{37800} - \frac{ 529 s^{4}(\epsilon _ 4, t^{2}(\epsilon _4, \epsilon _6)) }{14400} \nonumber \\  &\quad - \frac{s^7(\epsilon _{6},\epsilon _{10})}{30240} + \frac{s^7(\epsilon _{8},\epsilon _{8})}{12096} +\frac{ s^{5}(\epsilon _ 4, t^{3}(\epsilon _4, \epsilon _8)) }{2592} +\frac{ 7s^{5}(\epsilon _ 4, t^{3}(\epsilon _6, \epsilon _6)) }{51840 } \nonumber \\  &\quad - \frac{ 34921 s^{4}(\epsilon _ 6, t^{4}(\epsilon _6, \epsilon _4)) }{47628000} - \frac{ 2587 s^{5}(\epsilon _ 6, t^{3}(\epsilon _6, \epsilon _4)) }{1587600} + \frac{ 529 s^{6}(\epsilon _ 6, t^{2}(\epsilon _6, \epsilon _4)) }{604800 } \nonumber \\&\quad \frac{149 s^{3}(\epsilon _4,t^{3}(\epsilon _4,t^{3}(\epsilon _4,\epsilon _4)))}{13824}-\frac{149s^{4}(\epsilon _4,t^{2}(\epsilon _4,t^{3}(\epsilon _4,\epsilon _4)))}{69120} +\ldots \nonumber \\ \sigma _{11}&= -\frac{\epsilon _{12}^{(10)} }{ 10! } + \frac{11 s^3(\epsilon _{4},\epsilon _{10})}{40} + \frac{11 s^3(\epsilon _{6},\epsilon _{8}) }{60} + \frac{ 242407 s^{2}(\epsilon _ 4, t^{2}(\epsilon _4, \epsilon _6))}{14735232} + \frac{s^5(\epsilon _{4},\epsilon _{12} )}{720} \nonumber \\&\quad -\frac{s^5(\epsilon _{6},\epsilon _{10})}{216} - \frac{7 s^5(\epsilon _{8},\epsilon _{8})}{4320} +\frac{11090423 s^{2}(\epsilon _ 4, t^{4}(\epsilon _4, \epsilon _8)) }{309439872} + \frac{ 3197 s^{3}(\epsilon _ 4, t^{3}(\epsilon _4, \epsilon _8)) }{57600} \nonumber \\&\quad - \frac{ 2983 s^{4}(\epsilon _ 4, t^{2}(\epsilon _4, \epsilon _8)) }{86400} + \frac{ 148753 s^{3}(\epsilon _ 4, t^{3}(\epsilon _6, \epsilon _6)) }{7367616} + \frac{ 490853 s^{3}(\epsilon _ 6, t^{3}(\epsilon _6, \epsilon _4)) }{17191104} \nonumber \\&\quad + \frac{ 156805 s^{4}(\epsilon _ 6, t^{2}(\epsilon _6, \epsilon _4)) }{14735232} + c \, s^{2}(\epsilon _ 4, t^{2}(\epsilon _4, t^{3} (\epsilon _4,\epsilon _4))) + \ldots , \end{aligned}$$

where the ellipsis refers to all contributions of degree $\ge 18$, and the coefficient $c\in {\mathbb {Q}}$ of the first modular-depth-four contribution to $\sigma _{11}$ in the last line has not yet been computed. It is, however, a highest-weight vector and entirely fixed by our construction. Note that the $s^{d_2}(\epsilon _ {k_3}, t^{d_1}(\epsilon _{k_1}, \epsilon _{k_2}))$ only furnish highest-weight vectors if $d_2\le \textrm{min}(k_3,r)$, where $r=k_1+k_2-2d_1+2$. Accordingly, all the terms $s^{d_2}(\epsilon _ {k_3}, t^{d_1}(\epsilon _{k_1}, \epsilon _{k_2}))$ of modular depth three in (7.24) are highest-weight vectors with the exception of the contributions $s^{5}(\epsilon _ 4, t^{3}(\epsilon _4, \epsilon _8))$ and $s^{5}(\epsilon _ 4, t^{3}(\epsilon _6, \epsilon _6))$ to $\sigma _9$. The ancillary files of [12] provide all contributions to $\sigma _w^{\{m\le 3\}}$ at degree $\le 20$ in machine-readable form which determines all the highest-weight vectors of $\sigma _9^{\{3 \}}$ and $\sigma _{11}^{\{3 \}}$.

## Figures and Tables

**Fig. 3:**
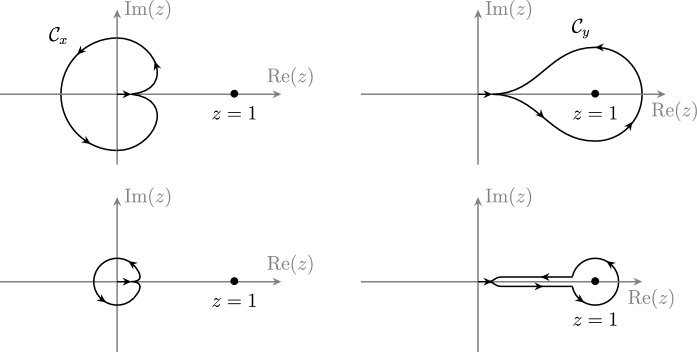
The loops ${{\mathcal {C}}}_x$ and ${{\mathcal {C}}}_y$ around ${\text {z}}=0$ and ${\text {z}}=1$ anchored at the origin (upper half) and their homotopy deformation to infinitesimal circles along with straight paths between zero and one in case of ${{\mathcal {C}}}_y$ (lower half) [81]. Strictly speaking, all the contours start and end at the tangential base point from 0 to 1 as indicated by the arrows at the origin pointing along the positive real axis. The straight line portions of the path in the lower-right panel should be viewed as running along the real axis between 0 and 1; they have been slightly separated for visual convenience

**Fig. 4:**
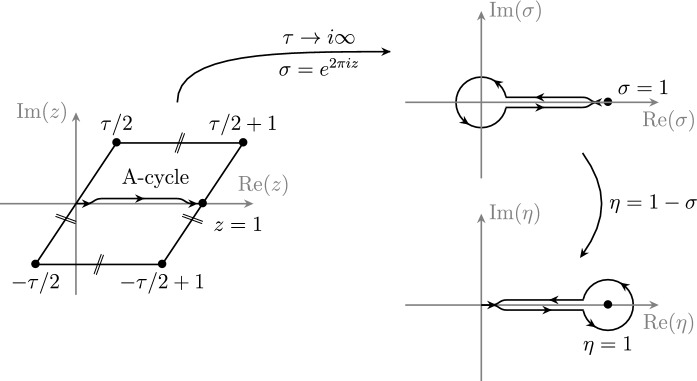
The degeneration $\tau \rightarrow i \infty$ of the torus with coordinate *z* (left panel) yields a nodal sphere, where the image of the A-cycle connecting $z=0$ with $z=1$ is drawn in two different coordinates $\sigma$ and $\eta$ (right panel). The image of the A-cycle in the $\eta$ coordinate (lower-right panel) matches the deformation of the loop ${{\mathcal {C}}}_y$ around $z=1$ in Fig. 3. Similar to Fig. 3, the straight line portions of all the paths should be viewed as running along the real axis between 0 and 1; they have been slightly separated for visual convenience

## Data Availability

The ancillary files in the arXiv and journal submissions of this work provide supplemental data concerning the canonical polynomials $g_w$ and the arithmetic derivations $z_w$ mentioned in the introduction.

## Appendix A. Deriving the Topological Map from the Sphere to the Torus

The goal of this appendix is to derive the explicit form of the map (5.29) and (5.30) between the generators *x*, *y* and *a*, *b* of the fundamental groups in genus zero and genus one, respectively. Our derivation will be based on a formulation of the zeta generators in terms of Knizhnik–Zamolodchikov (KZ) connections in genus zero and Knizhnik–Zamolodchikov–Bernard (KZB) connections in genus one. The form of the KZ connection obtained from the degeneration limit of the KZB connection then relates the generators *x*, *y* of the fundamental group of the thrice punctured sphere to the generators *a*, *b* of the fundamental group of the once-punctured torus.

### Zeta generators in terms of the KZ connection

In this appendix we assume the conjecture that the surjection from motivic to real MZVs is an isomorphism, and thus identify the motivic version $\Phi ^{\mathfrak {m}}(x,y)$ of the modified Drinfeld associator with $\Phi (x,y)$ as defined in (3.42). We will systematically assume that $\Phi (x,y)$ is written in the semi-canonical basis defined in Sect. 3.5, and use the notation

A.1 $$\begin{aligned} g_w=\Phi (x,y)|_{\zeta _w} \end{aligned}$$

for the canonical polynomial $g_w$ that then appears in $\Phi$ with coefficient $\zeta _w$ for odd $w\ge 3$ (see Definition 3.3.5). The power series $\Phi (x,y)$ in (3.42) can be obtained as the path-ordered exponential of the modified KZ connection *J* defined by^18^

A.2 $$\begin{aligned} J(x,y;z)&:=\, \bigg ( \frac{x}{z} + \frac{y}{1-z} \bigg )dz , \ \ \ \ z \in {\mathbb {C}} \setminus \{0,1 \} , \nonumber \\ \Phi (x,y)&= \textrm{Pexp} \bigg ( \int ^1_0 J(x,y;z) \bigg ) , \nonumber \\ g_w(x,y)&= \Phi (x,y) \, \big |_{\zeta _w} = \textrm{Pexp} \bigg ( \int ^1_0 \bigg [ \frac{x}{z} + \frac{y}{1-z} \bigg ]dz \bigg ) \, \big |_{\zeta _w} , \end{aligned}$$

where the iterated integration is taken over the simplex $0< z_1< \cdots< z_r< 1$, and the convention for expanding path-ordered exponentials is

A.3 $$\begin{aligned} \textrm{Pexp}\Biggl (\int _0^1 J(z)\Biggr )=1+\sum _{r= 1}^{\infty } \int _0^1 J(z_r) \int _0^{z_r} J(z_{r-1}) \cdots \int _0^{z_3} J(z_2) \int _0^{z_2} J(z_1) . \end{aligned}$$

The endpoint divergences in (A.3) are understood to be regularized by passing to shuffle-regularized versions (2.3) of the MZVs in the expansion of $\Phi (x,y)$.

The zeta generators in genus zero are given by the Ihara derivations $D_{g_w}$ associated to the polynomials $g_w$, which act on the free Lie algebra $\textrm{Lie}[x,y]$ via

A.4 $$\begin{aligned} D_{g_w}(x) = 0 , \ \ \ \ D_{g_w}(y) = [y,g_w(x,y)]\, ; \end{aligned}$$

they can be interpreted as the coefficient of $\zeta _w$ in the holonomies of *J*(*x*, *y*; *z*) w.r.t. the loops around $z=0$ and $z=1$, respectively. More specifically, (A.4) extracts the *linearized* monodromy of the loops ${{\mathcal {C}}}_x$ and ${{\mathcal {C}}}_y$ around $z=0$ and $z=1$ anchored at the tangential base point from 0 to 1 as drawn in Fig. 3, where only the first power of $2\pi i$ is retained:

A.5 $$\begin{aligned} D_{g_w}(x)&= - \textrm{Pexp} \bigg ( \int _{{{\mathcal {C}}}_x} J(x,y;z) \bigg ) \, \big |_{2\pi i \zeta _w} , \nonumber \\ D_{g_w}(y)&= - \textrm{Pexp} \bigg ( \int _{{{\mathcal {C}}}_y} J(x,y;z) \bigg ) \, \big |_{2\pi i \zeta _w} \end{aligned}$$

Equivalence to (A.4) can be seen as follows:

- The path-ordered exponentials of *J*(*x*, *y*; *z*) associated with the infinitesimal circles around 0 and 1 in counter-clockwise orientation are given by $e^{2 \pi i x}$ and $e^{-2 \pi i y}$, respectively.
- Since ${{\mathcal {C}}}_x$ is homotopic to an infinitesimal circle around $z=0$, we have
  A.6 $$\begin{aligned} \textrm{Pexp} \bigg ( \int _{{{\mathcal {C}}}_x} J(x,y;z) \bigg ) = e^{2 \pi i x} \end{aligned}$$
  which does not contain any odd Riemann zeta values, thereby reproducing $D_{g_w}(x) =0$.
- The path ${{\mathcal {C}}}_y$ is homotopic to the composition of the path (0, 1) followed by an infinitesimal circle around $z=1$ and the inverse path (1, 0) as seen in the lower-right panel of Fig. 3. Hence, the path-ordered exponential can be decomposed into
  A.7 $$\begin{aligned} \textrm{Pexp} \bigg ( \int _{{{\mathcal {C}}}_y} J(x,y;z) \bigg ) = \Phi (x,y)^{-1} e^{-2 \pi i y} \Phi (x,y) . \end{aligned}$$
  By the conventions (A.3) for path-ordered exponentials, the last segment (1, 0) of the deformed path ${{\mathcal {C}}}_y$ translates into the leftmost factor $\Phi (x,y)^{-1}$.
- Extracting the coefficient of $\zeta _w$ from (A.7) leads to
  A.8 $$\begin{aligned} \textrm{Pexp} \bigg ( \int _{{{\mathcal {C}}}_y} J(x,y;z) \bigg ) \, \big |_{\zeta _w }= e^{-2 \pi i y} g_w(x,y) - g_w(x,y) e^{-2 \pi i y} \end{aligned}$$
  which upon linearization in $2\pi i$ reduces to $-2\pi i [y, g_w(x,y)]$ and reproduces the action of $D_{g_w}$ on *y* in (A.4).

We emphasize that it will be the formulation (A.5) of zeta generators in terms of linearized monodromies which generalizes from genus zero to genus one.

### Degenerating the KZB connection

In the same way as the (modified) KZ connection (A.2) can be used to generate multiple polylogarithms in genus zero, the Brown–Levin formulation of elliptic polylogarithms in genus one [95] is based on the KZB connection

A.9 $$\begin{aligned} J_{\textrm{KZB}}(A,B;z|\tau )&:=\, \textrm{ad}_B F(z,\textrm{ad}_B| \tau )A \,dz , \ \ \ \ F(z,\alpha | \tau ) :=\frac{\theta '_1(0|\tau ) \theta _1(z+\alpha |\tau )}{\theta _1(z|\tau ) \theta _1(\alpha |\tau )} , \nonumber \\ \theta _1(z|\tau )&:=2 q^{1/8} \sin (\pi z) \prod _{n=1}^{\infty } (1-q^n) (1-e^{2\pi i z} q^n) (1-e^{-2\pi i z} q^n) , \ \ \ \ q:=e^{2\pi i \tau } , \end{aligned}$$

where $F(z,\alpha | \tau )$ is known as the Kronecker–Eisenstein series. The modular parameter $\tau \in {\mathbb {H}}$ of the torus takes values in the upper half plane ${\mathbb {H}}:=\{ \tau \in {\mathbb {C}} , \ \Im \tau >0\}$, and $z,\alpha \in {\mathbb {C}}$ live on the universal cover of the torus ${\mathbb {C}}/({\mathbb {Z}}+\tau \mathbb Z)$. The KZB connection $J_{\textrm{KZB}}$ depends on non-commutative indeterminates *A*, *B*, and the adjoint actions of *B* in $\textrm{ad}_B F(z,\textrm{ad}_B| \tau )$ are performed after series expansion in the second argument of *F*. Note that the elliptic associators of [26, 82, 89] are obtained from (regularized) path-ordered exponentials of (A.9), integrated over the homology cycles of the torus.

The degeneration $\tau \rightarrow i\infty$ of the Kronecker–Eisenstein series and its expansion coefficients w.r.t. the second argument $\alpha = \textrm{ad}_B$ is well-known to yield [96]

A.10 $$\begin{aligned} \lim _{\tau \rightarrow i\infty }F(z,\alpha | \tau ) = \frac{1}{\alpha }+ \pi \cot (\pi z) -2 \sum _{n=1}^{\infty } \alpha ^{2n-1} \zeta _{2n} . \end{aligned}$$

The limit $\tau \rightarrow i\infty$ degenerates the torus to a nodal sphere. In the coordinate $\sigma :=e^{2\pi i z}$ of the nodal sphere, the pinched homology cycle of the degenerate torus translates into the identification of the points $\sigma =0$ with $\sigma =\infty$. Based on $dz = \frac{d\sigma }{2\pi i \sigma }$ and (A.10), the degeneration of the KZB connection (A.9) is readily found to be

A.11 $$\begin{aligned} \lim _{\tau \rightarrow i\infty } J_{\textrm{KZB}}(A,B;z|\tau ) = \bigg \{ A+2\pi i\bigg ({-}\frac{1}{2}+ \frac{\sigma }{\sigma -1} \bigg ) [B,A] + \sum _{n=1}^{\infty } (2\pi i)^{2n} \frac{\textrm{B}_{2n}}{(2n)!} \textrm{ad}_B^{2n}A \bigg \} \frac{d\sigma }{2\pi i \sigma } . \end{aligned}$$

In order to make contact with the images $t_{01}$ and $t_{12}$ in (5.30) of the genus-zero generators *x*, *y*, we redefine the non-commutative *A*, *B* in (A.9) in terms of the generators *a*, *b* introduced in Sect. 5.1

A.12 $$\begin{aligned} A = - 2\pi i a , \ \ \ \ B= \frac{b}{2\pi i} \end{aligned}$$

and obtain the (modified) KZ connection (A.2) at $x=t_{01}$ and $y= -t_{12}$ from the degeneration (A.11),

A.13 $$\begin{aligned} \lim _{\tau \rightarrow i\infty } J_{\textrm{KZB}}(A,B;z|\tau ) = \bigg ( \frac{t_{01}}{\sigma }+ \frac{-t_{12}}{1-\sigma } \bigg ) d\sigma = J(t_{01},-t_{12};\sigma ) . \end{aligned}$$

### Link between genus zero and genus one

The non-commutative arguments $t_{01},t_{12}$ obtained in the comparison (A.13) of KZ and KZB connections do not yet line up with (5.29) and differ by a swap of *x* and *y*. This can be fixed by an additional change of coordinates to $\eta =1-\sigma$ in the degeneration of the KZB connection which is in fact necessary to map the origin $z=0$ of the torus to the origin $\eta =0$ of the nodal sphere (as opposed to $\sigma =1$). In this way, the homotopy deformation of the contour ${{\mathcal {C}}}_y$ of Fig. 3 producing the action of zeta generators in genus zero is the image of the *A*-cycle of the torus $z \in (0,1)$ under the change of variables from *z* via $\sigma =e^{2\pi i z}$ to $\eta =1-\sigma$, see Fig. 4. Similar homotopy deformations of paths together with the degeneration (A.13) of the KZB connection were used by Enriquez to express the limit $\tau \rightarrow i \infty$ of elliptic associators in terms of $\Phi _{\textrm{KZ}}$ [83].

With the degenerate KZB connection in the coordinate $\eta =1-\sigma$

A.14 $$\begin{aligned} \lim _{\tau \rightarrow i\infty } J_{\textrm{KZB}}(A,B;z|\tau ) = \bigg ( \frac{t_{12}}{\eta }+ \frac{-t_{01}}{1-\eta } \bigg ) d\eta = J(t_{12},-t_{01};\eta ) , \end{aligned}$$

we obtain the factor

A.15 $$\begin{aligned} g_w(t_{12},-t_{01}) = \textrm{Pexp} \bigg ( \int ^1_0 \bigg [ \frac{t_{12}}{\eta } + \frac{-t_{01}}{1-\eta } \bigg ] d\eta \bigg ) \, \big |_{\zeta _w} \end{aligned}$$

in the action (5.42) of genus-one zeta generators on $t_{01}$, in direct analogy with (A.2) in genus zero. Moreover, the realization (A.5) of $D_{g_w}(y)$ in genus zero generalizes to

A.16 $$\begin{aligned} \tau _w(t_{01})&= - \textrm{Pexp} \bigg ( \int _{{{\mathcal {C}}}_y} \bigg [ \frac{t_{12}}{\eta } + \frac{-t_{01}}{1-\eta } \bigg ]d\eta \bigg ) \, \big |_{2\pi i \zeta _w} , \nonumber \\&= - \lim _{\tau \rightarrow i \infty } \textrm{Pexp} \bigg ( \int _{0}^1 J_{\textrm{KZB}}(A,B;z|\tau ) \bigg ) \, \big |_{2\pi i \zeta _w} , \end{aligned}$$

i.e. the interpretation as a linearized monodromy passes through from genus zero to genus one. The loop ${{\mathcal {C}}}_y$ anchored at the tangential base point from $\eta =0$ to 1 on the sphere around the point $\eta =1$ descends from the *A*-cycle $z \in (0,1)$ of the torus. The other part $\tau _w(t_{12})=0$ of the action (5.42) of genus-one zeta generators in turn follows from a loop around the origin of both the sphere ($\eta =0$) and the torus ($z=0$) which can be contracted to an infinitesimal circle and does not produce any odd zeta values through its periods, see (A.6).

In summary, this appendix derived the close analogy between the actions (A.4) and (5.42) of zeta generators in genus zero and one and justified the morphism (5.29) by comparing (i) the underlying connections of KZ- and KZB-type in the degeneration of the torus to a nodal sphere and (ii) integration contours on the respective surfaces (loops around marked points and the pinched homology cycle of the degenerate torus).

## Appendix B. Brief Recap of Elliptic MZVs

We shall here review the definition and basic properties of elliptic MZVs, with particular emphasis on their interplay with the algebra $\mathfrak {u}$ of derivations of $\textrm{Lie}[a,b]$ in Definition 5.1.2.

Elliptic MZVs were introduced in the work of Enriquez [26, 83] as coefficients of iterated integrals over KZB connections. In particular, Enriquez’ *A*- *B*-elliptic MZVs are expansion coefficients of elliptic associators (obtained from the regularized *A*- and *B*-cycle holonomies of the KZB connection (A.9) [26, 82, 89]) in the same way as multizetas at genus zero are coefficients of the KZ associator [23]. We will restrict the review of this appendix to the discussion of *A*-elliptic MZVs which generate *B*-elliptic MZVs through their modular S transformation $\tau \rightarrow -\frac{1}{\tau }$, see [97–99] for properties specific to *B*-elliptic MZVs. For further background on elliptic MZVs, the reader is for instance referred to [100] for a comprehensive overview, to [84, 85] for their algebraic and differential relations as well as the structure of the ring they generate, and to [101, 102] for first applications to string amplitudes.

### Definition and basic properties

A convenient definition of individual *A*-elliptic MZVs involves the expansion coefficients $g^{(n)}(z| \tau )$ of the meromorphic Kronecker–Eisenstein series $F(z,\alpha | \tau )$ in (A.9) in its second argument $\alpha$,

B.1 $$\begin{aligned} F(z,\alpha | \tau ) = \sum _{n=0}^{\infty } \alpha ^{n-1} g^{(n)}(z| \tau ) , \end{aligned}$$

where $g^{(0)}(z| \tau ) = 1$. The non-constant $g^{(n)}(z| \tau )$ at $n\ge 1$ are meromorphic in both *z* in the universal cover of the torus and $\tau$ in the upper half-plane; their generating series in (A.9) yields explicit theta-function representations of $g^{(n)}(z| \tau )$ at arbitrary $n\in {\mathbb {N}}$ such as $g^{(1)}(z| \tau ) = \partial _z \log \theta _1(z|\tau )$.

#### Definition B.1.1

*A*-elliptic MZVs of length $r\ge 0$ are defined as iterated integrals of the Kronecker–Eisenstein coefficients in (B.1) over points $z_i$ on the *A*-cycle [83],

B.2 $$\begin{aligned} \omega (n_1,\ldots ,n_r|\tau ) = \int ^1_0 dz_{1}\, g^{(n_{1})}(z_{1}| \tau ) \int _{z_1}^1 dz_{2}\, g^{(n_{2})}(z_{2}| \tau ) \ldots \int ^1_{z_{r-1}} dz_r\, g^{(n_r)}(z_r| \tau ) . \end{aligned}$$

Following the enumeration conventions of [101], they are specified by integers $n_1,\ldots ,n_r \ge 0$ that specify their integration kernels. The endpoint divergences of the integrals (B.2) with $n_1 = 1$ and/or $n_r=1$ are regularized according to the prescription in [101] that is tailored to preserve the properties (i) and (ii) below of the convergent cases.

#### Proposition B.1.2

(see [83, 84, 101]) The *A*-elliptic MZVs of Definition B.1.1 exhibit the following basic properties, where $u,v \in \{ 0,1,2,\ldots \}^\times$ are words in the integer entries $n_1,n_2,\ldots$ of (B.2):

(i) shuffle relations identical to those of multizetas in (2.4),
  B.3
  see (2.5) for the shuffle product ,
(ii) reflection relations
  B.4 $$\begin{aligned} \omega (n_1,n_2,\ldots ,n_r |\tau ) = (-1)^{n_1+n_2+\ldots +n_r}\omega (n_r,\ldots ,n_2,n_1 |\tau ) , \end{aligned}$$
(iii) Fourier expansions in non-negative powers of $q = e^{2\pi i \tau }$ with ${\mathbb {Q}}[(2\pi i)^{-1}]$-linear combinations $c_n(u)$ of multizetas as coefficients
  B.5 $$\begin{aligned} \omega (u |\tau ) = \sum _{n=0}^\infty c_n(u) q^n , \end{aligned}$$
(iv) The simplest non-constant *A*-elliptic MZVs occur at length $r=2$ with odd $n_1{+}n_2$ since (for integer $k,\ell \ge 0$)
  B.6 $$\begin{aligned} \omega (2k{+}1 |\tau )&= 0 ,&\omega (2k{+}1 ,2\ell {+}1|\tau )&= 0 , \nonumber \\ \omega (2k |\tau )&= -2 \zeta _{2k} ,&\omega (2k,2\ell |\tau )&= 2 \zeta _{2k} \zeta _{2\ell } . \end{aligned}$$

#### Proof

For *A*-elliptic MZVs with $n_1,n_r \ne 1$, i.e. convergent integrals (B.2), (i) and (ii) readily follow from general properties of iterated integrals and the alternating parity $g^{(n)}(-z|\tau )=(-1)^n g^{(n)}(z|\tau )$ of the integration kernels. The extension of (i) and (ii) to divergent cases with $n_1=1$ and/or $n_r=1$ is ensured by the regularization of endpoint divergences according to [101]. The Fourier expansion of the Kronecker–Eisenstein series [96] and its coefficients $g^{(n)}(z| \tau )$ [101] implies the statement of (iii) with coefficients $c_n(u) \in {\mathbb {C}}$; the stronger statement that $c_n(u)$ are ${\mathbb {Q}}[(2\pi i)^{-1}]$-linear combinations of multizetas can for instance be established from the degeneration formulae (A.10), (A.11) for the integration kernels or the degeneration formula of [83] for elliptic associators. Finally, the expressions of (iv) for $\omega (n_1|\tau )$ readily follow from direct integration of (B.2) for $n_1\ne 1$ together with our regularization prescription for $n_1=1$; the results of (B.6) for $\omega (n_1,n_2|\tau )$ with $n_1 {+}n_2$ even in turn are simple consequences of (i), (ii) and the expressions for $\omega (n_1|\tau )$. $\square$

### Elliptic MZVs and geometric derivations

As will be reviewed in this section, the $\tau$-dependence of *A*-elliptic MZVs is governed by the algebra $\mathfrak {u}$ of Tsunogai derivations $\epsilon _k$ and their action on the free Lie-algebra in two generators *a*, *b*, see Sect. 5.1. This link arises from the generating series of *A*-elliptic MZVs

B.7 $$\begin{aligned} \mathfrak {A}_{a,b}(\tau ) = e^{i\pi [a,b]} \sum _{r=0}^\infty (-1)^r \sum _{n_1,\ldots ,n_r=0}^\infty \omega (n_1,n_2,\ldots ,n_r|\tau ) \, \textrm{ad}_a^{n_r}(b)\ldots \textrm{ad}_a^{n_2}(b) \textrm{ad}_a^{n_1}(b)\, , \end{aligned}$$

known as the *A*-elliptic associator $\mathfrak {A}_{a,b}(\tau )$, and the appearance of holomorphic Eisenstein series $\textrm{G}_{k}(\tau )$ in its $\tau$-derivative, with normalization conventions^19^

B.8 $$\begin{aligned} \textrm{G}_{k}(\tau ) = \left\{ \begin{array}{ll} \displaystyle 2 \zeta _k + \frac{2(2\pi i)^k}{(k{-}1)!} \sum _{m,n=1}m^{k-1} q^{mn}& : \ k\ge 2 \ \textrm{even} , \\ -1 & : \ k=0 . \end{array} \right. \end{aligned}$$

#### Theorem B.2.1

(Enriquez [26, 83]). The *A*-elliptic associator $\mathfrak {A}_{a,b}(\tau )$ obeys the differential equation,

B.9 $$\begin{aligned} 2\pi i \frac{\partial }{\partial \tau } \mathfrak {A}_{a,b}(\tau ) = \sum _{\ell =0}^\infty (1{-}2\ell ) \textrm{G}_{2\ell }(\tau ) \epsilon _{2\ell } \mathfrak {A}_{a,b}(\tau ) , \end{aligned}$$

with Tsunogai derivations acting on the associator via (5.2), (5.3) and their Leibniz property.

The factor of $e^{i\pi [a,b]}$ in the expansion (B.7) of $\mathfrak {A}_{a,b}(\tau )$ ensures that regularized *A*-elliptic MZVs obey the reflection property (B.4) and does not alter the differential equation (B.9) since Tsunogai derivations $\epsilon _{2\ell }$ all annihilate the commutator [*a*, *b*].

#### Corollary B.2.2

(Broedel, Matthes, Schlotterer [84]). *A*-elliptic MZVs are given by (tangential-base-point regularized [103]) linear combinations of iterated integrals over holomorphic Eisenstein series (B.8) with ${\mathbb {Q}}[(2\pi i)^{-1}]$ linear combinations of multizetas as coefficients.

#### Proof

The discussion in section 4.3 of [84] (with *x*, *y* renamed to *a*, *b*) is equivalent to writing the *A*-elliptic associator as a path-ordered exponential in the conventions of (A.3),

B.10 $$\begin{aligned} \mathfrak {A}_{a,b}(\tau ) = \textrm{Pexp}\bigg ( \int ^\tau _{i\infty } \frac{d \tau '}{2\pi i}\sum _{\ell =0}^\infty (1{-}2\ell ) \textrm{G}_{2\ell }(\tau ') \epsilon _{2\ell } \bigg ) \mathfrak {A}_{a,b}(i\infty ) . \end{aligned}$$

The initial value $\mathfrak {A}_{a,b}(i\infty )$ is obtained from the Drinfeld associator as spelt out in section 4.5 of [82], section 1.2 of [83] and section 2.3 of [84]. $\square$

#### Remark B.2.3

*A*-elliptic MZVs only realize the proper subset of iterated Eisenstein integrals admitted by the differential equation (B.9) and the relations in $\mathfrak {u}$, namely

B.11 $$\begin{aligned} \textrm{ad}^{2k-1}_{\epsilon _0}(\epsilon _{2k}) = 0 \ \forall \ k \ge 1 , \ \ \ \ [\epsilon _2, \epsilon _{2\ell }] = 0 \ \forall \ \ell \ge 0 \end{aligned}$$

and Pollack’s relations in Remark 5.1.6. Each relation in the Lie-algebra $\mathfrak {u}$ of $\epsilon _{2\ell }$ leads to a dropout of a (shuffle-indecomposable) iterated Eisenstein integral in the expansion of (B.10).

More precisely, the dropouts of iterated Eisenstein integrals from (B.10) due to the closed-form relations among $\epsilon _{2\ell }$ in (B.11) can be described as follows:

(i) The fact that $\epsilon _2$ is central in $\mathfrak {u}$, i.e. $[\epsilon _2, \epsilon _{2\ell }] = 0$, implies that all appearances of the quasi-modular holomorphic Eisenstein series $\textrm{G}_2$ in the iterated Eisenstein integrals of *A*-elliptic MZVs can be reduced to polynomials in the depth-one integral $\int ^\tau _{i\infty } d\tau ' \, \textrm{G}_2(\tau ')$. The latter is related to the only shuffle-indecomposable divergent *A*-elliptic MZV (besides the vanishing $\omega (1|\tau )=0$) via $\omega (0,1|\tau ) = \frac{i\pi }{2} -\frac{1}{2\pi i} \int ^\tau _{i\infty } d\tau ' ( \textrm{G}_2(\tau ') - 2\zeta _2)$ [84].
(ii) The leftover iterated Eisenstein integrals over $\textrm{G}_0 = -1$ and modular $\textrm{G}_k$ at $k\ge 4$ in (B.10) are expressible as Brown’s iterated integrals over kernels $d\tau \, \tau ^j \textrm{G}_k(\tau )$ [103], where $k\ge 4$ and $0\le j \le k{-}2$ by virtue of $\textrm{ad}^{k-1}_{\epsilon _0}(\epsilon _{k}) = 0$. The coefficients in these decompositions are ${\mathbb {Q}}[(2\pi i)^{\pm 1}]$ polynomials in $\tau$ that can for instance be determined from the generating functions in [97].

The Pollack relations of Remark 5.1.6 in turn induce further dropouts among Brown’s iterated Eisenstein integrals over the above $d\tau \, \tau ^j \textrm{G}_k(\tau )$ which are realized among elliptic MZVs. For instance, $[\epsilon _4,\epsilon _{10}] = 3 [\epsilon _6,\epsilon _8]$ interlocks the double integrals over $\textrm{G}_{k_1}(\tau _1) \textrm{G}_{k_2}(\tau _2)$ with $(k_1,k_2) = (4,10), (6,8),(8,6), (10,4)$ such that only three ${\mathbb {Q}}$-linear combinations occur among *A*-elliptic MZVs. Similarly, the relation (5.19) interlocks triple integrals (e.g. over $\textrm{G}_4(\tau _1) \textrm{G}_4(\tau _2) \textrm{G}_8(\tau _3)$) with double integrals (e.g. over $\tau _1 \textrm{G}_8(\tau _1) \textrm{G}_8(\tau _2)$).

### Elliptic MZVs versus modular graph forms

We conclude this section with comments on links between elliptic MZVs and the non-holomorphic modular graph forms [13, 14] of closed-string amplitudes. The main statement is that the one-to-one correspondence between relations in $\mathfrak {u}$ and dropouts of iterated Eisenstein integral from *A*-elliptic MZVs extends to modular graph forms.

An alternative way of constructing generating series of all *A*-elliptic MZVs akin to the associator is to expand the configuration-space integrals in genus-one open-string amplitudes in certain physical parameters [104, 105]. The generating series of open-string integrals in the references over different numbers *n* of marked points obey the differential equation (B.9) of the *A*-elliptic associator with conjectural $(n{-}1)!\times (n{-}1)!$ matrix representations of the $\epsilon _{2\ell }$. In particular, the expansion of open-string integrals in terms of *A*-elliptic MZVs arises from the analogous matrix representations of the path-ordered exponential in (B.10) and therefore features a dropout among the iterated Eisenstein integrals for each relation among the matrix representations of $\epsilon _{2\ell }$.

Similar generating series of configuration-space integrals adapted to closed-string (rather than open-string) amplitudes at genus one generate all modular graph forms [106, 107]. The holomorphic differential equations of these closed-string integrals in $\tau$ again take the form of (B.9) with almost identical conjectural matrix representations of $\epsilon _{2\ell }$ as seen in the open-string case. Hence, the matrix representation of the path-ordered exponential in (B.10)—along with the dropouts of iterated Eisenstein integrals for each relation in $\mathfrak {u}$—also features in generating series of modular graph forms.

More precisely, modular graph forms are modular combinations of iterated Eisenstein integrals, their complex conjugates, multizetas and rational functions of $\tau ,{\bar{\tau }}$ [11, 13, 97, 107]. Accordingly, their generating series combine the holomorphic path-ordered exponential (B.10) with its complex conjugate (reversed in the concatenation order of the $\epsilon _{2\ell }$) and series in multizetas as in Brown’s construction of equivariant iterated Eisenstein integrals [9, 10]. The identification of Brown’s construction with generating series of modular graph forms is described in [11], and the description of the series in multizetas via zeta generators can be found in the companion paper [12] to this work.

In conclusion, the dropouts of elliptic MZVs due to relations in $\mathfrak {u}$ directly carry over to modular graph forms under the following assumption: the matrices in the generating series of open- and closed-string genus-one integrals in [104–107] need to obey the entirety of all relations among geometric derivations $\epsilon _{2\ell }$ as supported by the arguments in section 4.5 of [105]. Independently on this assumption, the generating series of equivariant iterated Eisenstein integrals of [9, 10] exhibits one dropout of non-holomorphic modular form per relation in $\mathfrak {u}$.

## Footnotes

1 The definition of motivic multizeta values originates from algebro-geometric considerations as explained for example in [1–3].

2 More formally, this is the Lie algebra of the pro-unipotent radical of the fundamental group of the Tannakian category of mixed Tate motives unramified over $\mathbb {Z}$ [19].

3 The Lie algebra $\mathfrak {u}$ is not free on the $\epsilon _k^{(j)}$ but satisfies relations [30–32] that are not homogeneous in modular depth and which, for this reason, only provides a filtration rather than a grading of $\mathfrak {u}$, see Remark 5.1.6.

4 The coaction $\Delta ^{GB}$ based on Goncharov’s original coproduct was introduced in [1, 2, 34]. The coaction $\Delta _{GB}$ is used in the recent particle-physics, string-theory and mathematics literature such as [61–66].

5 The superscript in $\zeta ^{\mathfrak {dr}}$ refers to de Rham periods [1–3, 69]. In the motivic coaction $\Delta _{GB}$, de Rham periods occur in the right entry of tensor products $A \otimes B$, i.e. *B* is considered modulo $i\pi$. This is opposite to the coaction $\Delta ^{GB}$ of [2] where de Rham periods are in the left entry, so that (2.46) would instead read $\Delta ^{GB}(\zeta ^{\mathfrak {m}}_2)=1\otimes \zeta ^{\mathfrak {m}}_2$.

6 The subscript “KZ” in $\Phi _{\textrm{KZ}}(x,y)$ stems from the fact that the Drinfeld associator can be constructed by solving the Knizhnik–Zamolodchikov equation, see “ 7.4.4”.

7 This definition gives an a posteriori explanation of the stuffle-regularized MZVs defined in (2.12): for words *w* ending with *y* the value $\zeta _*(w)$ is nothing other than the coefficient of *w* in the product $C \Phi$ of formal power series, where *C* is the power series defined in (2.10).

8 The product $\circ$ among $h\in {\mathfrak {ds}}$ and $g\in {\mathcal {U}}{\mathfrak {ds}}$ in [62] is related to the Poincaré–Birkhoff–Witt multiplication $\diamond$ in (3.17) via $\overleftarrow{g \circ h}: = \overleftarrow{h} \diamond \overleftarrow{g}$, where $\overleftarrow{w}$ is obtained by reversing the letters *x*, *y* of $w \in {\mathbb {Q}}\langle x,y \rangle$. This is a consequence of $D_{\overleftarrow{h}}(\overleftarrow{g}) = -\overleftarrow{D_h (g)}$ which can be proven by induction. The Drinfeld associator in the conventions of [62] is obtained from the series $\Phi (x,y)$ in the present work by reversing the words $w \mapsto \overleftarrow{w}$ in (3.42).

9 The minus sign in front of $t_{01}$ in (5.32) is present because if $h(x,y)\in {\mathfrak {mz}}^\vee \subset {\mathfrak {ds}}$, then as in (5.24), the polynomial $h(x,-y)$ lies in ${\mathfrak {grt}}$. Since the process described in the present section is an explicit version of Enriquez’s map (5.25) from ${\mathfrak {grt}}$ to $\textrm{Der}^0\textrm{Lie}[a,b]$, the starting point of the map is the ${\mathfrak {grt}}$ polynomial $h(x,-y)$, or more precisely, the associated Ihara derivation which maps $x\mapsto 0$ and $y\mapsto [y,h(x,-y)]$. The first step in the explicit construction of the Enriquez map is transporting this Ihara derivation to a derivation on $\textrm{Lie}[t_{01},t_{12}]$ via the map (5.29), which is what is expressed in (5.32).

10 Note that, as a derivation, the lowest degree of $\sigma _3$ is 4, but here we are writing the degree of the image of *a* and *b* as the subscript.

11 It can be useful, although not necessary, to group these terms according to $\mathfrak {sl}_2$ representations.

12 This symmetry property follows from the odd degree *w* of $g_w$ together with the facts that $g_w(x,-y)\in {\mathfrak {grt}}$ by (5.24) and that one of the defining properties of elements $h\in {\mathfrak {grt}}$ is $h(x,y)+h(y,x)=0$.

13 The prefactor follows from the fact that the $\mathfrak {sl}_2$ properties of $[\epsilon _0, (\sigma _w)_{2w}^{(d)}]$ are identical to $\epsilon _{d+1}^{(\frac{d+1}{2})}$, where the action of the lowering operator $\textrm{ad}_{\epsilon _0^\vee }$ yields $\frac{1}{4}(d-1)(d+1) \epsilon _{d+1}^{(\frac{d-1}{2})}$ by (5.12).

14 The analogous highest-weight vectors in the expansion (5.49) that completely determine $\sigma _7$ are given by $-\frac{1}{720} \epsilon _8^{(6)}$ at degree 8, by $\frac{7 }{1152} ( [\epsilon _4^{(2)},\epsilon _6^{(3)}] -[\epsilon _4^{(1)},\epsilon _6^{(4)}])$ at degree 10, by $\frac{1}{13{,}824}([\epsilon _6^{(1)},\epsilon _6^{(4)}] - [\epsilon _6^{(2)},\epsilon _6^{(3)}])$ and $-\frac{661}{57{,}600} ( [\epsilon _4^{(1)},[\epsilon _4^{(1)},\epsilon _4^{(2)}]] + [\epsilon _4^{(2)},[\epsilon _4^{(2)},\epsilon _4]])$ at degree 12 and finally $z_7$ at key degree 14.

15 More specifically, Pollack relations whose relative factors in the modular-depth-two contributions $[ \epsilon _{k_1}^{(j_1)} , \epsilon _{k_2}^{(j_2)}]$ are governed by holomorphic cusp forms of modular weight *w* [32] fall into irreducible $\mathfrak {sl}_2$ representations of dimension $w-1$.

16 For instance, the image of the second relation in (5.18) under $\textrm{ad}_{\epsilon _0}^{10}$ can be used to convert contributions $\sim s^3(\epsilon _4,\epsilon _{12}),s^3(\epsilon _6,\epsilon _{10})$ and $s^3(\epsilon _8,\epsilon _{8})$ to $\sigma _{13}^{ \{2\} }$ into contributions $\sim [\epsilon _4^{(2)},[\epsilon _4^{(2)},\epsilon _8^{(6)}]]$ and $[\epsilon _6^{(4)},[\epsilon _6^{(4)},\epsilon _4^{(2)}]]$ to $\sigma _{13}^{ \{3\} }$. Similarly, the coefficient of $t^4(\epsilon _4, \epsilon _{14})$ in $[z_3, \epsilon _{12}]^{\{2\}}$ can be modified through Pollack relations of degree 18 at the cost of extra terms in all of $[z_3, \epsilon _{12}]^{\{2\}}$, $[z_3, \epsilon _{12}]^{\{3\}}$ and $[z_3, \epsilon _{12}]^{\{4\}}$.

17 The analogous highest-weight vectors in $\sigma _9^{\{2\}}$ resulting from the first line of (7.18) are given by $\frac{1}{5184}([\epsilon _4^{(2)},\epsilon _8^{(5)}] - [\epsilon _4^{(1)},\epsilon _8^{(6)}])$ and $-\frac{7 }{20{,}736}[\epsilon _6^{(3)},\epsilon _6^{(4)}]$ at degree 12, $\frac{7 }{4{,}147{,}200} ( [\epsilon _6^{(1)},\epsilon _8^{(6)}] - [\epsilon _6^{(2)},\epsilon _8^{(5)}] +[\epsilon _6^{(3)},\epsilon _8^{(4)}]-[\epsilon _6^{(4)},\epsilon _8^{(3)}])$ at degree 14 and $-\frac{1}{26{,}127{,}360}([\epsilon _8^{(1)},\epsilon _8^{(6)}]-[\epsilon _8^{(2)},\epsilon _8^{(5)}]+[\epsilon _8^{(3)},\epsilon _8^{(4)}])$ at degree 16.

## References

[1] Goncharov, A.: Galois symmetries of fundamental groupoids and noncommutative geometry. Duke Math. J. **128**, 209 (2005). arXiv:math/0208144 [math.AG]

[2] Brown, F.: Mixed Tate motives over . Ann. Math. **175**(2), 949–976 (2012). arXiv:1102.1312 [math.AG]

[3] Brown, F.: Motivic periods and the projective line minus three points. In: Proceedings of the ICM 2014 (2014). arXiv:1407.5165 [math.NT]. https://api.semanticscholar.org/CorpusID:118359180

[4] Brown, F.: On the decomposition of motivic multiple zeta values. In: Galois–Teichmüller Theory and Arithmetic Geometry, vol. 63 of Advanced Studies in Pure Mathematics, pp. 31–58. Mathematical Society of Japan, Tokyo (2012). arXiv:1102.1310 [math.NT]

[5] Brown, F.: Polylogarithmes multiples uniformes en une variable. C. R. Acad. Sci. Paris Ser. **I**(338), 527–532 (2004)

[6] Broedel, J., Sprenger, M., Torres Orjuela, A.: Towards single-valued polylogarithms in two variables for the seven-point remainder function in multi-Regge-kinematics. Nucl. Phys. B **915**, 394–413 (2017). arXiv:1606.08411 [hep-th]

[7] Del Duca, V., Druc, S., Drummond, J., Duhr, C., Dulat, F., Marzucca, R., Papathanasiou, G., Verbeek, B.: Multi-Regge kinematics and the moduli space of Riemann spheres with marked points. JHEP **08**, 152 (2016). arXiv:1606.08807 [hep-th]

[8] Frost, H., Hidding, M., Kamlesh, D., Rodriguez, C., Schlotterer, O., Verbeek, B.: Motivic coaction and single-valued map of polylogarithms from zeta generators. J. Phys. A **57**(31), 31LT01 (2024). arXiv:2312.00697 [hep-th]

[9] Brown, F.: A class of non-holomorphic modular forms I. Res. Math. Sci. **5**(5), 7 (2018). arXiv:1707.01230 [math.NT]

[10] Brown, F.: A class of non-holomorphic modular forms II: equivariant iterated Eisenstein integrals. Forum Math. Sigma **8**, 1 (2020). arXiv:1708.03354 [math.NT]

[11] Dorigoni, D., Doroudiani, M., Drewitt, J., Hidding, M., Kleinschmidt, A., Matthes, N., Schlotterer, O., Verbeek, B.: Modular graph forms from equivariant iterated Eisenstein integrals. JHEP **12**, 162 (2022). arXiv:2209.06772 [hep-th]

[12] Dorigoni, D., Doroudiani, M., Drewitt, J., Hidding, M., Kleinschmidt, A., Schlotterer, O., Schneps, L., Verbeek, B.: Non-holomorphic modular forms from zeta generators. JHEP **10**, 053 (2024). arXiv:2403.14816 [hep-th] 10.1007/s00220-025-05489-x PMC12686056 41376673

[13] D’Hoker, E., Green, M.B., Gürdogan, Ö., Vanhove, P.: Modular graph functions. Commun. Number Theor. Phys. **11**, 165–218 (2017). arXiv:1512.06779 [hep-th]

[14] D’Hoker, E., Green, M.B.: Identities between modular graph forms. J. Number Theory **189**, 25–80 (2018). arXiv:1603.00839 [hep-th]

[15] Gerken, J.E.: Modular graph forms and scattering amplitudes in string theory. arXiv:2011.08647 [hep-th]

[16] Berkovits, N., D’Hoker, E., Green, M.B., Johansson, H., Schlotterer, O.: Snowmass white paper: string perturbation theory. In: 2022 Snowmass Summer Study, vol. 3 (2022). arXiv:2203.09099 [hep-th]

[17] D’Hoker, E., Kaidi, J.: Lectures on modular forms and strings. arXiv:2208.07242 [hep-th]

[18] Levine, M.: Tate motives and the vanishing conjectures for algebraic -theory. In: Algebraic -Theory and Algebraic Topology (Lake Louise, AB, 1991), vol. 407 of NATO Advanced Study Institute on Mathematical Physics, pp. 167–188. Kluwer Academic Publishers, Dordrecht (1993)

[19] Deligne, P., Goncharov, A.B.: Groupes fondamentaux motiviques de tate mixte. Ann. Sci. lÉcole Normale Supérieure **38**(1), 1–56 (2005). https://www.sciencedirect.com/science/article/pii/S0012959305000029

[20] Schneps, L.: Dual-depth adapted irreducible formal multizeta values. Math. Scand. **113**(1), 53–62 (2013)

[21] Drinfeld, V.: Quasi Hopf algebras. Leningrad Math. J. **1**, 1419–1457 (1989)

[22] Drinfeld, V.: On quasitriangular quasi-Hopf algebras and on a group that is closely connected with . Leningrad Math. J. **2**(4), 829–860 (1991)

[23] Le, T.T.Q., Murakami, J.: Kontsevich’s integral for the Kauffman polynomial. Nagoya Math. J. **142**, 39–65 (1996)

[24] Écalle, J.: ARI/GARI, la dimorphie et l’arithmétique des multizêtas:un premier bilan. J. Theory Nombres Bordeaux **15**(2), 411–478 (2003)

[25] Keilthy, A.: Rational structures on multiple zeta values. PhD thesis, University of Oxford (2020). https://ora.ox.ac.uk/objects/uuid:f46cf1e1-f5d7-45c8-b8c3-c55b3caf139f/files/dxs55mc143

[26] Enriquez, B.: Elliptic associators. Sel. Math. (N.S.) **20**(2), 491–584 (2014). arXiv:1003.1012 [math.QA]

[27] Hain, R., Matsumoto, M.: Universal mixed elliptic motives. J. Inst. Math. Jussieu **19**(3), 663–766 (2020). arXiv:1512.03975 [math.AG]

[28] Écalle, J.: The flexion structure and dimorphy: flexion units, singulators, generators, and the enumeration of multizeta irreducibles. CRM Ser. **12**, 27–211 (2011)

[29] Schneps, L.: Elliptic double shuffle, Grothendieck–Teichmüller and mould theory. Ann. Math. Québec **44**(2), 261–289 (2020). arXiv:1506.09050 [math.NT]

[30] Ihara, Y.: Some arithmetic aspects of Galois actions in the pro- fundamental group of . In: Arithmetic Fundamental Groups and Noncommutative Algebra (Berkeley, CA, 1999), vol. 70 of Proceedings of a Symposium in Pure Mathematics, pp. 247–273. American Mathematical Society, Providence, RI (2002)

[31] Schneps, L.: On the Poisson bracket on the free Lie algebra in two generators. J. Lie Theory **16**(1), 19–37 (2006)

[32] Pollack, A.: Relations between derivations arising from modular forms. Undergraduate thesis, Duke University (2009). https://dukespace.lib.duke.edu/dspace/handle/10161/1281

[33] Brown, F.: Zeta elements in depth 3 and the fundamental lie algebra of the infinitesimal Tate curve. Forum Math. Sigma **5** (2017). arXiv:1504.04737 [math.NT]

[34] Goncharov, A.B.: Multiple polylogarithms and mixed Tate motives. arXiv:math/0103059 [math.AG]

[35] Zagier, D.: The Bloch–Wigner–Ramakrishnan polylogarithm function. Math. Ann. **286**, 613 (1990)

[36] D’Hoker, E., Green, M.B., Pioline, B.: Asymptotics of the genus-two string invariant. Commun. Number Theory Phys. **13**(2), 351–462 (2019). arXiv:1806.02691 [hep-th]

[37] Basu, A.: Poisson equations for elliptic modular graph functions. Phys. Lett. B **814**, 136086 (2021). arXiv:2009.02221 [hep-th]

[38] D’Hoker, E., Kleinschmidt, A., Schlotterer, O.: Elliptic modular graph forms. Part I. Identities and generating series. JHEP **03**, 151 (2021). arXiv:2012.09198 [hep-th]

[39] Hidding, M., Schlotterer, O., Verbeek, B.: Elliptic modular graph forms II: iterated integrals. arXiv:2208.11116 [hep-th]

[40] Enriquez, B.: Flat connections on configuration spaces and braid groups of surfaces. Adv. Math. **252**, 204–226 (2014). arXiv:1112.0864 [math.GT]

[41] Enriquez, B., Zerbini, F.: Construction of Maurer–Cartan elements over configuration spaces of curves. arXiv:2110.09341 [math.AG]

[42] Enriquez, B., Zerbini, F.: Analogues of hyperlogarithm functions on affine complex curves. arXiv:2212.03119 [math.AG]

[43] D’Hoker, E., Hidding, M., Schlotterer, O.: Constructing polylogarithms on higher-genus Riemann surfaces. Commun. Number Theory Phys. **19**(2), 355–413 (2025). arXiv:2306.08644 [hep-th]

[44] D’Hoker, E., Green, M.B.: Zhang–Kawazumi invariants and superstring amplitudes. J. Number Theory **144**, 111 (2014). arXiv:1308.4597 [hep-th]

[45] Pioline, B.: A Theta lift representation for the Kawazumi–Zhang and Faltings invariants of genus-two Riemann surfaces. J. Number Theory **163**, 520–541 (2016). arXiv:1504.04182 [hep-th]

[46] D’Hoker, E., Green, M.B., Pioline, B.: Higher genus modular graph functions, string invariants, and their exact asymptotics. Commun. Math. Phys. **366**(3), 927–979 (2019). arXiv:1712.06135 [hep-th]

[47] Basu, A.: Eigenvalue equation for genus two modular graphs. JHEP **02**, 046 (2019). arXiv:1812.00389 [hep-th]

[48] Kawazumi, N.: Lecture “Some tensor field on the Teichmüller space” given at MCM2016, OIST (2016). https://www.ms.u-tokyo.ac.jp/~kawazumi/OIST1610_v1.pdf

[49] Kawazumi, N.: Lecture “Differential forms and functions on the moduli space of Riemann surfaces” given in the “Séminaire Algèbre et topologie, Université de Strasbourg” (2017). https://www.ms.u-tokyo.ac.jp/~kawazumi/1701Strasbourg_v1.pdf

[50] D’Hoker, E., Schlotterer, O.: Identities among higher genus modular graph tensors. Commun. Number Theory Phys. **16**(1), 35–74 (2022). arXiv:2010.00924 [hep-th]

[51] Kawazumi, N.: A twisted invariant of a compact Riemann surface. arXiv:2210.00532 [math.GT]

[52] Gil, J.I.B., Fresan, J.: Multiple zeta values: from numbers to motives. In: Clay Mathematics Proceedings, to appear. http://javier.fresan.perso.math.cnrs.fr/mzv.pdf

[53] Schneps, L.: ARI, GARI, Zig and Zag: an introduction to Écalle’s theory of multizeta values. arXiv:1507.01534 [math.NT]

[54] Ihara, K., Kaneko, M., Zagier, D.: Derivation and double shuffle relations for multiple zeta values. Compos. Math. **142**(2), 307–338 (2006)

[55] Furusho, H.: The multiple zeta value algebra and the stable derivation algebra. Publ. Res. Inst. Math. Sci. **39**(4), 695–720 (2003) https://ems.press/content/serial-article-files/40837

[56] Hoffman, M.E.: Quasi-shuffle products. J. Algebraic Comb. **11**, 49–68 (2000). arXiv:9907173v1

[57] Racinet, G.: Doubles mélanges des polylogarithmes multiples aux racines de l?unité. Publications Math. l’IHÉS **95**, 185–231 (2002) http://eudml.org/doc/104182

[58] Burmester, A., Confurius, N., Kühn, U.: AGZT-Lectures on formal multiple zeta values. arXiv:2406.13630 [math.NT]

[59] Espie, M., Novelli, J.-C., Racinet, G.: Formal Computations about Multiple Zeta Values, pp. 1–16. De Gruyter, Berlin (2003)

[60] Hoffman, M.E.: Multiple harmonic series. Pac. J. Math. **152**(2), 275–290 (1992). http://projecteuclid.org/euclid.pjm/1102636166

[61] Duhr, C.: Hopf algebras, coproducts and symbols: an application to Higgs boson amplitudes. JHEP **08**, 043 (2012). arXiv:1203.0454 [hep-ph]

[62] Drummond, J.M., Ragoucy, E.: Superstring amplitudes and the associator. JHEP **08**, 135 (2013). arXiv:1301.0794 [hep-th]

[63] Brown, F., Dupont, C.: Single-valued integration and double copy. J. Reine Angew. Math. **2021**(775), 145–196 (2021). arXiv:1810.07682 [math.NT]

[64] Brown, F., Dupont, C.: Lauricella hypergeometric functions, unipotent fundamental groups of the punctured Riemann sphere, and their motivic coactions. Nagoya Math. J. **249**, 148–220 (2023) arXiv:1907.06603 [math.AG]

[65] Abreu, S., Britto, R., Duhr, C.: The SAGEX review on scattering amplitudes Chapter 3: mathematical structures in Feynman integrals. *J. Phys. A* **55**(44), 443004 (2022). arXiv:2203.13014 [hep-th]

[66] Mafra, C.R., Schlotterer, O.: Tree-level amplitudes from the pure spinor superstring. Phys. Rep. **1020**, 1–162 (2023). arXiv:2210.14241 [hep-th]

[67] Deligne, P.: Multizêtas, according to Francis Brown. In: Bourbaki Seminar Volume 2011/2012 Exhibits 1043–1058, no. 352 in Asterisk, pp. 161–185. French Mathematical Society (2013). https://www.numdam.org/item/AST_2013__352__161_0/

[68] Dupont, C.: An introduction to mixed Tate motives. arXiv:2404.03770 [math.AG]

[69] Brown, F.: Notes on motivic periods. Commun. Number Theory Phys. **11**(3), 557–655 (2017). arXiv:1512.06410 [math.NT]

[70] Lyndon, R.: Free differential calculus. IV: the quotient groups of the lower central series. Ann. Math. **68**(2), 81–95 (1958)

[71] Širšov, A.I.: On free Lie rings. Mat. Sb. (N.S.) **45**(87), 113–122 (1958)

[72] Reutenauer, C.: Free Lie Algebras. London Mathematical Society Monographs. New Series. The Clarendon Press, vol. 7. Oxford University Press, New York (1993)

[73] Perrin, D., Viennot, G.: A note on shuffle algebras (1981). Unpublished note, personal communication

[74] Milnor, J.W., Moore, J.C.: On the structure of Hopf algebras. Ann. Math. (2) **81**, 211–264 (1965)

[75] Bourbaki, N.: Lie Groups and Lie Algebras. Chapters 1–3. Elements of Mathematics (Berlin). Springer, Berlin (1998). Translated from the French, Reprint of the 1989 English translation

[76] Blümlein, J., Broadhurst, D.J., Vermaseren, J.A.M.: The multiple zeta value data mine. Comput. Phys. Commun. **181**, 582–625 (2010). arXiv:0907.2557 [math-ph]

[77] Schnetz, O.: HyperlogProcedures. Maple procedures available on the homepage of the author (2023). https://www.math.fau.de/person/oliver-schnetz/

[78] Schneps, L.: Double shuffle and Kashiwara–Vergne Lie algebras. J. Algebra **367**, 54–74 (2012). arXiv:1201.5316 [math.QA]

[79] Tsunogai, H.: On some derivations of Lie algebras related to Galois representations. Publ. Res. Inst. Math. Sci. **31**(1), 113–134 (1995)

[80] Tsunogai, H.: The stable derivation algebras for higher genera. Israel J. Math. **136**, 221–250 (2003)

[81] Ihara, Y.: Braids, Galois groups, and some arithmetic functions. In: Proceedings of the International Congress of Mathematicians, Vol. I, II (Kyoto, 1990), pp. 99–120. Mathematical Society of Japan, Tokyo (1991)

[82] Calaque, D., Enriquez, B., Etingof, P.: Universal KZB equations: the elliptic case. In: Algebra, Arithmetic, and Geometry: In Honor of Yu. I. Manin. Vol. I, vol. 269 of Progress in Mathematics, pp. 165–266. Birkhäuser Boston, Boston (2009). arXiv:math/0702670

[83] Enriquez, B.: Analogues elliptiques des nombres multizétas. Bull. Soc. Math. France **144**(3), 395–427 (2016). arXiv:1301.3042 [math.NT]

[84] Broedel, J., Matthes, N., Schlotterer, O.: Relations between elliptic multiple zeta values and a special derivation algebra. J. Phys. A **49**(15), 155203 (2016). arXiv:1507.02254 [hep-th]

[85] Lochak, P., Matthes, N., Schneps, L.: Elliptic multizetas and the elliptic double shuffle relations. Int. Math. Res. Not. **2021**, 695–753 (2021). arXiv:1703.09410 [math.NT]

[86] Luque, J.-G., Novelli, J.-C., Thibon, J.-Y.: Period polynomials and Ihara brackets. J. Lie Theory **17**, 229–239 (2007). arXiv:math/0606301 [math.CO,math.NT]

[87] Grothendieck, A., Raynaud, M.: Revêtements Étales et Groupe Fondamental (SGA1). Lecture Notes in Mathematics, vol. 224. Springer, Berlin (1971)

[88] André, Y.: Une introduction aux motifs (motifs purs, motifs mixtes, périodes). Panoramas et Synthèses [Panoramas and Syntheses], vol. 17. Société Mathématique de France, Paris (2004)

[89] Hain, R.: Notes on the universal elliptic KZB connection. Pure Appl. Math. Q. **16**(2), 229–312 (2020). arXiv:1309.0580 [math.AG]

[90] Raphael, E., Schneps, L.: On linearised and elliptic versions of the Kashiwara–Vergne Lie algebra. arXiv:1706.08299v1 [math.QA]

[91] Écalle, J.: Eupolars and their bialternality grid. Acta Vietnam. **40**, 545–636 (2015)

[92] Baumard, S., Schneps, L.: On the derivation representation of the fundamental Lie algebra of mixed elliptic motives. Ann. Math. Qué. **41**(1), 43–62 (2017). arXiv:1510.05549 [math.QA]

[93] Dorigoni, D., Kleinschmidt, A., Schlotterer, O.: Poincaré series for modular graph forms at depth two. Part I. Seeds and Laplace systems. JHEP **01**, 133 (2022). arXiv:2109.05017 [hep-th]

[94] Dorigoni, D., Kleinschmidt, A., Schlotterer, O.: Poincaré series for modular graph forms at depth two. Part II. Iterated integrals of cusp forms. JHEP **01**, 134 (2022). arXiv:2109.05018 [hep-th]

[95] Brown, F., Levin, A.: Multiple elliptic polylogarithms. arXiv:1110.6917 [math]

[96] Zagier, D.: Periods of modular forms and Jacobi theta functions. Invent. Math. **104**(3), 449–465 (1991)

[97] Broedel, J., Schlotterer, O., Zerbini, F.: From elliptic multiple zeta values to modular graph functions: open and closed strings at one loop. JHEP **01**, 155 (2019). arXiv:1803.00527 [hep-th]

[98] Zerbini, F.: Elliptic multiple zeta values, modular graph functions and genus 1 superstring scattering amplitudes. PhD thesis, Bonn U. (2017). arXiv:1804.07989 [math-ph]

[99] Zerbini, F.: Modular and holomorphic graph function from superstring amplitudes. In: KMPB Conference: Elliptic Integrals, Elliptic Functions and Modular Forms in Quantum Field Theory Zeuthen, Germany, October 23–26, 2017 (2018). arXiv:1807.04506 [math-ph]

[100] Matthes, N.: Elliptic Multiple Zeta Values. PhD thesis, Universität Hamburg (2016)

[101] Broedel, J., Mafra, C.R., Matthes, N., Schlotterer, O.: Elliptic multiple zeta values and one-loop superstring amplitudes. JHEP **07**, 112 (2015). arXiv:1412.5535 [hep-th]

[102] Broedel, J., Matthes, N., Richter, G., Schlotterer, O.: Twisted elliptic multiple zeta values and non-planar one-loop open-string amplitudes. J. Phys. A **51**(28), 285401 (2018). arXiv:1704.03449 [hep-th]

[103] Brown, F.: Multiple modular values and the relative completion of the fundamental group of . arXiv:1407.5167 [math.NT]

[104] Mafra, C.R., Schlotterer, O.: All-order alpha’-expansion of one-loop open-string integrals. Phys. Rev. Lett. **124**(10), 101603 (2020). arXiv:1908.09848 [hep-th] 32216382 10.1103/PhysRevLett.124.101603

[105] Mafra, C.R., Schlotterer, O.: One-loop open-string integrals from differential equations: all-order ’-expansions at points. JHEP **03**, 007 (2020). arXiv:1908.10830 [hep-th] 10.1103/PhysRevLett.124.101603 32216382

[106] Gerken, J.E., Kleinschmidt, A., Schlotterer, O.: All-order differential equations for one-loop closed-string integrals and modular graph forms. JHEP **01**, 064 (2020). arXiv:1911.03476 [hep-th]

[107] Gerken, J.E., Kleinschmidt, A., Schlotterer, O.: Generating series of all modular graph forms from iterated Eisenstein integrals. JHEP **07**(07), 190 (2020). arXiv:2004.05156 [hep-th]

